# Supplementary material for: Reducing cardiovascular disease risk among families with familial hypercholesterolaemia by improving diet and physical activity: a randomised controlled feasibility trial
Source: BMJ Open. 2020 Dec 28;10(12):e044200. doi: 10.1136/bmjopen-2020-044200 (PMC7772289; doi:10.1136/bmjopen-2020-044200)
Supplement: Supplementary data [file bmjopen-2020-044200supp001.pdf]

Supplementary file 1. CONSORT 2010 checklist of information to include when reporting a pilot or feasibility trial

Section/Topic	Item No	Checklist item	Reported on page #
**Title and abstract**			
	1a	Identification as a pilot or feasibility randomised trial in the title	Title
	1b	Structured summary of pilot trial design, methods, results, and conclusions (for specific guidance see CONSORT abstract extension for pilot trials)	*1*
**Introduction**			
Background and objectives	2a	Scientific background and explanation of rationale for future definitive trial, and reasons for randomised pilot trial	2-3
	2b	Specific objectives or research questions for pilot trial	3
**Methods**			
Trial design	3a	Description of pilot trial design (such as parallel, factorial) including allocation ratio	4
	3b	Important changes to methods after pilot trial commencement (such as eligibility criteria), with reasons	n/a
Participants	4a	Eligibility criteria for participants	4
	4b	Settings and locations where the data were collected	4
	4c	How participants were identified and consented	4
Interventions	5	The interventions for each group with sufficient details to allow replication, including how and when they were actually administered	5 and Appendix 2
Outcomes	6a	Completely defined prespecified assessments or measurements to address each pilot trial objective specified in 2b, including how and when they were assessed	5-7
	6b	Any changes to pilot trial assessments or measurements after the pilot trial commenced, with reasons	n/a
	6c	If applicable, prespecified criteria used to judge whether, or how, to proceed with future definitive trial	n/a
Sample size	7a	Rationale for numbers in the pilot trial	4
	7b	When applicable, explanation of any interim analyses and stopping guidelines	n/a
Randomisation:			
Sequence	8a	Method used to generate the random allocation sequence	4

generation	8b	Type of randomisation(s); details of any restriction (such as blocking and block size)	4
Allocation concealment mechanism	9	Mechanism used to implement the random allocation sequence (such as sequentially numbered containers), describing any steps taken to conceal the sequence until interventions were assigned	4
Implementation	10	Who generated the random allocation sequence, who enrolled participants, and who assigned participants to interventions	4
Blinding	11a	If done, who was blinded after assignment to interventions (for example, participants, care providers, those assessing outcomes) and how	4
	11b	If relevant, description of the similarity of interventions	n/a
Statistical methods	12	Methods used to address each pilot trial objective whether qualitative or quantitative	7
**Results**			
Participant flow (a diagram is strongly recommended)	13a	For each group, the numbers of participants who were approached and/or assessed for eligibility, randomly assigned, received intended treatment, and were assessed for each objective	8 and Figure 1
	13b	For each group, losses and exclusions after randomisation, together with reasons	8 and Figure 1
Recruitment	14a	Dates defining the periods of recruitment and follow-up	8
	14b	Why the pilot trial ended or was stopped	8
Baseline data	15	A table showing baseline demographic and clinical characteristics for each group	Table 1
Numbers analysed	16	For each objective, number of participants (denominator) included in each analysis. If relevant, these numbers should be by randomised group	Tables 2 and 3
Outcomes and estimation	17	For each objective, results including expressions of uncertainty (such as 95% confidence interval) for any estimates. If relevant, these results should be by randomised group	Tables 2 and 3
Ancillary analyses	18	Results of any other analyses performed that could be used to inform the future definitive trial	8-10
Harms	19	All important harms or unintended effects in each group (for specific guidance see CONSORT for harms)	n/a
	19a	If relevant, other important unintended consequences	n/a
**Discussion**			
Limitations	20	Pilot trial limitations, addressing sources of potential bias and remaining uncertainty about feasibility	11-13
Generalisability	21	Generalisability (applicability) of pilot trial methods and findings to future definitive trial and other studies	11-13

Interpretation	22	Interpretation consistent with pilot trial objectives and findings, balancing potential benefits and harms, and considering other relevant evidence	11-13
	22a	Implications for progression from pilot to future definitive trial, including any proposed amendments	11-13
**Other information**			
Registration	23	Registration number for pilot trial and name of trial registry	Abstract
Protocol	24	Where the pilot trial protocol can be accessed, if available	4
Funding	25	Sources of funding and other support (such as supply of drugs), role of funders	15
	26	Ethical approval or approval by research review committee, confirmed with reference number	4
