# Supplementary material for: Reducing cardiovascular disease risk among families with familial hypercholesterolaemia by improving diet and physical activity: a randomised controlled feasibility trial
Source: BMJ Open. 2020 Dec 28;10(12):e044200. doi: 10.1136/bmjopen-2020-044200 (PMC7772289; doi:10.1136/bmjopen-2020-044200)
Supplement: Supplementary data [file bmjopen-2020-044200supp002.pdf]

Supplementary file 2. Additional details of intervention content and delivery

Full details of the development of the intervention, and the selection of content and the delivery method, are described elsewhere.[1] This appendix will provide overview of the 1) The behavioural targets of the intervention; 2) The structure and topics covered in the intervention session; 3) Behaviour change techniques (BCTs) included and details of how they were incorporated into the sessions; 4) The BCTs actually delivered in practice to the participants during the initial and follow-up sessions in the study.

1. Behavioural targets of the intervention

All available clinical guidelines for the treatment of children and adults with FH include recommendations for dietary intakes and physical activity. The recommendations are very similar across guidelines, although small differences are apparent. The recommendations selected to be included as targets of the behavioural intervention in this research are summarized below:

1. Total daily fat intake ≤30% of TEI
2. Daily saturated fat intake of ≤10% TEI achieved via replacement with monounsaturated and polyunsaturated fats
3. Daily dietary cholesterol intake ≤300mg
4. Consumption of ≥ 5 portions of fruit and vegetables a day
5. Age appropriate fibre intake: 10-year-olds= 20g/day; 11-16 year-olds = 25g/day and 30g/day for ≥17 years
6. Consumption of 2g of plant sterols or stanols per day
7. Reduce time spent engaged in sedentary behaviors
8. Age appropriate physical activity levels:

- Adults aged 19-64 years: ≥ 150 minutes a week of moderate intensity physical activity or ≥ 75 minutes of vigorous intensity physical activity, or a mixture of the two. Additional activity focussing on improving muscle strength should be undertaken twice a week.
- Children aged 5-18 years: ≥ 60 minutes of moderate-to-vigorous physical activity each day, with 3 of these sessions each week being of vigorous intensity and including activities that strengthen muscle and bone.

Kinnear FJ, *et al. BMJ Open* 2020; 10:e044200. doi: 10.1136/bmjopen-2020-044200

These recommendations reflect the U.K. NICE guidelines,[2] as the developed intervention was to be delivered in an U.K. context, in the first instance. However, three additional recommendations were included for reasons described below:

A) Consumption of 2g/day plant sterol or stanols

While the U.K. NICE guidelines do not consider plant sterols and stanols to be an essential component of dietary treatment,[2] a recommendation for their intake was included in the intervention for the following reasons: the promising evidence to support the addition of plant sterols or stanols to the cholesterol lowering diet in children and adults with FH;[3] the indication for use in individuals with FH from the age of six in EAS and AHA clinical guidelines for the management of high cholesterol;[4-6] recommendation for use in other and more recent clinical guidelines for the management of FH;[7-11] their inclusion in the resources produced by *HEART UK: The cholesterol charity*,[12] which are recommended for use in the recent U.K. guidelines for the management of FH in children.[13]

B) Age appropriate fibre intakes

All the clinical guidelines advocate a Mediterranean dietary pattern and the high fibre content of this dietary pattern may be of particular benefit to individuals with high cholesterol levels.[14-17] Therefore a specific recommendation for fibre intake was included as a specific goal of dietary treatment in this research, using values derived from the guidelines for fiber intake for the general population in the U.K.[18]

C) Reduce time spent engaged in sedentary behaviours

Only the NICE guidelines specify the type or amount of physical activity to be included as part of lifestyle treatment which is based upon the advice for the general population.[2] However, the lifestyle treatment section of the NICE guidelines have not been updated since 2008 and therefore do not include reflect the updated physical activity guidelines for the general population which now include advice to reduce sedentary behaviours.[19] This was therefore added as an additional recommendation to be targeted by the intervention.

D) No specific target for trans fatty acids

While not stated in the U.K. NICE guidelines,[2] other clinical guidelines advocate reducing intake of trans fatty acids (TFA).[8,10-11] This was not selected as a recommendation to be targeted in the intervention in this research for two reasons. Firstly, intakes in the U.K. general population are below the maximum recommended intakes of 2% total energy intake.[20] Secondly, as most TFA are found in processed foods, it is recognised that reducing intakes of TFA requires interventions targeting the use of TFA by the food industry.[21-22] It was therefore not deemed to be appropriate or required for the intervention to target intakes of TFA in the present research. Lastly, in light of the lack of research to support the effectiveness of the current recommendations, it was decided that the intervention would not target additional dietary components that have been investigated to a small extent, such as protein or omega-3 fatty acids.[3]

2. The structure of, and topics covered in, the intervention sessions

The intervention was delivered to families by a dietitian as an initial 1-hour face-to-face session followed by four telephone or email follow-up sessions. The intervention primarily focused upon providing education about the dietary and physical activity recommendations and the importance of adhering to these in addition to pharmacological treatment for the avoidance of long-term health consequences associated with FH. The dietary and physical activity recommendations to be targeted in the intervention were communicated as six targets, which provided structure for the intervention booklets and intervention sessions. The targets were:

1) Reduce how many foods high in saturated fat you eat and replace with foods rich in unsaturated fats
2) Limit how many foods high in dietary cholesterol you eat per week
3) Increase the number of portions of fruits and vegetables you eat each day
4) Increase how many insoluble and soluble fiber-rich foods you eat each day
5) Have one plant sterol or stanol fortified yogurt drink each day
6) Increase how physically active you are and reduce time spent being sedentary

Achievable goals were set for each of the six dietary and physical activity targets, tailored towards each individuals current physical activity levels, dietary intakes and unique personal and family characteristics. These goals were SMART (specific, measurable, acceptable, realistic, time-based) in nature.[23] The dietitians provided

verbal and written (in intervention booklets developed specifically for this trial) instructions on how to achieve these targets such as food swaps, cooking techniques, and recipes. The goals were reviewed at each follow-up session alongside further problem solving and action planning sessions to facilitate continued attainment of goals. Parents and children were encouraged to view each other as sources of emotional and social support and other family members encouraged to adopt the lifestyle changes. Individuals will be encouraged to self-monitor their behaviours through the use of self-reflection diaries and checklists to monitor intake of foods fortified with plant sterols and stanols.

The initial session comprised of eight sections, as shown in Table 1 alongside the intended aims for participants. Each follow-up session comprised of four sections: *introduction and overview; review of goals; barriers and solutions; further support and follow up*.

3. **Behaviour change techniques (BCTs) included and details of how they were incorporated into the sessions**

Table 2 displays the BCTs to be included in the initial session and Table 3 displays the BCTs to be included in the follow-up sessions. These 'BCT checklists' were provided to dietitians to provide them with instructions on how to incorporate the BCTs into the sessions and to monitor fidelity. Descriptions of each BCT are provided in full in The BCT Taxonomy (V1).[24] Dietitians were instructed to record any adaptations they made to the intended BCTs- including if they delivered other additional BCTs.

Table 1. The eight sections of the initial intervention session and participant aims

Section	Aim(s) for participants
**1. Introduction<br>&<br>2. Scientific rationale**	• To understand the importance of diet and physical activity in the management of FH and for their overall health.<br>• To be aware of the importance placed on diet and physical activity by national and international guidelines for FH and the current recommendations in England that all FH patients should receive individualised advice about diet & physical activity<br>• To understand that the earlier treatment for FH starts, the more effective it is and this is why it is important to optimise nutrition and physical activity from a young age
**3. Education about dietary guidelines**	• To know what a healthy balanced diet looks like, including the food groups and the proportion each one should make to diet<br>• To know what the five diet targets of the intervention are<br>• To understand why each target is important for their health and for the management of their FH<br>• To understand what foods to include/exclude and/or increase/decrease consumption of to achieve targets
**4. Education about physical activity guidelines**	• To know what the physical activity and sedentary behaviour recommendations are for their age<br>• To know why these recommendations are important for their health and for management of their FH<br>• To know what the different levels of physical activity are (low, moderate and high) and what types of physical activity fall into which group<br>• To understand how they can incorporate more physical activity into their lifestyle to help increase levels to recommended amounts, or more in addition to reducing sedentary behaviours.
**5. Goal setting**	• To agree with dietitian upon SMART goals for each of the targets. These will be changes to their lifestyle that they agree to make over the following 12 weeks to achieve nutritional intakes and physical activity levels closer to the guidelines
**6. Barriers and solutions**	• To identify potential barriers that may prevent them from meeting the goals set<br>• To identify, through discussion with dietitian and other family member, solutions to these barriers
**7. Follow up arrangements<br>&<br>8. Wrap-up**	• To understand what will happen over the following 12 weeks<br>• To understand the purpose of the weekly reflection diaries and know how to fill them out<br>• To know what will be discussed during the follow-up sessions<br>• An opportunity to ask any unanswered questions<br>• To receive summary of session discussion & receive encouragement and motivation from dietitian

Table 2. BCT checklist for initial session of intervention to be completed by dietitians

Behaviour change technique	How to be used within intervention	Included?
Action planning	Individual will be prompted to develop specific planning of how they will achieve each goal set i.e. *if increasing fibre intake then the food swap or additional food to be included in diet will be specified, along with what meal or snack they will include it in and how many times per day or week.*	
Behaviour substitution	Individuals encouraged to set goals that involve swapping something they currently do everyday with something that will help them achieve the guidelines to help establish new healthy habits i.e. *walking to and from work instead of driving* or *always having a piece of fruit when they make their morning cup of tea instead of a biscuit*.	
Behavioural practice/rehearsal	Prompt individual to practice cooking during intervention and adults to encourage child to help them.	
Biofeedback	Individual to be provided with weight, body fat % and blood pressure before starting the intervention to prompt adoption of guidelines- either to improve these figures or maintain them.	
Comparative imaging of future outcomes	Dietitian to prompt individual to think about what the possible health outcomes would be if they choose to follow guidelines compared to if they chose not to- with emphasis on what this would mean for their children or parents.	
Credible sources	• Intervention delivered by a dietitian who will explain the training they have undertaken to gain that title- to help individual recognise that their advice is credible. All individuals will also be informed that their doctor is aware and supportive of them receiving the intervention as they view it as being part of their clinical care.<br>• Individual to be receive verbal and written advice about the guidelines which will include information about where the guidelines have come from- national committees across several countries who have reviewed all the available scientific evidence and come to same conclusion about the guidelines that individuals with FH should be following.	
Demonstration of the behaviour	Individual to be signposted to resources which can aid with demonstration of the behaviour i.e. *step by step recipe videos or pictorial guides and parents encouraged to demonstrate cooking skills to child.*	
Feedback on outcomes of behaviour	Individual to receive feedback about how their dietary intakes and physical activity levels compare to the guidelines at the start and end of the intervention as evidence of their capability of adhering to the guidelines to promote maintenance of behaviours after intervention ended.<br>Note- the follow-up information will be delivered at research contact 3. Provide initial information in initial session.	
Framing/re-framing	• Guidelines communicated to individual as being specifically for individuals with FH as opposed to general healthy lifestyle guidelines provided to all individuals. The inclusion of two specific dietary guidelines for individuals with FH such as eating foods fortified with plant stanols/sterols and reducing dietary cholesterol intakes will help individuals to buy into the idea that following the guidelines is part of their identity of having FH. The benefits of following the guidelines in the management of their FH will be emphasised, in addition to general overall health benefits.<br>• Dietary guidelines to be communicated as a healthy lifestyle rather than a restrictive diet, with all foods permitted. Emphasis will be put on foods to add into the diet (fruits, vegetables, fibre rich foods, plant sterols/stanols) to help individual view dietary choices are positive and enjoyable.<br>• Physical activity to be communicated positively, with emphasis placed upon finding activities that the individual enjoys doing, either alone or with friends or family, rather than it being a chore they have to try and fit into their day without enjoying it.<br>• Individual prompted to view the guidelines as behaviours that can help reduce their risk of developing symptoms as their family members have, or reduce the likelihood of experiencing further symptoms. They are something the individual can do to help take control of their health.	
Goal setting (behavioural)	• Individual prompted to set their own goals which they feel are achievable-which take into consideration their food preferences, fitness levels, readiness to change and lifestyles. The goals will be SMART. Parents will be encouraged to help child with setting their goals.	
Graded tasks	• Child to be prompted to help with food preparation, starting with simple task such as preparing vegetables, progressing to more complex tasks until able to prepare full meal.<br>• Adults to be prompted to start cooking meals- starting with simple recipes and progressing to more complex ones.	
Identification of self as a role model	• Parents encouraged to view themselves as role models for their children and make their behaviours part of everyday life for their children to help them foster adoption of healthy habits from a young age. Dietitian to communicate the benefits engaging in the behaviours could bring to these significant others.<br>• Children also encouraged to view themselves as role models for their parents, other siblings and friends.	
Information about antecedents	Individual prompted to consider specific contexts in which they find it hard to adhere to dietary guidelines and identify triggers for this behaviour. The findings will be addressed in action planning and problem-solving session of the intervention.	

Kinnear FJ, *et al. BMJ Open* 2020; 10:e044200. doi: 10.1136/bmjopen-2020-044200

Information about emotional consequences	• Dietitian to discuss with individual the potential improvement in their mood that they could experience if they choose to engage in dietary behaviours.<br>• Dietitian to emphasis to individual that if they find foods that they enjoy that fit the guidelines, they will also experience the same enjoyment of these foods.	
Information about health consequences	• The intervention starts with explanation from dietitian about the importance of lifestyle guidelines in the management of FH. It will be explained that despite use of medication, many individuals with FH may still be at higher risk of cardiovascular disease and adherence to lifestyle guidelines can help reduce this risk. The benefits to their overall health will also be communicated.<br>• Individuals to be informed about the 'silent' nature of cholesterol and the importance of keeping cholesterol low for your whole life, before any symptoms occur.	
Instruction on how to perform behaviour	• Individuals will receive detailed instructions about food swaps and cooking methods. The advice given will provide instructions to individual about how they can achieve the desired behaviours in their current *context i.e. cheaper options for suggested food swaps, suggested food swaps one they can obtain in the current place they shop, easy and quick options for meals if individual has limited time to cook and suggestions of physical activity they can fit into their current routines such as walking instead of bus/car.*<br>• The dietary intake data will be analysed ahead of intervention to establish any existing eating habits that are indicative of misinformation i.e. *cooking with coconut oil.*	
Problem solving	During intervention 'barriers and solutions' section, dietitian will encourage individual to think of situations in which they feel they will struggle to engage in the desired behaviours (*i.e. social situations*) and think of solutions to overcome these.	
Prompts and cues	• Individual advised to leave intervention booklet in a place they regularly eat to prompt them to engage in the dietary goals.<br>• Set reminders on phones/fitness monitors to move regularly.	
Restructuring the physical environment	• Advise individual to keep food choices they enjoy eating, that are in line with the guidelines, in the house/at work/in car to encourage consumption of these<br>• Individuals encouraged to socialise with friends and family in places that facilitate engagement of desired behaviours i.*e. meet in the park* or *choose restaurants in which there are suitable options for them.*	
Restructuring the social environment	The intervention is delivered at a family-based level with parent and child making dietary and physical activity choices together. Any other family members will also be encouraged to make the changes as well to facilitate a home environment that encourages adherence to the dietary and physical activity guidelines.	
Social support (emotional)	• Dietitian to provide emotional support during intervention- discussing with them what else is going on in their life and how this is influencing their ability to adhere to guidelines.<br>• Individual also encouraged to seek support from the family member(s) they are taking part in the intervention with- to view it as a 'team effort' and provide support and encouragement to each other.<br>• Dietitian to encourage parent and child to provide support to each other throughout the intervention and encourage each other to engage in the guidelines.<br>• Individuals also encouraged to seek the emotional support of friends and family.	
Social support (practical)	• Dietitian to provide practical support during the session- helping individual to identify methods to help encourage the behaviour *i.e. online recipes or suggestions on how they could obtain practical support from friends* or *having their parent prepare help make their lunch.*<br>• The dietitian will encourage the whole family (both with and without FH) to engage in the dietary and physical activity guidelines to provide support to the individuals.<br>• Individuals encouraged to seek support from family members (including those not taking part in intervention) and friends i*.e. arranging to go to the gym with a friend or having partner/sibling help with food shopping or meal preparation.*	
Verbal persuasion about capabilities	Dietitian to encourage individual and tell them that they are capable of changing their behaviours.	
Self-monitoring of behaviour (prompt)	Individuals asked to complete weekly reflection diaries in which they record whether they have been able to meet the goals set for lifestyle behaviours.<br>They will also be asked to keep checklists of plant stanol/sterol drink consumption.	

Kinnear FJ, *et al. BMJ Open* 2020; 10:e044200. doi: 10.1136/bmjopen-2020-044200

Table 3. **BCT checklist for intervention follow-up sessions completed by dietitians**

Behaviour change technique	Instructions for use within intervention	Included?
Action planning	• Individual will be prompted to develop specific planning of how they will continue to, or start to, achieve each goal set at previous and current session i.e. if increasing fibre intake then the food swap or additional food to be included in diet will be specified, along with what meal or snack they will include it in and how many times per day or week.<br>• Follow up sessions are opportunity to review action plans set a previous session and identify what has worked and what needs more planning.	
Problem solving	• During 'barriers and solutions' section, dietitian will review with individual the barriers and solutions they discussed at previous session. Together they will identify what they will do next if barrier still preventing them from achieving goal.<br>• Dietitian will encourage individual to think of other situations in which they feel they will struggle to engage in the desired behaviours between now and next follow up session and think of solutions to overcome these.	
Social support (emotional)	• Dietitian to provide emotional support during follow-up sessions- discussing with them what else is going on in their life and how this is influencing their ability to adhere to guidelines.<br>• Individuals encouraged to seek the emotional support of friends and family<br>• Individual encouraged to seek support from the family member(s) they are taking part in the intervention with- to view it as a 'team effort' and provide support and encouragement to each other to engage in the guidelines	
Social support (practical)	• Dietitian provide practical support during follow-up sessions- helping individual to identify methods to help encourage the behaviour i.e. links to online resources or suggestions on how they could obtain practical support from friends or family i.e. having their parent prepare help make their lunch<br>• Individuals encouraged to seek support from family members (including those not taking part in intervention) and friends i.e. arranging to go to the gym with a friend or having partner/sibling help with food shopping or meal preparation.	
Verbal persuasion about capabilities	Dietitian to encourage individual and tell them that they are capable of changing their behaviours.	
Focus on past success	Dietitian to focus on any successful changes individuals have made and use these to provide encouragement and motivation to individual to carry on and make more changes.	
Review behavioural goal(s)	At each follow-up session, dietitian and individual will review the goals set at previous session or follow-up. Together they will agree to either: keep goal the same, modify the goal or create new goal. These decisions will be based upon individuals levels of achievement and willingness to change.	
Self-monitoring of behaviour (prompt to)	Individuals asked to complete weekly reflection diaries in which they record whether they have been able to meet the goals set for lifestyle behaviours which will be discussed during follow-ups. They will also be asked to keep checklists of plant stanol drink consumption which will be reviewed at each follow up session.	
Self-monitoring of behaviour (as reported by individuals)	Individuals self-reported completion of the weekly self-reflection diaries and plant stanol checklist.	

Kinnear FJ, *et al. BMJ Open* 2020; 10:e044200. doi: 10.1136/bmjopen-2020-044200

4. The BCTs actually delivered in practice to the participants during the initial and follow-up sessions.

The BCTs intended to have been delivered in the initial and follow up sessions are indicated with a tick in Table 4 alongside the percentage of families who received each one in practice. Further details of how each BCT was to be delivered in the intervention are displayed in Table 2 and Table 3. Overall, the fidelity was high with the majority of BCTs delivered as intended across all sessions and sites. Four BCTs (*demonstration of the behaviour*; *graded tasks*, *problem solving* and *self-monitoring of behaviour prompt)* were not delivered to all families. In all instances, this was reported by the dietitians to be due to it not being applicable to the individual family. For instance, the dietitian reported it was not appropriate to prompt families, who had previously stated they were not, or did not want to, use the self-reflection resources at previous follow-up sessions. The families stated they did not have time or did not feel it was necessary to help them achieve their goals. Two further BCTs *biofeedback* and *feedback on outcomes of behaviour* were not delivered to families (n=2) at the London study site as the dietitian did not have access to the dietary intake or anthropometric data ahead of the initial session.

In response to the needs of each individual family, the dietitians reported the occasional use of additional BCTs during the follow-up sessions, as indicated by the plus sign in Table 4. The dietitians reported using these BCTs to help families overcome barriers they were facing in achieving their goals. For instance, *instruction on how to perform the behaviour* was delivered to 20% of families during follow-up one who had requested recipes or cooking advice.

Kinnear FJ, *et al. BMJ Open* 2020; 10:e044200. doi: 10.1136/bmjopen-2020-044200

Table 4. The BCTs intended to be included in the initial and follow-up sessions (✔), the additional BCTs delivered (+) and the percentage of families who received each BCT in each session (%)

Behaviour change technique (BCT)* †	Initial session (n=10)	Follow-up 1 (n=10)	Follow-up 2 (n=10)	Follow-up 3 (n=10)	Follow-up 4 (n=10)
**Action planning**	✔ (100%)	✔ (100%)	✔ (100%)	✔ (100%)	✔ (100%)
**Behaviour substitution**	✔ (100%)	+ (20%)	+ (10%)	+ (5%)	
**Behavioural practice/rehearsal**	✔ (100%)				
**Biofeedback**	✔ (80%)				
**Comparative imaging of future outcomes**	✔ (100%)				
**Credible sources**	✔ (100%)				
**Demonstration of the behaviour**	✔ (0%)	+ (5%)			
**Feedback on outcomes of behaviour**	✔ (90%)				
**Framing/re-framing**	✔ (100%)	+ (25%)	+ (25%)	+ (5%)	
**Goal setting (behavioural)**	✔ (100%)				
**Graded tasks**	✔ (30%)				
**Identification of self as a role model**	✔ (100%)				
**Information about antecedents**	✔ (100%)				
**Information about emotional consequences**	✔ (100%)				
**Information about health consequences**	✔ (100%)				
**Instruction on how to perform behaviour**	✔ (100%)	+ (20%)	+ (10%)	+ (11%)	
**Problem solving**	✔ (100%)	✔ (100%)	✔ (100%)	✔ (84%)	✔ (100%)
**Prompts and cues**	✔ (100%)		+ (5%)	+ (5%)	
**Restructuring the physical environment**	✔ (100%)	+ (10%)			
**Restructuring the social environment**	✔ (100%)				
**Social support (emotional)**	✔ (100%)	✔ (100%)	✔ (100%)	✔ (100%)	✔ (100%)
**Social support (practical)**	✔ (100%)	✔ (100%)	✔ (100%)	✔ (100%)	✔ (100%)
**Verbal persuasion about capabilities**	✔ (100%)	✔ (100%)	✔ (100%)	✔ (100%)	✔ (100%)
**Focus on past success**		✔ (100%)	✔ (100%)	✔ (100%)	✔ (100%)
**Review behavioural goal(s)**		✔ (100%)	✔ (100%)	✔ (100%)	✔ (100%)
**Self-monitoring of behaviour (prompt to)**	✔ (90%)	✔ (100%)	✔ (100%)	✔ (68%)	✔ (50%)

*Details of how each BCT was delivered in the intervention are available in Table 2 and Table 3.
† Further details of the BCTs are available on the BCT taxonomy (BCTTv1)[24]

References

1.Kinnear FJ, Wainwright E, Bourne JE, Lithander FE, Hamilton-Shield J, Searle A. The development of a theory informed behaviour change intervention to improve adherence to dietary and physical activity treatment guidelines in individuals with familial hypercholesterolaemia (FH). BMC health services research. 2020;20(1):27.
2.NICE clinical guideline 71: Familial hypercholesterolemia: identification and management. [Internet]. 2008 [cited 19/01/2018]. Available from: http://nice.org.uk/guidance/cg71.
3.Malhotra A, Shafiq N, Arora A, Singh M, Kumar R, Malhotra S. Dietary interventions (plant sterols, stanols, omega-3 fatty acids, soy protein and dietary fibers) for familial hypercholesterolaemia. Cochrane Database Syst Rev. 2014(6):CD001918.
4.Gylling H, Plat J, Turley S, Ginsberg HN, Ellegard L, Jessup W, et al. Plant sterols and plant stanols in the management of dyslipidaemia and prevention of cardiovascular disease. Atherosclerosis. 2014;232(2):346-60.
5.Mach F, Baigent C, Catapano AL, Koskinas KC, Casula M, Badimon L, et al. 2019 ESC/EAS Guidelines for the management of dyslipidaemias: lipid modification to reduce cardiovascular risk: The Task Force for the management of dyslipidaemias of the European Society of Cardiology (ESC) and European Atherosclerosis Society (EAS). European heart journal. 2019;41(1):111-88.
6.Grundy Scott M, Stone Neil J, Bailey Alison L, Beam C, Birtcher Kim K, Blumenthal Roger S, et al. 2018 AHA/ACC/AACVPR/AAPA/ABC/ACPM/ADA/AGS/APhA/ASPC/NLA/PCNA Guideline on the Management of Blood Cholesterol. Circulation.0(0):CIR.0000000000000625.
7.Goldberg AC, Hopkins PN, Toth PP, Ballantyne CM, Rader DJ, Robinson JG, et al. Familial hypercholesterolemia: screening, diagnosis and management of pediatric and adult patients: clinical guidance from the National Lipid Association Expert Panel on Familial Hypercholesterolemia. J Clin Lipidol. 2011;5(3 Suppl):S1-8.
8.Nordestgaard BG, Chapman MJ, Humphries SE, Ginsberg HN, Masana L, Descamps OS, et al. Familial hypercholesterolaemia is underdiagnosed and undertreated in the general population: guidance for clinicians to prevent coronary heart disease : Consensus Statement of the European Atherosclerosis Society. European heart journal. 2013;34(45):3478-90.
9.Wiegman A, Gidding SS, Watts GF, Chapman MJ, Ginsberg HN, Cuchel M, et al. Familial hypercholesterolaemia in children and adolescents: gaining decades of life by optimizing detection and treatment. European heart journal. 2015;36(36):2425-37.
10.Watts GF, Gidding S, Wierzbicki AS, Toth PP, Alonso R, Brown WV, et al. Integrated guidance on the care of familial hypercholesterolaemia from the International FH Foundation. International journal of cardiology. 2014;171(3):309-25.
11.Gidding SS, Champagne MA, de Ferranti SD, Defesche J, Ito MK, Knowles JW, et al. The Agenda for Familial Hypercholesterolemia: A Scientific Statement From the American Heart Association. Circulation. 2015;132(22):2167-92.
12.HEART UK: The Cholesterol Charity. Familial hypercholesterolaemia: An education booklet for people with familial hypercholesterolaemia (FH). London HEART UK: The cholesterol charity; 2019.
13.Ramaswami U, Humphries SE, Priestley-Barnham L, Green P, Wald DS, Capps N, et al. Current management of children and young people with heterozygous familial hypercholesterolaemia - HEART UK statement of care. Atherosclerosis. 2019;290:1-8.

14.McRae MP. Dietary Fiber Is Beneficial for the Prevention of Cardiovascular Disease: An Umbrella Review of Meta-analyses. Journal of Chiropractic Medicine. 2017;16(4):289-99.
15.Brown L, Rosner B, Willett WW, Sacks FM. Cholesterol-lowering effects of dietary fiber: a meta-analysis. The American journal of clinical nutrition. 1999;69(1):30-42.
16.Whitehead A, Beck EJ, Tosh S, Wolever TM. Cholesterol-lowering effects of oat β-glucan: a meta-analysis of randomized controlled trials. The American journal of clinical nutrition. 2014;100(6):1413-21.
17.Ho HV, Sievenpiper JL, Zurbau A, Blanco Mejia S, Jovanovski E, Au-Yeung F, et al. The effect of oat β-glucan on LDL-cholesterol, non-HDL-cholesterol and apoB for CVD risk reduction: a systematic review and meta-analysis of randomised-controlled trials. Br J Nutr. 2016;116(8):1369-82.
18.Nutrition SACo. Carbohydrates and Health. London: The Stationery Office (TSO); 2015.
19.Department of Health and Social Care. UK Chief Medical Ofiicers' Physical Activity Guidelines London: UK Government Department of Health and Social Care; 2019.
20.Public Health England. National Diet and Nutrition Survey Results from Years 7 and 8 (combined) of the Rolling Programme (2014/2015 to 2015/2016). London: Public Health England and Food Standards Agency 2018.
21.Hyseni L, Bromley H, Kypridemos C, O'Flaherty M, Lloyd-Williams F, Guzman-Castillo M, et al. Systematic review of dietary trans-fat reduction interventions. Bulletin of the World Health Organization. 2017;95(12):821-30G.
22.Hendry VL, Almíron-Roig E, Monsivais P, Jebb SA, Neelon SE, Griffin SJ, et al. Impact of regulatory interventions to reduce intake of artificial trans-fatty acids: a systematic review. American journal of public health. 2015;105(3):e32-42.
23.Ogbeiwi O. Why written objectives need to be really SMART. British Journal of Healthcare Management. 2017;23(7):324-36.
24.Michie S, Richardson M, Johnston M, Abraham C, Francis J, Hardeman W, et al. The behavior change technique taxonomy (v1) of 93 hierarchically clustered techniques: building an international consensus for the reporting of behavior change interventions. Ann Behav Med. 2013;46(1):81-95.
