# Supplementary material for: Reducing cardiovascular disease risk among families with familial hypercholesterolaemia by improving diet and physical activity: a randomised controlled feasibility trial
Source: BMJ Open. 2020 Dec 28;10(12):e044200. doi: 10.1136/bmjopen-2020-044200 (PMC7772289; doi:10.1136/bmjopen-2020-044200)
Supplement: Supplementary data [file bmjopen-2020-044200supp003.pdf]

Supplementary file 3. Additional details of outcome measures used in study

Behavioural outcomes (study objectives 5 & 6)

Behavioural outcomes were collected in the week preceding research contact two (baseline) and research contact three (endpoint).

1. Dietary intakes: participants recorded their dietary intake on four non-consecutive days (including one weekend day) using Intake24, a validated online 24-hour recall tool.[1,2] Data were downloaded from Intake24 as an Excel file before being cleaned and analysed, in line with Intake24 data processing guidelines. After data quality checks, data not meeting the validity criteria (completion rate <2 minutes; daily energy intake <2000KJ or >16000KJ) were excluded and only participants with two weekdays and one weekend day of valid intake were included in the analysis. Using pivot tables, daily intakes of macronutrients were calculated. Intakes of fruits and vegetables were presented as mean portions consumed per day by dividing the sum intake of fruit and vegetables by 80, per participant and per day. The value of 80 was chosen as a single portion of fruit and vegetables, in U.K. national guidelines, is considered to be 80g. Dried fruit, fruit juices and pulses were included but a portion was considered to be 30g, 150ml and 80g respectively, and fruit juices and pulses were capped at one portion per day. Fruits and vegetables in composite dishes were not included as there is not yet a validated method to calculate this using the Intake24 database. In line with the specific dietary goals of the intervention described in appendix 2, mean daily intakes of fat, saturated fat, monounsaturated fats, polyunsaturated fats, cholesterol, fiber, fruit and vegetable portions, and plant sterol or stanol fortified foods were chosen as outcome measures.

2. Physical activity levels: average daily minutes of free-living moderate and vigorous physical activity (MVPA) was measured by asking participants to wear an ActiGraph GT3X+ accelerometer monitor (ActiGraph, Pensacola, USA) during waking hours for seven consecutive days. Participants also completed logbooks to capture waking/sleeping times and any activities during which the monitor was taken off. Raw data were downloaded and analysed, in 15 and 60-second epochs for children and adults respectively, using the manufacturer's software (ActiLife software v6.13.4; ActiGraph, Pansacola, FL, USA). Logbook data and an algorithm, which detected non-wear as periods of 60 minutes or more of consecutive zeros with no allowance

Kinnear FJ, *et al. BMJ Open* 2020; 10:e044200. doi: 10.1136/bmjopen-2020-044200

for interruptions, was used to identify valid weartime periods. A second researcher (AET) analysed the data from 10 participants (five adults and five children) to ensure consistency and validity of the decisions made by FJK. To determine MVPA, the cut-points developed by Freedson[3] and Evenson[4] were used for adults and children, respectively. At least four valid days (defined as ≥ 8 hours weartime), including at least one weekend day, were required for analysis.

3. Sedentary time: participants wore an activPAL3 accelerometer (PAL Technologies Ltd., Glasgow, UK) for seven consecutive days to estimate the average daily time spent sedentary (sitting or lying).[5] Accelerometers were made waterproof using a nitrile sleeve and were attached to the front of the thigh using a waterproof hypoallergenic medical dressing (Hypafix), enabling participants to wear the monitor continuously for 24 hours/day, removing only for swimming. Data was downloaded using the manufacturer's software (PAL software v7.2.38; PAL Technologies Ltd., Glasgow, UK) and was cleaned and processed in ProcessingPAL, a Java application which utilises a validated algorithm to isolate waking weartime.[6] Logbooks were used to aid identification of the non-wear periods and sleep time, which were removed from analysis. A second researcher (AET) analysed the data from 10 participants (five adults and five children) to ensure consistency and validity of the decisions made by FJK. At least four valid days (defined as ≥10 h of worn waking hours, <95% of time spent in any one position and ≥500 steps), including one weekend day was required for analysis.

Table 1. Data processing and analysis decisions for data collected using Actigraph accelerometers

Factor to consider	Description	Approach taken in this study *Adults*	*Children*
**EPOC length**	EPOC length is the time period over which the ActiGraph counts are totalled and can range between 1 and 60 seconds.	• 60 second epochs	• 15 second epochs
**Cut-points for classification of activity intensity**	To interpret the counts per epoch, thresholds are applied to define different intensities of PA known as cut-points. There are several sets of cut-points available.	• Freedson et al[3]	• Evenson et al[4]
**Classification of non-wear time**	Periods of time that participant is not wearing Actigraph should be detected and deleted from the data to avoid misclassifying non-wear time as sedentary time. There are a variety of available algorithms to detect non-wear time, which differ in the criteria they use.	• An algorithm which detects non-wear as periods of 60 minutes or more of consecutive zeros with no allowance for interruptions<br>• Participants are asked to complete logbooks to capture non-wear occasions and reasons, to cross check results from algorithm<br>• Self-reported PA during non-wear times (e.g. swimming or contact sports) were not included in analysis	
**Valid day & minimum number of days criteria**	The number of hours of weartime required to define a valid day, and the number of these valid days required for data to be included in the analysis.	• A valid day is defined as ≥ 8 hours (480 minutes)<br>• Four valid days (including one weekend day) were required for inclusion in analysis	
**Uni- or tri-axial counts**	The ActiGraph detects activity counts across three axes (vertical, mediolateral and anteroposterior). Researchers can choose to use the vector magnitude (VM) score, which includes activity counts detected across all axes, or use activity counts detected across vertical axis only.	• Activity counts detected across vertical axis were analysed to match the approach used in the studies that developed the MVPA cut-points	

Clinical outcomes (Study objectives 5 and 6)

Clinical outcomes were collected from participants at research contact two (baseline) and three (endpoint) by members of the research team.

1. Anthropometry and body composition: Height was measured using a stadiometer and weight, body fat percentage and fat free mass using bioelectrical impedance scales (Tanita™ MC-780MA). At sites where this equipment was unavailable, weight was measured using medical scales. These outcomes were used to calculate the body mass index (BMI) for adult participants. The LMS method was used to express the BMIs of child participants as BMI Z-scores and percentiles.[7] This was carried out using the lmsGrowth excel add-in[8] and the British 1990 reference population.[9] The 91st and 98th BMI percentiles were applied to define overweight and obesity as recommended for clinical interventions.[10]

2. Resting arterial blood pressure: The mean of two sphygmomanometer readings (three if 1st and 2nd differed by more than 10mmHg) was calculated to provide systolic and diastolic blood pressure outcomes. Participants were seated during the measurements, which were taken after a period of at least five minute of rest to allow values to return to resting levels.

3. HRQoL: Children completed an age appropriate Pediatric Quality of Life Inventory ™ (PedsQL™) Version 4.0 and adults completed an EuroQol Group EQ-5D-3L health questionnaire.[11,12] For children, the completed inventories were scored using the recommended scoring system[13] to produce a score for HRQoL and a breakdown of the component physical and psychosocial (emotional, social and school) functioning scores. For adults, the recommended scoring system was used[12] to produce a five-digit health state profile representative of five dimensions of health which was converted into a single index value using a validated value set created for use in the United Kingdom.[14] The visual analogue scale data was presented as a single value between 1 and 100.

4. Blood lipid profile: 25ml fasted blood samples were collected and a proportion was analysed immediately in the local NHS laboratory. Analysis was done by routine homogeneous enzymatic methods using Cobas reagents (Cholesterol Gen 2;

Triglyceride Gen 1; HDL Gen 3 or 4) on Roche/Hitachi c701/2 analysers. LDL-C concentration was then calculated using the Friedewald equation.[15] The remaining sample was processed and stored at -80°C as plasma or serum. Subsequent batch metabolomics analysis to determine 201 metabolomics is planned, but not presented in this manuscript due to laboratory closure during the COVID-19 outbreak.

References

1.Simpson E, Bradley J, Poliakov I, Jackson D, Olivier P, Adamson AJ, et al. Iterative Development of an Online Dietary Recall Tool: INTAKE24. Nutrients. 2017;9(2):118.
2.Bradley J, Simpson E, Poliakov I, Matthews J, Olivier P, Adamson A, et al. Comparison of INTAKE24 (an Online 24-h Dietary Recall Tool) with Interviewer-Led 24-h Recall in 11–24 Year-Old. Nutrients. 2016;8(6):358.
3.Freedson PS, Melanson E, Sirard J. Calibration of the Computer Science and Applications, Inc. accelerometer. Medicine & Science in Sports & Exercise. 1998;30(5):777-81.
4.Evenson KR, Catellier DJ, Gill K, Ondrak KS, McMurray RG. Calibration of two objective measures of physical activity for children. J Sports Sci. 2008;26(14):1557-65.
5.Atkin AJ, Gorely T, Clemes SA, Yates T, Edwardson C, Brage S, et al. Methods of Measurement in epidemiology: Sedentary Behaviour. International Journal of Epidemiology. 2012;41(5):1460-71.
6.Winkler EA, Bodicoat DH, Healy GN, Bakrania K, Yates T, Owen N, et al. Identifying adults' valid waking wear time by automated estimation in activPAL data collected with a 24 h wear protocol. Physiological measurement. 2016;37(10):1653-68.
7.Green PJ, Cole TJ. Smoothing reference centile curves: the LMS method and penalized likelihood. Statistics in Medicine. 1992;11:1305-19.
8.Pan HaC, T User’s guide to lmsGrowth. London, U.K. : Medical Research Council; 2012.
9.Cole TJ, Freeman JV, Preece MA. Body mass index reference curves for the UK, 1990. Archives of Disease in Childhood. 1995;73(1):25.
10.Scientific Advisory Committee on Nutrition (SACN). SACN/RCPCH defining child underweight, overweight and obesity in the UK In: England PH, editor. London: Public Health England; 2012.
11.Rabin R, de Charro F. EQ-5D: a measure of health status from the EuroQol Group. Ann Med. 2001;33(5):337-43.
12.EuroQoL Research Foundation. EQ-5D-3L User Guide Basic information on how to use the EQ-5D-3L instrument. Version 6.0. . 2018.
13.Varni JW. Scaling and scoring of the Pediatric Quality of Life Inventory ™ PedsQL ™. 2017 [Available from: http://www.pedsql.org/PedsQL-Scoring.pdf.
14.Dolan P. Modeling valuations for EuroQol health states. Medical care. 1997;35(11):1095-108.
15.Friedewald WT, Levy RI, Fredrickson DS. Estimation of the concentration of low-density lipoprotein cholesterol in plasma, without use of the preparative ultracentrifuge. Clin Chem. 1972;18(6):499-502
