# Supplementary material for: Reducing cardiovascular disease risk among families with familial hypercholesterolaemia by improving diet and physical activity: a randomised controlled feasibility trial
Source: BMJ Open. 2020 Dec 28;10(12):e044200. doi: 10.1136/bmjopen-2020-044200 (PMC7772289; doi:10.1136/bmjopen-2020-044200)
Supplement: Author's manuscript [file bmjopen-2020-044200.draft_revisions.pdf]

BMJ Open is committed to open peer review. As part of this commitment we make the peer review history of every article we publish publicly available.

When an article is published we post the peer reviewers' comments and the authors' responses online. We also post the versions of the paper that were used during peer review. These are the versions that the peer review comments apply to.

The versions of the paper that follow are the versions that were submitted during the peer review process. They are not the versions of record or the final published versions. They should not be cited or distributed as the published version of this manuscript.

BMJ Open is an open access journal and the full, final, typeset and author-corrected version of record of the manuscript is available on our site with no access controls, subscription charges or pay-per-view fees (http://bmjopen.bmj.com).

If you have any questions on BMJ Open's open peer review process please email info.bmjopen@bmj.com

BMJ Open

# Reducing cardiovascular disease risk amongst families with familial hypercholesterolaemia by improving diet and physical activity: A randomised controlled feasibility trial



| | |
|---|---|
| Journal: | *BMJ Open* |
| Manuscript ID | bmjopen-2020-044200 |
| Article Type: | Original research |
| Date Submitted by the Author: | 31-Aug-2020 |




| | |
|---|---|
| Complete List of Authors: | Kinnear, Fiona; University Hospitals Bristol and Weston NHS Foundation Trust, National Institute for Health Research (NIHR) Bristol Biomedical Research Centre (BRC); University of Bristol<br>Lithander, Fiona; University Hospitals Bristol and Weston NHS Foundation Trust, National Institute for Health Research (NIHR) Bristol Biomedical Research Centre (BRC); University of Bristol, Bristol Medical School<br>Searle, Aidan; University Hospitals Bristol and Weston NHS Foundation Trust, National Institute for Health Research (NIHR) Bristol Biomedical Research Centre (BRC); University of Bristol<br>Bayly, Graham; University Hospitals Bristol and Weston NHS Foundation Trust, Department of Clinical Biochemistry<br>Wei, Christina; St George's University Hospitals NHS Foundation Trust<br>Stensel, David; Loughborough University, National Centre for Sport and Exercise Medicine, School of Sport, Exercise and Health Sciences; University Hospitals of Leicester NHS Trust, National Institute for Health Research (NIHR) Leicester Biomedical Research Centre (BRC)<br>Thackray, Alice; Loughborough University, National Centre for Sport and Exercise Medicine, School of Sport, Exercise and Health Sciences; University Hospitals of Leicester NHS Trust, National Institute for Health Research (NIHR) Leicester Biomedical Research Centre (BRC)<br>Hunt, Linda; University Hospitals Bristol and Weston NHS Foundation Trust, National Institute for Health Research (NIHR) Bristol Biomedical Research Centre (BRC); University of Bristol<br>Shield, Julian; University Hospitals Bristol and Weston NHS Foundation Trust, National Institute for Health Research (NIHR) Bristol Biomedical Research Centre (BRC); University of Bristol |




| | |
|---|---|
| Keywords: | CARDIOLOGY, Lipid disorders < DIABETES & ENDOCRINOLOGY, NUTRITION & DIETETICS, Paediatric endocrinology < DIABETES & ENDOCRINOLOGY, PREVENTIVE MEDICINE, QUALITATIVE RESEARCH |
| | |



SCHOLARONE™
Manuscripts

*I, the Submitting Author has the right to grant and does grant on behalf of all authors of the Work (as defined in the below author licence), an exclusive licence and/or a non-exclusive licence for contributions from authors who are: i) UK Crown employees; ii) where BMJ has agreed a CC-BY licence shall apply, and/or iii) in accordance with the terms applicable for US Federal Government officers or employees acting as part of their official duties; on a worldwide, perpetual, irrevocable, royalty-free basis to BMJ Publishing Group Ltd ("BMJ") its licensees and where the relevant Journal is co-owned by BMJ to the co-owners of the Journal, to publish the Work in this journal and any other BMJ products and to exploit all rights, as set out in our licence.*

*The Submitting Author accepts and understands that any supply made under these terms is made by BMJ to the Submitting Author unless you are acting as an employee on behalf of your employer or a postgraduate student of an affiliated institution which is paying any applicable article publishing charge ("APC") for Open Access articles. Where the Submitting Author wishes to make the Work available on an Open Access basis (and intends to pay the relevant APC), the terms of reuse of such Open Access shall be governed by a Creative Commons licence – details of these licences and which Creative Commons licence will apply to this Work are set out in our licence referred to above.*

*Other than as permitted in any relevant BMJ Author's Self Archiving Policies, I confirm this Work has not been accepted for publication elsewhere, is not being considered for publication elsewhere and does not duplicate material already published. I confirm all authors consent to publication of this Work and authorise the granting of this licence.*

For peer review only

# Reducing cardiovascular disease risk amongst families with familial hypercholesterolaemia by improving diet and physical activity: A randomised controlled feasibility trial

Fiona J Kinnear[1], Fiona E Lithander[1, 2], Aidan Searle[1], Graham Bayly[3], Christina Wei[4], David J Stensel[5,6], Alice E Thackray[5, 6], Linda Hunt[1], Julian P Hamilton-Shield[1].

1. National Institute for Health Research Bristol Biomedical Research Centre, University Hospitals Bristol and Weston NHS Foundation Trust and University of Bristol, Bristol, UK.
2. Bristol Medical School, The University of Bristol, Bristol, UK
3. Department of Clinical Biochemistry, University Hospitals Bristol and Weston NHS Foundation Trust, Bristol, UK
4. St George's University Hospitals NHS Foundation Trust, London, UK
5. National Centre for Sport and Exercise Medicine, School of Sport, Exercise and Health Sciences, Loughborough University, Epinal Way, Loughborough LE11 3TU, UK
6. National Institute for Health Research (NIHR) Leicester Biomedical Research Centre, University Hospitals of Leicester NHS Trust and University of Leicester, Leicester, UK

Corresponding author:
Fiona Kinnear, NIHR Bristol Biomedical Research Centre Nutrition Theme,
University Hospitals Bristol and Weston NHS Foundation Trust and the University of Bristol, Bristol, UK. Email:Fiona.kinnear@bristol.ac.uk

**Word count: 4028**

For peer review only

For peer review only


For peer review only - http://bmjopen.bmj.com/site/about/guidelines.xhtml

## Abstract

**Objective** Familial hypercholesterolaemia (FH) elevates low-density lipoprotein cholesterol (LDL-C) and increases cardiovascular disease (CVD) risk. This study aimed to provide evidence for the feasibility of conducting a randomised controlled trial to evaluate the efficacy of an intervention designed to improve diet and physical activity in families with FH.

**Design** A parallel, randomised, wait-list controlled, feasibility pilot trial.

**Setting** Three outpatient lipid clinics in the UK.

**Participants** Families comprised of children (10-18 years) and their parent with genetically diagnosed FH.

**Intervention** Families were randomised to either 12-week usual-care or intervention. The behavioral change intervention aimed to improve dietary, physical activity and sedentary behaviours. It was delivered to families by dietitians initially via a single face-to face session and then by four telephone or email follow-up sessions.

**Outcome measures** Feasibility was assessed via measures related to recruitment, retention, and intervention fidelity. Post-intervention qualitative interviews were conducted to explore intervention acceptability. Behavioural (dietary intake, physical activity and sedentary time) and clinical (blood pressure, body composition and blood lipids) outcomes were collected at baseline and endpoint assessments to evaluate the intervention's potential benefit.

**Results** Twenty-one families (38% of those approached) were recruited, comprised of 22 children and 17 adults with FH, and 97% of families completed the study. The intervention was implemented with high fidelity and the qualitative data revealed it was well accepted. Between group differences at the endpoint assessment were indicative of the intervention's potential for improving diet in children and adults. Evidence for potential benefits upon physical activity and sedentary behaviours were less apparent. However, the intervention was associated with improvements in several CVD risk factors including LDL-C, with a within group mean decrease of 8% (children) and 10% (adults).

**Conclusions** The study's recruitment, retention, acceptability, and potential efficacy supports the development of a definitive trial, subject to identified refinements.


Trial registration number: ISRCTN24880714, URL: https://doi.org/10.1186/ISRCTN24880714

**Abstract word count: 299**



For peer review only - http://bmjopen.bmj.com/site/about/guidelines.xhtml

## Strengths and limitations of this study

- This is the first study to assess the feasibility and acceptability of delivering a behaviour change intervention, which aims to enable attainment of the dietary and physical activity treatment recommendations, to families affected by familial hypercholesterolaemia (FH).
- The quantitative and qualitative measures employed in this multi-centred, randomised controlled trial have provided evidence of the feasibility and acceptability of conducting a future adequately powered trial, subject to identified refinements.
- An adequately powered, randomised controlled trial is required to formally evaluate the effectiveness of the intervention upon behavioural and clinical outcomes.
- The sample was comprised of mostly highly educated, white European participants and the findings may not be generalisable to all individuals living with FH


For peer review only - http://bmjopen.bmj.com/site/about/guidelines.xhtml

## Introduction

Familial hypercholesterolaemia is a genetic disease, characterised by elevated levels of low-density lipoprotein cholesterol (LDL-C) from birth.[1] The disease confers an 18-fold increased risk of cardiovascular disease (CVD), accounting for one in every 17 CVD cases worldwide.[2] The heterozygous genotype of familial hypercholesterolaemia, henceforth called FH, affects one in 311 individuals globally. (2) Pharmacological treatment significantly reduces LDL-C, and thus CVD risk, especially when initiated in childhood.[3] However, CVD risk remains elevated and its onset and severity varies substantially.[4,5]

Environmental factors, including lifestyle behaviours, may explain some of the variance in CVD risk,[6] mediated via LDL-C reductions additive to that attained by pharmacological treatment or effects on other CVD risk factors.[7,8] Both pathways are important as LDL-C treatment goals are often not achieved[9] and hypertension, type II diabetes mellitus (T2DM) and obesity are independently associated with CVD risk amongst adults with FH.[7] Accordingly, adjuvant to pharmacological therapy, clinical guidelines recommend individuals with FH should be encouraged to be physically active, maintain a healthy weight and consume a healthy diet.[10] There is no evidence from randomised controlled trials (RCTs) demonstrating the effectiveness of these recommendations on CVD risk in individuals with FH.[11,12]

A previous RCT sought to address this evidence gap,[13] but the intervention was implemented with poor fidelity and failed to significantly improve dietary or physical activity behaviours.[14] Less than half of children and adults with FH adhere to dietary and physical activity treatment recommendations,[15,16] with several factors identified as barriers including the asymptomatic nature prior to onset of CVD and the perception that it is unnecessary when also taking medication.[17] In order to explore the true effects of diet and physical activity upon CVD risk, it is essential that interventions can successfully change these behaviours. Given the high prevalence of FH, any intervention must also be feasible to implement within the time and resource constraints of health services.[10] Therefore, we previously developed a pragmatic behaviour change intervention specifically designed to address the determinants of dietary and physical activity behaviours amongst individuals with FH.[18] As the



For peer review only - http://bmjopen.bmj.com/site/about/guidelines.xhtml

inheritance pattern of FH means all affected children will have one affected parent, it was delivered to families.

According to the Medical Research Council (U.K.) guidance,[19] healthcare interventions should undergo feasibility testing prior to evaluation in a full scale RCT. Therefore, this trial aimed to provide evidence for the feasibility and acceptability of conducting a future, adequately powered RCT and identify any refinements to the RCT design or intervention required. The six objectives were to:

1. Assess the feasibility of recruiting, randomising, and retaining families with FH
2. Evaluate the feasibility of collecting valid outcome measures
3. Assess the feasibility of implementing the intervention with sufficient fidelity
4. Explore the participant acceptability of the intervention
5. Explore the potential effectiveness of the intervention upon behavioural and clinical outcomes
6. Estimate the sample size required to adequately power a future RCT


For peer review only - http://bmjopen.bmj.com/site/about/guidelines.xhtml

## Methods

The trial received ethical approval from the Cornwall and Plymouth Research Ethics Committee (reference: 18/SW/0121). The study protocol is published,[20] and a CONSORT checklist[21] displayed in supplementary file 1.

### *Trial design and setting*

The study was a parallel, two-arm, randomised, controlled, feasibility pilot trial[22] comparing a diet and physical activity intervention against usual-care waitlist control amongst families living with FH. It was conducted across three National Health Service (NHS) Foundation Trust sites in England, U.K.

### *Participants*

The study aimed to recruit families comprised of a child aged 10-18 years and their parent, both with a genetic diagnosis of FH and established on their current treatment (lifestyle and/or pharmacological) regimen ≥ 1 month. Families could be comprised of multiple affected children and non-affected parents or carers in instances in which affected parents were unwilling, unable or ineligible to participate. If this was not possible, children could participate without parental involvement.

### *Recruitment and randomisation*

All eligible children receiving care from outpatient lipid clinics at the study sites were invited to participate, along with their parent, via invitation letters and/or when attending routine clinic appointments. Written informed consent (or assent and parental consent for children under 16 years old) was obtained from all participants. After baseline data collection, families were randomised to receive usual care and waitlist control or the intervention on a 1:1 basis, stratified by study site. This was carried out by a database manager using pre-prepared, password protected, randomised lists. It was not possible to blind participants or research staff to the randomisation.

### *Sample size*

As this was a feasibility study, a formal sample size calculation was not required.[21] The recruitment of 24 families, based upon the local number of eligible families, was deemed to be large enough to address the study objectives.



For peer review only - http://bmjopen.bmj.com/site/about/guidelines.xhtml

### Intervention

The development and content of the behaviour change intervention are described in detail elsewhere[18] and in supplementary file 2. In brief, the intervention aimed to enable individuals to reduce dietary intakes of total fat, saturated fat and cholesterol; increase intakes of unsaturated fats, fibre, fruits, vegetables and plant stanol or sterol fortified foods; reduce sedentary behaviour and increase physical activity. The Behaviour Change Wheel, which brings together 19 different theories of behaviour change,[23] was applied to the findings of a qualitative evidence synthesis to identify the determinants of treatment behaviours.[17] Twenty-six behavioural change techniques (BCTs) were then incorporated into the intervention to target these.[18] The intervention was delivered to families by one of two dietitians as an initial 1-hour face-to-face session and four email or telephone follow-up sessions over a 12-week intervention period. The dietitians received comprehensive manuals which detailed the content and BCTs to be delivered to families at each session (supplementary file 2).

*Comparator (Waitlist, usual care control)*

Families were informed that they were on a waitlist to receive the intervention at the end of the 12-week study period and received usual care, which comprised of an annual outpatient lipid clinic appointment. Adults, but not children, in both groups had previously received dietetic counseling upon FH diagnosis as part of usual care. No participant received any further dietetic advice during the trial control period.

*Pharmacological treatment*

Participants in both groups were instructed to continue with their prescribed pharmacological treatment, if applicable. Any self-reported changes to medication were documented and validated through confirmation from medical records.

### Outcome measures

*Feasibility outcomes (study objectives 1-3)*

Recruitment, randomisation and retention rates were collected for families, as the study aimed to recruit children and their parent. Checklists completed by dietitians after each intervention session were analysed to produce outcomes related to the *dose* (number and duration of sessions delivered) and *fidelity* (inclusion of pre-specified content sections and BCTs) of the intervention delivered to each family.[24] Dietitians documented any adaptations made to the intervention. The proportion of participants

For peer review only



For peer review only - http://bmjopen.bmj.com/site/about/guidelines.xhtml

from whom valid outcome measures were collected was used to assess the feasibility of obtaining these measures. While outcome measures were collected from all adults, data is only analysed and presented for adults affected with FH.

*Acceptability outcomes (study objective 4)*

After study completion, 10 families took part in face-to-face or telephone qualitative interviews to explore the acceptability of the intervention. Purposive sampling was employed to yield maximum variation within this sub-sample. The sample included four families from the control group who were interviewed after they received the intervention upon study completion. All interviews were conducted by FJK and were audio-recorded and transcribed *verbatim.*

*Behavioural and clinical outcomes (study objectives 5 & 6)*

It was not appropriate to test the effectiveness hypothesis, as the study was not sufficiently powered to do so.[25] However, all outcome measures to be assessed in a future trial were measured and the potential effectiveness of the intervention on behavioural (diet, physical activity and sedentary behaviours) and clinical (selected CVD risk factors and quality of life) outcomes was explored. Clinical outcomes were measured by the research team at baseline and endpoint assessment visits before and after the 12-week intervention or control period. Behavioural outcomes were measured in the week preceding these visits. A summary is provided below, with full details elsewhere[20] and supplementary file 3.

Participants recorded their dietary intake on four non-consecutive days (including one weekend day) using Intake24, a validated online 24-hour recall tool.[26,27] At least three days were required for analysis and data were analysed to produce outcome measures related to the intervention dietary targets. Participants wore two accelerometers (ActiGraph GT3X+ and activPAL3) for seven consecutive days to measure moderate and vigorous physical activity (MVPA) and time spent being sedentary during waking hours. At least four valid days (including one weekend day) were required for analysis.

Height was measured using a stadiometer and weight (kg), body fat (%) and fat free mass (kg) using bioelectrical impedance scales (Tanita™ MC-780MA). At sites


For peer review only - http://bmjopen.bmj.com/site/about/guidelines.xhtml

where this equipment was unavailable, weight was measured using medical scales. BMI was determined from the height and weight measurements and systolic and diastolic blood pressures were measured using automated oscillometric devices (Dinamap, V100). Health related quality of life (HRQoL) was measured in children using a Pediatric Quality of Life Inventory ™ (PedsQL™) and in adults using the EuroQol Group EQ-5D-3L health questionnaires.[28,29] Fasted blood samples were collected to measure total cholesterol, triglyceride and HDL-C using standard methodology and reagents on local NHS laboratory autoanalysers (Details in supplementary file 3). LDL-C was estimated using the Friedewald equation.[30] The results of lipidomic analyses will be reported in the future, due to the closure of the relevant metabolomics laboratory because of the coronavirus.

***Data analysis***

Feasibility outcomes were analysed descriptively and the qualitative outcomes analysed using the six stages of thematic analysis described by Braun and Clarke.[31] The analysis of the behavioural and clinical outcomes were carried out separately for children and adults. For parametric outcomes, the baseline and endpoint measures for intervention and control groups were summarised with means and standard deviations (S.D.). The endpoint means in the intervention and control groups were compared with and without adjustment for the baseline values using regression analysis,[32] and their differences reported with 95% confidence intervals (C.I.). For non-parametric outcomes, baseline and endpoint measures and within-group pre-to-post changes for intervention and control groups were summarised by median and range. To maximise the power of the models, all valid data collected at baseline and endpoint assessments were included in the analyses.

**Patient involvement**

As detailed in the protocol,[20] patients with FH, under the care of the University Hospitals Bristol NHS Foundation Trust, participated in the development work underpinning this research. They gave feedback about the initial design of the intervention and data collection methods.


For peer review only - http://bmjopen.bmj.com/site/about/guidelines.xhtml

## Results

### *Recruitment, randomisation and retention (study objective 1)*

Recruitment took place between August 2018 and December 2019 and ceased due to study timelines. Recruitment to the trial was 38% of eligible families, with 21 of the target 24 families recruited, 18 (86%) of which comprised of a child and their parent with FH (Figure 1). All families were successfully randomised and retention was high at 97%. The baseline characteristics of the participants (with FH) are displayed in Table 1.

### *Collection of clinical and behavioural outcome measures (study objective 2)*

All families who completed the study attended all research visits, although one child was unable to accompany their parent to the endpoint assessment. Clinical outcome measures were collected from all other participants, with the exception of body composition, due to unavailability of the required equipment at two of the three sites, and blood lipid profile, due to needle phobia (n=2; children), nurse unavailability (n=3; 2 child, 1 adult) and unsuccessful venepuncture (n=2; 1 child, 1 adult). All adults selected the maximum values in the HRQoL questionnaires and due to this ceiling effect, the data were not analysed. Valid paired (baseline and endpoint) dietary outcome measures were obtained from >80% of participants, with missing data due to participants (n=5; 4 child, 1 adult) not completing the required minimum three days of dietary recalls. Due to battery malfunctions (n=12; 6 child, 6 adult) and non-wear due to skin reactions (n=3; children), paired sedentary outcome measures were only collected from 57% and 65% of children and adults, respectively.

### *Intervention implementation and fidelity outcomes (study objective 3)*

All families received the initial intervention session and the duration averaged 66 minutes, 10% higher than the intended 60 minutes. All families received the first three follow-up sessions, only 60% of families opted to receive the fourth follow-up session. Two families chose to receive follow-up sessions via email and eight opted for telephone with an average duration of 25 minutes, which was below the intended 30 minutes. The fidelity to the content and BCTs intended to be delivered in each session was high, with only one content section (*'barriers and solutions', detailed in supplementary file 2)* not delivered to 20% of families, due to the initial session exceeding the intended 60 minutes and the family having to leave. In response to the individual needs of the families, four BCTs were not delivered to all families and 80%



For peer review only - http://bmjopen.bmj.com/site/about/guidelines.xhtml

of families received additional BCTs during the follow-up sessions. Two further BCTs were not delivered to 20% of families, as the required information (dietary intake and body composition data) was unavailable for the initial session. Supplementary file 2 provides details of these omitted and additional BCTs.

***Acceptability results (study objective 4)***

Overall the participants were positive towards the content of the intervention, which was perceived to have provided the knowledge and motivation to change behaviours: *'If no-one's telling you what you have to change, then it's harder to think about it.'* (parent). The follow-up sessions were valued as they provided additional information and/or motivational support: *'If we'd not had any contact, it might have been difficult to keep on track.'* (parent). The step-wise approach to goal setting and the family-based delivery of the intervention facilitated behaviour change: *'it's just easier if you have someone else doing it with you.'* (child). If suitable alternatives could be identified, the dietary goals were perceived to be easy to implement and participants intended to continue with the swaps: *'I got used to the cheese and the brown pasta. It just tastes better now than the other pasta.'* (child). Having their child enjoy these foods increased acceptability: *'To see her eating broccoli and green beans, it's what every mum wants.'* (adult). For those who were inactive previously, changing physical activity behaviours proved harder to implement, requiring *'discipline'* (adult) and if they did not find an activity they enjoyed, many continued to struggle. The acceptability of changing behaviours was harder when routines changed during school holidays. However, participants viewed these as short-term lapses and returned to their new habits after: *'The summer holidays have been tricky because you're always out of a routine. When we're back at school it will be easier.'* (adult).

For peer review only

***Behavioural and clinical outcomes (study objectives 5 & 6)***

The baseline data collected from the withdrawn participant was included in the analysis. The results suggest that the intervention may be effective in decreasing intake of total fat, saturated fat and cholesterol, and increasing intake of fibre, fruits and vegetables (Table 2). No participants in either group consumed plant stanol or sterol fortified products at baseline. This remained true for the control group, whereas all but one participant in the intervention group reported consuming a yogurt containing 2g of plant stanols or sterols everyday throughout the intervention. The within-group median change between baseline and endpoint assessment is indicative



For peer review only - http://bmjopen.bmj.com/site/about/guidelines.xhtml

of a positive effect of the intervention upon increasing daily MVPA and decreasing daily sedentary time in adults, but not children (Table 2). However, the data suggests MVPA also increased amongst adults in the control group meaning it is not possible to attribute the improvements solely to the intervention. The direction of trends suggests a favourable effect of the intervention on diastolic blood pressure, total cholesterol and LDL-C in adults and children. (Table 2) Additionally, the within-group median change suggests that the intervention may be associated with improvements in BMI, body composition and HRQoL in children (Table 3).

The within group mean decrease in LDL-C for children in the intervention group was 0.29 (0.58) mmol/l and for adults was 0.33 (0.77) mmol/l, which is an approximate decrease of 8 and 10% from baseline, respectively. A LDL-C reduction of 10% was deemed to be of clinical significance and the sample size calculation was based upon this. Using the mean baseline LDL-C and SDs in children in the intervention group, and a 5% level of significance, a sample size of 42 children in each group was calculated to have 80% statistical power to show a difference between a 10% decrease in LDL-C and no difference in the control group. Using the same methods, a sample size of 68 adults in each group was calculated. To account for non-collection of behavioural outcomes and participation of families with non-affected parents, a future trial should aim to recruit 160 families. Using the 38% recruitment rate, this would require a potential recruitment pool of 420 eligible families.



For peer review only - http://bmjopen.bmj.com/site/about/guidelines.xhtml

## Discussion

This trial has demonstrated that it is feasible to recruit, randomise and retain families with FH into a 12-week trial, and to implement and evaluate a dietary and physical activity intervention, which is acceptable to families. While this study was not sufficiently powered, the potentially favourable effects upon CVD risk factors are encouraging and warrant further investigation in a full-scale RCT, subject to the identified refinements.

The 8-10% reduction in LDL-C that that was observed in this trial is clinically significant. It is in fact comparable to that achieved through the addition of further medication, such as one that inhibits the intestinal absorption of cholesterol, to standard statin therapy.[33] Many children and adults with FH do not meet LDL-C treatment targets, even when using maximally licensed doses of medication.[3] There is no LDL-C concentration below which the beneficial effects upon CVD risk cease to improve,[34] and a 'the lower, the better' approach is advocated.[10] Any additional LDL-C reduction that can be achieved through non-pharmacological approaches is critically important, highlighting the potential benefit of the LDL-C finding.

Most families in this trial were recruited at paediatric lipid clinics, held every three to six months. This infrequency contributed to the recruitment target not being met within study timelines, a common finding in RCTs.[35] The infrequent clinics are representative of the low number of individuals diagnosed with FH in the U.K., (8%) and worldwide (1-15%).[36] Ongoing efforts to increase the detection of FH through cascade testing continue to improve these statistics,[36] which should increase the number of eligible families and clinics in a future trial. The study recruited 38% of eligible families, higher than the 34% reported in a similar study conducted in adults with FH[37] and the 22% reported in a family-based study to reduce CVD risk in children without FH.[38] Recruitment could be improved by telephoning non-responders[39] and incorporating a research visit into a lipid clinic appointment to reduce the time burden on participants, a common barrier to participation in this study. Once recruited, the acceptability of the trial was high regardless of randomisation, as reflected by retention of 97%. This study has also demonstrated the feasibility of non-affected parents participating in the trial when the affected parent cannot. As treatment is most beneficial when initiated in childhood,[10] and the family-



For peer review only - http://bmjopen.bmj.com/site/about/guidelines.xhtml

based delivery of the intervention was integral to the participants' acceptability, non-affected parents should continue to be eligible to facilitate recruitment of children in a future trial.

Participant acceptability and compliance with recording dietary and physical activity behaviours was higher than previous research.[40, 41] Battery malfunction due to the age of the accelerometers used, accounted for most missing sedentary behaviour outcomes and a future trial should use new or recently refurbished activPALs. The low collection of body composition outcomes can be overcome in a future trial by providing sites with the equipment and the updated EQ-5D-5L version of the adult QoL questionnaire should be used, which reduces the occurrence of the ceiling effect found in this study.[42]

In contrast to a previous trial conducted in adults with FH,[14] the intervention was implemented with high fidelity, likely due to the comprehensive manuals created. The adaptations made were almost all in response to the individual needs of families. If adaptations do not undermine the mechanisms of action by which the intervention is proposed to have its effect, tailoring interventions to suit different contexts can have beneficial effects upon the outcomes.[43] As the intervention was systematically developed,[18] the effect of adaptations can be evaluated in a definitive trial.[21]

Participants' dietary intakes fell short of treatment recommendations at baseline, as reported by others previously.[13,44] This was despite all adults having previously received dietetic advice upon diagnosis, suggesting the specifically designed behaviour change intervention has advantages over routine advice provided in outpatient lipid clinics. The qualitative findings serve to demonstrate that the intervention was highly acceptable to families with FH, who were able and willing to change their lifestyle behaviours. This was largely driven by the family-based approach, also adopted by a previous non-randomised trial, which successfully improved dietary behaviours of children with FH.[44] However, only the behaviours of the children were targeted and eight dietetic sessions were provided.[44] While not powered to detect changes, the findings suggest that the pragmatic intervention evaluated in the current study may achieve dietary improvements in both adults and children without the need for multiple dietetic sessions.


For peer review only - http://bmjopen.bmj.com/site/about/guidelines.xhtml

Unlike previous research,[14] the results suggest physical activity and sedentary behaviours of adults with FH may be improved by the intervention developed for the current study. However, trends towards improvement amongst adults in the control group were also observed. This finding may be explained by the *reactivity bias* associated with wearing accelerometers, in which participants modify their habitual physical activity in response to wearing accelerometers.[45] In contrast, the results of this study do not suggest that physical activity or sedentary behaviours were improved amongst children in either group. Participants received feedback about the disparities between their dietary intakes and the recommendations, but not for physical activity. Parents of inactive children typically over-estimate the time they spend in physical activity, as do the children themselves.[46] Given the low rates of children meeting the physical activity recommendations in this study, feedback on current physical activity levels in comparison to the recommendations should be provided in a future trial to increase motivation to change this behaviour.

### ***Strengths and limitations***

The participants were highly educated and mostly white Europeans, limiting the generalisability of the findings to all families with FH, a common condition affecting all ethnicities.[2] The qualitative sample did not include any participants of different ethnicities and it cannot be concluded that data saturation was achieved, limiting the validity of the qualitative findings.[47] The 12-week follow-up period in this study does not enable exploration of longer-term effects, which are of importance to treatment for a life-long condition like FH.[11] Individuals attend annual lipid clinic appointments and it would be feasible to extend the follow-up period to 12 months in a future trial. The dietary intake estimates in this study are limited by the misreporting bias associated with all self-report methods of dietary assessment.[48] A future trial should include objective measures of dietary intakes to validate the estimates of the nutrients targeted by the intervention, such as blood concentrations of fatty acids and phytosterols.[49] Adherence to pharmacological treatment influences LDL-C concentrations and this was not formally assessed in this study. The validated Morisky Eight-item Medication Adherence scale is suggested for a future trial.[50]


For peer review only - http://bmjopen.bmj.com/site/about/guidelines.xhtml

## Conclusion

This study has addressed the main uncertainties ahead of a definitive trial and if the identified refinements are made to the study design then a fully powered RCT is feasible and warranted. Such a trial will address the unanswered question of the potential benefits of the current diet and physical activity treatment recommendations upon LDL-C treatment goals and CVD risk in children and adults with FH.

For peer review only



For peer review only - http://bmjopen.bmj.com/site/about/guidelines.xhtml

**Acknowledgements** We would like to thank Dr Coxson, Dr Edmonds and all members of the research teams at University Hospitals Bristol and Weston, St. George's University Hospitals and Royal United Hospitals Bath NHS Foundation Trusts. DJS and AET acknowledge support from the National Institute for Health Research NIHR Leicester Biomedical Research Centre. The views expressed are those of the authors and not necessarily those of the NHS, the NIHR or the Department of Health and Social Care.

**Contributors:** FJK, JHS, FEL, GB, CL and DS contributed to the design of the study. FJK and AS analysed and interpreted the qualitative data. FJK, AET, FEL, JHS analysed and interpreted the quantitative data. FJK and LH devised and conducted the statistical analysis plan for this analysis. FJK prepared the manuscript, which was critically revised by all co-authors and all authors approved the final version.

**Funding** This study was funded by the NIHR Biomedical Research Centre at University Hospitals Bristol and Weston NHS Foundation Trust and the University of Bristol. Award/grant number: not applicable. The views expressed are those of the authors and not necessarily those of the NIHR or the Department of Health and Social Care.

**Competing interests** All authors declare that they have no competing interests.

**Data sharing statement:** Data from this study are available on request.

**Figure legends**

Figure 1. Consolidated Standards of Reporting Trials (CONSORT) diagram of recruitment of families across the three sites.

**Supplementary files**

Supplementary file 1. CONSORT 2010 checklist of information to include when reporting a pilot or feasibility trial

Supplementary file 2. Additional details of intervention content and delivery

Supplemental file 3. Additional details of outcome measures used in study



For peer review only - http://bmjopen.bmj.com/site/about/guidelines.xhtml

# Tables

## Table 1 Baseline characteristics of the study participants*

| Characteristic | Adults | | Children | |
|---|---|---|---|---|
| | Control (n=9) | Intervention (n=8) | Control (n=12) | Intervention (n=10) |
| **Gender- n female (%)** | 4 (44%) | 4 (50%) | 6 (50%) | 5 (50%) |
| **Age (years)- mean (SD); range** | 51 (5); 43-59 | 42 (6); 32-49 | 15 (3) 10-18 | 13 (2); 10-16 |
| **Ethnicity- n (%)** | | | | |
| *White European* | 8 (89%) | 7 (88%) | 9 (75%) | 9 (90%) |
| *Asian* | 1 (11%) | 1 (12%) | 3 (25%) | 1 (10%) |
| **Managerial or professional job- n (%)** | 7 (78%) | 8 (100%) | - | - |
| **Treatment- n (%)** | | | | |
| *Lifestyle only* | 0 | 1 (13%) | 2 (17%) | 3 (30%) |
| *Statin medication* | 2 (22%) | 2 (25%) | 10 (80%) | 7 (70%) |
| *Statin and Ezetimibe medication* | 6 (67%) | 5 (63%) | 0 | 0 |
| *PCSK9 inhibitor injections* | 1 (11%) | 0 | 0 | 0 |
| **FH pathogenic variant location- n (%)** | | | | |
| *LDLR* | 7 (78%) | 5 (63%) | 10 (83%) | 7 (70%) |
| *APOB* | 2 (12%) | 2 (25%) | 2 (17%) | 2 (20%) |
| *PCSK9* | 0 | 1 (12%) | 0 | 1 (10%) |
| **Risk factors – n (%)** | | | | |
| *Current smoker* | 0 | 1 (13%) | 0 | 0 |
| *Previous smoker* | 4 (44%) | 1 (13%) | 0 | 0 |
| *Hypertension* | 2 (22%) | 1 (13%) | 0 | 0 |
| *Overweight weight status†* | 4 (44%) | 4 (50%) | 2 (17%) | 2 (20%) |
| *Obese weight status ‡* | 0 | 0 | 1 (8%) | 1 (10%) |

N, number; SD, standard deviation; LDLR, low density lipoprotein receptor; APOB, Apolipoprotein B; PCSK9, Proprotein convertase subtilisin/kexin type 9

*Only adults with FH included in this table

† Overweight in adults defined as BMI ≥ 25-29.9kg/m$^2$ and BMI centile ≥ 91st percentile for children

‡ Obese defined as BMI ≥ 30kg/m$^2$ for adults and BMI centile ≥ 98th percentile for children

For peer review only



For peer review only - http://bmjopen.bmj.com/site/about/guidelines.xhtml

Table 2. Mean daily intakes of nutrients and clinical outcome measures (SBP, DBP, total cholesterol, LDL-C, HDL-C and HRQoL) at baseline and endpoint assessments for control and intervention groups and a comparison of the differences between groups at the endpoint assessment unadjusted and adjusted for baseline values*

| | | Children | | | | | | | Adults | | | | | | |
|---|---|---|---|---|---|---|---|---|---|---|---|---|---|---|---|
| Outcome | Group | N | Baseline | N | Endpoint | Endpoint difference (95% CI) | N | Adujsted difference† (95% CI) | N | Baseline | N | Endpoint | Endpoint difference (95% CI) | N | Adujsted difference† (95% CI) |
| Fat intake (% TEI) | Intervention | 9 | 33.0 (6.4) | 9 | 28.3 (5.1) | -5.6 | 9 | -5.3 | 8 | 35.4 (7.8) | 8 | 31.2 (5.9) | -0.3 | 8 | -2.3 |
| | Control | 10 | 32.9 (4.8) | 9 | 33.9 (4.1) | (-10.2 to -0.9) | 8 | (-8.9 to -1.5) | 9 | 32.0 (5.7) | 8 | 31.6 (6.3) | (-6.9 to 6.2) | 8 | (-7.4 to 2.9) |
| SFA intake (%TEI) | Intervention | 9 | 12.6 (2.0) | 9 | 10.3 (2.6) | -1.8 | 9 | -1.8 | 8 | 12.1(3.8) | 8 | 9.9 (2.6) | -1.1 | 8 | -1.9 |
| | Control | 10 | 12.1 (2.9) | 9 | 12.1 (2.1) | (-4.2 to 0.5) | 8 | (-4.3 to 0.8) | 9 | 10.8 (3.4) | 8 | 10.9 (3.3) | (-4.3 to 2.2) | 8 | (-4.3 to 0.5) |
| MUFA intake (%TEI) | Intervention | 9 | 11.6 (4.1) | 9 | 9.4 (2.0) | -2.8 | 9 | -3.2 | 8 | 13.5 (3.7) | 8 | 11.5 (3.4) | 0.9 | 8 | 0.3 |
| | Control | 10 | 11.3 (1.8) | 9 | 12.2 (2.3) | (-4.9 to -0.6) | 8 | (-5.3 to -1.01) | 9 | 12.1 (2.6) | 8 | 10.6 (3.5) | (-2.8 to 4.6) | 8 | (-3.3 to 3.9) |
| PUFA intake (%TEI) | Intervention | 9 | 4.8 (1.4) | 9 | 4.8(1.1) | -0.4 | 9 | -0.6 | 8 | 5.7 (1.5) | 8 | 5.6 (1.6) | 0.1 | 8 | 0.03 |
| | Control | 10 | 4.6 (1.1) | 9 | 5.3 (1.3) | (-1.7 to 0.8) | 8 | (-1.9 to 0.7) | 9 | 5.2 (1.4) | 8 | 5.5 (2.2) | (-1.9 to 2.2) | 8 | (-2.1 to 2.2) |
| Cholesterol intake (mg) | Intervention | 9 | 181.9 (71.2) | 9 | 149.4 (54.6) | -34.1 | 9 | -24.1 | 8 | 219.2 (84.6) | 8 | 204.4 (30.5) | 63.9 | 8 | 28.1 |
| | Control | 10 | 220.9 (95.2) | 9 | 183.5 (34.7) | (-117.2 to 48.7) | 8 | (-100.9 to 52.7) | 9 | 160.9 (60.1) | 8 | 37.4 (105.8) | (-39.6 to 167.5) | 8 | (-76.6 to 132.8) |
| Fibre intake (g) | Intervention | 9 | 20.0 (5.2) | 9 | 24.2 (6.4) | 7.8 | 9 | 5.2 | 8 | 15.5 (5.7) | 8 | 20.6 (7.8) | -2.1 | 8 | 1.1 |
| | Control | 10 | 15.4 (4.0) | 9 | 16.3 (5.7) | (1.8 to 13.9) | 8 | (-0.7 to 10.9) | 9 | 22.4 (10.6) | 8 | 22.6 (7.4) | (-10.2 to 6.1) | 8 | (-6.7 to 8.8) |
| Fruit & vegetable intake (portions) | Intervention | 9 | 3.2 (1.7) | 9 | 4.2 (0.9) | 2.2 | 9 | 2.2 | 8 | 3.3 (2.0) | 8 | 4.1 (1.5) | 1.2 | 8 | 1.6 |
| | Control | 10 | 2.2 (1.1) | 9 | 2.0 (0.6) | (1.4 to 3.0) | 8 | (1.2 to 3.2) | 9 | 4.5 (1.8) | 8 | 2.9 (1.3) | (-0.3 to 2.8) | 8 | (0.1 to 3.1) |
| SBP (mmHg) | Intervention | 10 | 104.5 (10.5) | 9 | 104.9 (6.3) | -3.1 | 9 | -1.6 | 8 | 115.5 (15.1) | 8 | 111.9 (3.6) | -8.1 | 8 | -6.8 |
| | Control | 12 | 110.3 (102) | 11 | 107.9 (8.3) | (-10.9 to 4.8) | 11 | (-8.6 to 5.3) | 9 | 117.5 (14.5) | 9 | 120 (4.5) | (-21.2 to 4.9) | 9 | (-15.9 to 1.4 |
| DBP (mmHg) | Intervention | 10 | 62.9 (6.7) | 9 | 58.9 (4.9) | -4.1 | 9 | -2.2 | 8 | 75.4 (10.1) | 8 | 71.6 (7.2) | -3.2 | 8 | -6.8 |
| | Control | 12 | 68.9 (9.2) | 11 | 63.0 (2.4) | (-10.4 to 2.3) | 11 | (-8.1 to 3.7) | 9 | 69.7 (8.6) | 9 | 74.8 (8.6) | (-11.4 to 5.1) | 9 | (-12.7 to -0.9) |
| Total cholesterol (mmol/l) | Intervention | 8 | 5.2 (1.9) | 8 | 5.0 (1.7) | -0.06 | 8 | -0.3 | 7 | 5.0 (0.9) | 8 | 4.8 (0.3) | -0.08 | 7 | -0.4 |
| | Control | 10 | 4.8 (0.8) | 8 | 4.9 (0.8) | (-1.4 to 1.5) | 8 | (-0.9 to 0.4) | 9 | 4.6 (0.9) | 8 | 4.9 (0.5) | (-1.3 to 1.2) | 8 | (-1.2 to 0.4) |
| LDL-C (mmol/l) | Intervention | 8 | 3.5 (0.8) | 8 | 3.2 (1.6) | -0.2 | 8 | -0.36 | 7 | 3.2 (0.7) | 8 | 2.8 (0.8) | -0.2 | 7 | -0.56 |
| | Control | 10 | 3.2 (0.8) | 8 | 3.3 (0.7 | (-1.5 to 1.1) | 8 | (-0.82 to 0.12) | 9 | 2.7 (0.8) | 8 | 2.9 (1.1) | (-1.2 to 0.8) | 8 | (-1.33 to 0.12) |
| HDL-C (mmol/l) | Intervention | 8 | 1.4 (0.3) | 8 | 1.4 (0.3) | 0.1 | 8 | 0.01 | 7 | 1.47 (0.16) | 8 | 1.39 (0.22) | -0.13 | 7 | -0.17 |
| | Control | 10 | 1.2 (0.3) | 8 | 1.3 (0.1 | (-0.2 to 0.4) | 8 | (-0.1 to 0.2) | 9 | 1.45 (0.27) | 8 | 1.52 (0.09) | (-0.38 to 0.13) | 8 | (-0.38 to 0.04) |
| HRQoL ‡ (Score 0 to 100) | Intervention | 10 | 85.1 (9.8) | 9 | 89.0 (7.8) | 16.7 | 9 | 5.9 | | | | | | | |
| | Control | 12 | 73.4 (9.9) | 11 | 72.3 (3.3) | (7.6 to 25.7) | 11 | (-0.3 to 12.0) | | | | | | | |

N, number; CI, confidence interval; TEI, total energy intake; SFA, saturated fatty acids; MUFA, monounsaturated fatty acids; PUFA, polyunsaturated fatty acids; SBP, systolic blood pressure; DBP, diastolic blood pressure; LDL-C, low density lipoprotein cholesterol; HDL-C, high density lipoprotein; HRQoL, health related quality of life

*All data has been included, the ‘N’ columns indicate the number of participants included in each estimated group mean value and estimated mean differences between groups.

† Adjusted for baseline values, this difference estimate includes only participants for whom paired baseline and endpoint data was collected indicated in the ’N’ column

‡ HRQoL data presented for children only due to ‘ceiling effect’ that occurred in the data collected from adult participants

For peer review only

Table 3. Median (range) values for BMI, body composition, daily MVPA and sedentary time at baseline and endpoint assessments for control and intervention groups and the median change (range) between these timepoints for both groups for children and adults*†

| | | Children | | | | | | Adults | | | | | |
|---|---|---|---|---|---|---|---|---|---|---|---|---|---|
| Outcomes | Group | N | **Baseline median (range)** | **N** | **Endpoint median (range)** | **N** | **Median change (range)** | **N** | **Baseline median (range)** | **N** | **Endpoint median (range)** | **N** | **Median change (range)** |
| BMI (z-score children; kg/m² adults) | Intervention | 10 | 0.71 (-1.46 to 3.14) | 9 | 0.54 (-1.74 to 3.18) | 9 | -0.20 (-0.39 to 0.07) | 8 | 24.9 (21.9 to 29.1) | 8 | 25.5 (22.2 to 28.9) | 8 | 0.1 (-1.31 to 0.68) |
| | Control | 12 | 0.41 (-1.79 to 2.35) | 11 | 0.31 (-1.72 to 1.45) | 11 | 0.07 (-0.28 to 0.30) | 9 | 24.0 (21.7 to 28.1) | 9 | 23.2 (22.5 to 27.5) | 9 | -0.41 (-1.85 to 1.35) |
| Body fat (%) | Intervention | 6 | 21.2 (13 to 24.5) | 5 | 22.4 (12.9 to 23.7) | 5 | -0.1 (-1.0 to 0.9) | 4 | 26.4 (23 to 36.5) | 4 | 26.8 (21.6 to 33.7) | 4 | -0.6 (-2.8 to 0.5) |
| | Control | 6 | 27.9 (14.4 to 37) | 5 | 23.8 (16.3 to 29.2) | 5 | 0.2 (-1.4 to 0.3) | 4 | 29.2 (22.4 to 33.2) | 4 | 26.9 (22.7 to 32.5) | 4 | -0.9 (-3.5 to 0.3) |
| Fat free mass (kg) | Intervention | 6 | 41.3 (25.4 to 56.9) | 5 | 43.1 (29.4 to 56.2) | 5 | -0.6 (-1.0 to -0.3) | 4 | 45.1 (41.8 to 52.9) | 4 | 44.9 (40.8 to 54.4) | 4 | -0.2 (-1 to 1.5) |
| | Control | 6 | 38.2 (27.2 to 43.8) | 5 | 39.1 (29.2 to 47.3) | 5 | 1.6 (0.3 to 3.5) | 4 | 52.4 (40.7 to 58.2) | 4 | 51.8 (40.6 to 58) | 4 | -0.2 (-2.2 to 0.9) |
| Daily MVPA (minutes) | Intervention | 10 | 67.1 (59.9 to 84.6) | 10 | 62.2 (42.2 to 86.2) | 10 | -4.2 (-14.3 to 2.4) | 8 | 37.7 (28.0 to 63.1) | 8 | 47.4 (27.9 to 79.9) | 8 | 4.3 (1.2 to 17.2) |
| | Control | 10 | 51.5 (41.9 to 71.9) | 8 | 45.9 (38.7 to 59.4) | 6 | -5.9 (-19.1 to 12.4) | 8 | 34.5 (21.4 to 59.0) | 8 | 41.9 (24.7 to 65.9) | 7 | 6 (-22.2 to 19.1) |
| Daily sedentary time (minutes) | Intervention | 9 | 551 (497 to 553) | 8 | 560 (526 to 608) | 7 | 14 (-41 to 105) | 8 | 593 (543 to 624) | 6 | 552 (529 to 583) | 6 | -34 (-52 to -7) |
| | Control | 10 | 515 (494 to 633) | 6 | 595 (532 to 674) | 5 | 81 (10 to 160) | 8 | 571 (478 to 619) | 8 | 537 (293 to 679) | 5 | 3.8 (-32 to 19) |

N, number; BMI, body mass index; MVPA, moderate and vigorous physical activity.
*All data has been included, the 'N' columns indicate the number of participants included in each estimated group median value and estimated median within group changes from baseline to endpoint
† Ranges are presented due to small sample sizes.


For peer review only - http://bmjopen.bmj.com/site/about/guidelines.xhtml

**Figures**

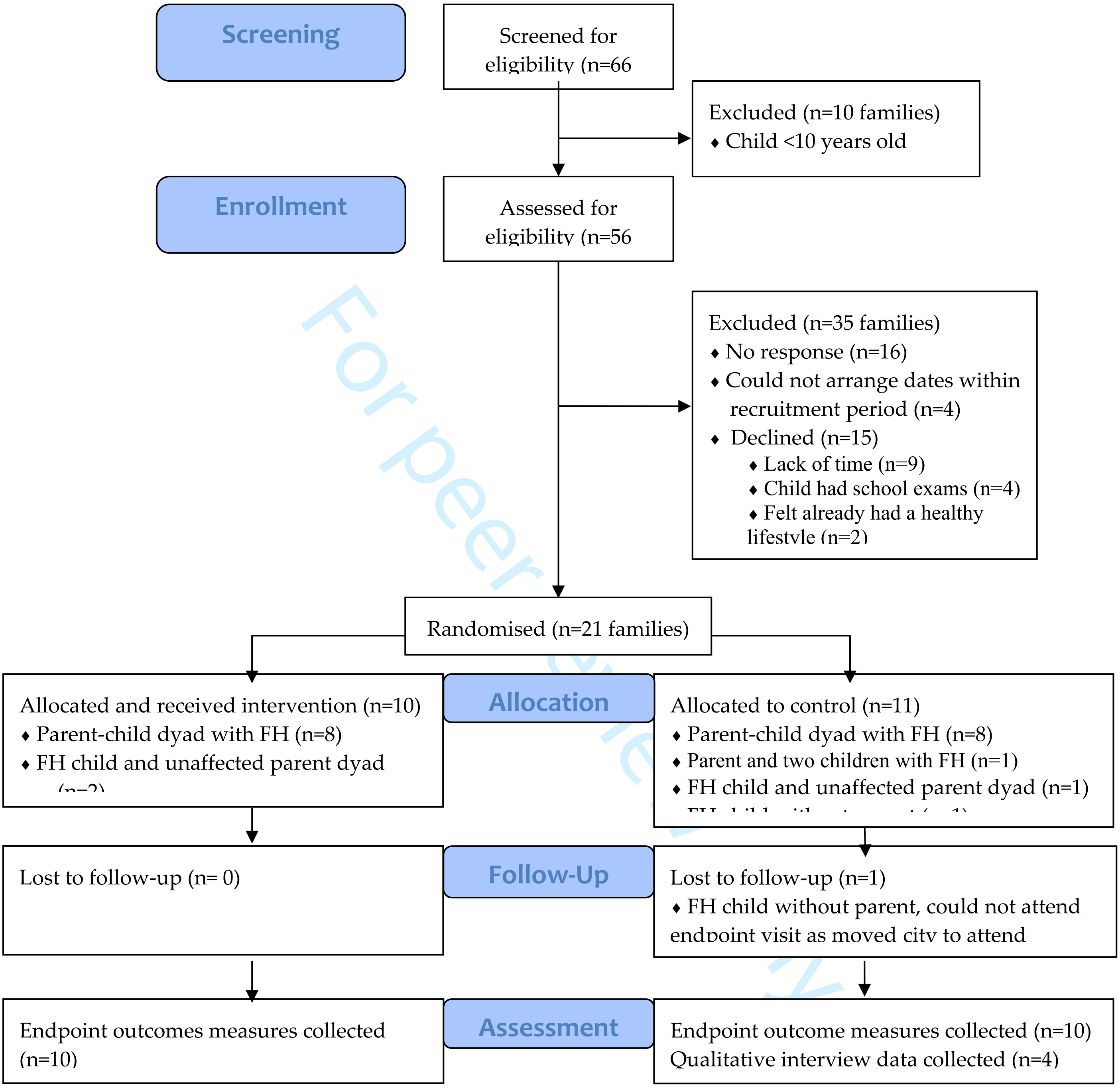


Figure 1. Consolidated Standards of Reporting Trials (CONSORT) diagram of recruitment of families across the three sites.


For peer review only - http://bmjopen.bmj.com/site/about/guidelines.xhtml

## References

1. Goldstein JH, HH; Brown, MS. Familial hypercholesterolemia. In: Sciver CR A, Sly WS, Valle D. editors, editor. The Metabolic and Molecular Bases of Inherited Disease. 8th edition ed. New York: McGraw-Hill; 2001. p. 2863-913.
2. Beheshti SO, Madsen CM, Varbo A, Nordestgaard BG. Worldwide Prevalence of Familial Hypercholesterolemia: Meta-Analyses of 11 Million Subjects. Journal of the American College of Cardiology. 2020;75(20):2553-66.
3. Alonso R, Perez de Isla L, Muñiz-Grijalvo O, Mata P. Barriers to Early Diagnosis and Treatment of Familial Hypercholesterolemia: Current Perspectives on Improving Patient Care. Vasc Health Risk Manag. 2020;16:11-25.
4. Bianconi V, Banach M, Pirro M. Why patients with familial hypercholesterolemia are at high cardiovascular risk? Beyond LDL-C levels. Trends Cardiovasc Med. 2020.
5. Galema-Boers AM, Lenzen MJ, Engelkes SR, Sijbrands EJ, Roeters van Lennep JE. Cardiovascular risk in patients with familial hypercholesterolemia using optimal lipid-lowering therapy. Journal of Clinical Lipidology. 2018;12(2):409-16.
6. Arsenault BJ, Perrot N, Couture P. Does lifestyle contribute to disease severity in patients with inherited lipid disorders? Current opinion in lipidology. 2017;28(2):177-85.
7. Akioyamen LE, Genest J, Chu A, Inibhunu H, Ko DT, Tu JV. Risk factors for cardiovascular disease in heterozygous familial hypercholesterolemia: A systematic review and meta-analysis. J Clin Lipidol. 2018.
8. Mach F, Baigent C, Catapano AL, Koskinas KC, Casula M, Badimon L, et al. 2019 ESC/EAS Guidelines for the management of dyslipidaemias: lipid modification to reduce cardiovascular risk: The Task Force for the management of dyslipidaemias of the European Society of Cardiology (ESC) and European Atherosclerosis Society (EAS). European heart journal. 2019;41(1):111-88.
9. Stock J. First insights from the EAS familial hypercholesterolaemia collaboration registry: FH is still underdiagnosed and undertreated. Atherosclerosis. 2019;290:138-9.
10. Watts GF, Gidding SS, Mata P, Pang J, Sullivan DR, Yamashita S, et al. Familial hypercholesterolaemia: evolving knowledge for designing adaptive models of care. Nat Rev Cardiol. 2020;17(6):360-77.
11. Malhotra A, Shafiq N, Arora A, Singh M, Kumar R, Malhotra S. Dietary interventions (plant sterols, stanols, omega-3 fatty acids, soy protein and dietary fibers) for familial hypercholesterolaemia. Cochrane Database Syst Rev. 2014(6):CD001918.
12. Lozano P, Henrikson NB, Dunn J, Morrison CC, Nguyen M, Blasi PR, et al. U.S. Preventive Services Task Force Evidence Syntheses, formerly Systematic Evidence Reviews. Lipid Screening in Childhood and Adolescence for Detection of Familial Hypercholesterolemia: A Systematic Evidence Review for the US Preventive Services Task Force. Rockville (MD): Agency for Healthcare Research and Quality (US); 2016.
13. Broekhuizen K, van Poppel MN, Koppes LL, Kindt I, Brug J, van Mechelen W. No significant improvement of cardiovascular disease risk indicators by a lifestyle intervention in people with familial hypercholesterolemia compared to usual care: results of a randomised controlled trial. BMC research notes. 2012;5:181.
14. Broekhuizen K, Jelsma JGM, van Poppel MNM, Koppes LLJ, Brug J, van Mechelen W. Is the process of delivery of an individually tailored lifestyle intervention associated with improvements in LDL cholesterol and multiple lifestyle behaviours in people with familial hypercholesterolemia? BMC public health [Internet]. 2012 2012; 12:[348 p.].
15. Avis HJ, Kusters DM, Vissers MN, Huijgen R, Janssen TH, Wiegman A, et al. Follow-Up of Children Diagnosed with Familial Hypercholesterolemia in a National Genetic Screening Program. The Journal of Pediatrics. 2012;161(1):99-103.


For peer review only - http://bmjopen.bmj.com/site/about/guidelines.xhtml

16. Claassen L, Henneman L, Kindt I, Marteau TM, Timmermans DRM. Perceived Risk and Representations of Cardiovascular Disease and Preventive Behaviour in People Diagnosed with Familial Hypercholesterolemia: A Cross-sectional Questionnaire Study. Journal of Health Psychology. 2010;15(1):33-43.
17. Kinnear FJ, Wainwright E, Perry R, Lithander FE, Bayly G, Huntley A, et al. Enablers and barriers to treatment adherence in heterozygous familial hypercholesterolaemia: a qualitative evidence synthesis. BMJ Open. 2019;9(7):e030290.
18. Kinnear FJ, Wainwright E, Bourne JE, Lithander FE, Hamilton-Shield J, Searle A. The development of a theory informed behaviour change intervention to improve adherence to dietary and physical activity treatment guidelines in individuals with familial hypercholesterolaemia (FH). BMC health services research. 2020;20(1):27.
19. Craig P, Dieppe P, Macintyre S, Michie S, Nazareth I, Petticrew M. Developing and evaluating complex interventions: the new Medical Research Council guidance. BMJ. 2008;337.
20. Kinnear FJ, Hamilton-Shield JP, Stensel DJ, Bayly G, Searle A, Thackray AE, et al. Nutrition and physical activity intervention for families with familial hypercholesterolaemia: protocol for a pilot randomised controlled feasibility study. Pilot Feasibility Stud. 2020;6:42.
21. Eldridge SM, Chan CL, Campbell MJ, Bond CM, Hopewell S, Thabane L, et al. CONSORT 2010 statement: extension to randomised pilot and feasibility trials. BMJ. 2016;355:i5239.
22. Eldridge SM, Lancaster GA, Campbell MJ, Thabane L, Hopewell S, Coleman CL, et al. Defining Feasibility and Pilot Studies in Preparation for Randomised Controlled Trials: Development of a Conceptual Framework. PloS one. 2016;11(3):e0150205-e.
23. Michie S, van Stralen MM, West R. The behaviour change wheel: A new method for characterising and designing behaviour change interventions. Implementation Science. 2011;6(1):42.
24. Moore GF, Audrey S, Barker M, Bond L, Bonell C, Hardeman W, et al. Process evaluation of complex interventions: Medical Research Council guidance. BMJ : British Medical Journal. 2015;350:h1258.
25. Sim J. Should treatment effects be estimated in pilot and feasibility studies? Pilot and Feasibility Studies. 2019;5(1):107.
26. Simpson E, Bradley J, Poliakov I, Jackson D, Olivier P, Adamson AJ, et al. Iterative Development of an Online Dietary Recall Tool: INTAKE24. Nutrients. 2017;9(2):118.
27. Bradley J, Simpson E, Poliakov I, Matthews J, Olivier P, Adamson A, et al. Comparison of INTAKE24 (an Online 24-h Dietary Recall Tool) with Interviewer-Led 24-h Recall in 11–24 Year-Old. Nutrients. 2016;8(6):358.
28. Rabin R, de Charro F. EQ-5D: a measure of health status from the EuroQol Group. Ann Med. 2001;33(5):337-43.
29. EuroQoL Research Foundation. EQ-5D-3L User Guide Basic information on how to use the EQ-5D-3L instrument. Version 6.0. . 2018.
30. Friedewald WT, Levy RI, Fredrickson DS. Estimation of the concentration of low-density lipoprotein cholesterol in plasma, without use of the preparative ultracentrifuge. Clin Chem. 1972;18(6):499-502.
31. Braun V, Clarke V. Using thematic analysis in psychology. Qualitative Research in Psychology. 2006;3(2):77-101.
32. Vickers AJ, Altman DG. Analysing controlled trials with baseline and follow up measurements. BMJ. 2001;323(7321):1123.
33. Pisciotta L, Fasano T, Bellocchio A, Bocchi L, Sallo R, Fresa R, et al. Effect of ezetimibe coadministered with statins in genotype-confirmed heterozygous FH patients. Atherosclerosis. 2007;194(2):e116-e22.


For peer review only - http://bmjopen.bmj.com/site/about/guidelines.xhtml

34. Ference BA, Ginsberg HN, Graham I, Ray KK, Packard CJ, Bruckert E, et al. Low-density lipoproteins cause atherosclerotic cardiovascular disease. 1. Evidence from genetic, epidemiologic, and clinical studies. A consensus statement from the European Atherosclerosis Society Consensus Panel. European heart journal. 2017;38(32):2459-72.
35. McDonald AM, Knight RC, Campbell MK, Entwistle VA, Grant AM, Cook JA, et al. What influences recruitment to randomised controlled trials? A review of trials funded by two UK funding agencies. Trials. 2006;7(1):9.
36. Vallejo-Vaz AJ, De Marco M, Stevens CAT, Akram A, Freiberger T, Hovingh GK, et al. Overview of the current status of familial hypercholesterolaemia care in over 60 countries - The EAS Familial Hypercholesterolaemia Studies Collaboration (FHSC). Atherosclerosis. 2018;277:234-55.
37. Broekhuizen K, van Poppel MN, Koppes LL, Kindt I, Brug J, van Mechelen W. Can multiple lifestyle behaviours be improved in people with familial hypercholesterolemia? Results of a parallel randomised controlled trial. PloS one. 2012;7(12):e50032.
38. Schwandt P, Geiß HC, Ritter MM, Üblacker C, Parhofer KG, Otto C, et al. The Prevention Education Program (PEP). A Prospective Study of the Efficacy of Family-Oriented Life Style Modification in the Reduction of Cardiovascular Risk and Disease: Design and Baseline Data. Journal of Clinical Epidemiology. 1999;52(8):791-800.
39. Treweek S, Lockhart P, Pitkethly M, Cook JA, Kjeldstrøm M, Johansen M, et al. Methods to improve recruitment to randomised controlled trials: Cochrane systematic review and meta-analysis. BMJ Open. 2013;3(2):e002360.
40. Howie EK, Straker LM. Rates of attrition, non-compliance and missingness in randomized controlled trials of child physical activity interventions using accelerometers: A brief methodological review. Journal of Science and Medicine in Sport. 2016;19(10):830-6.
41. Rowland MK, Adamson AJ, Poliakov I, Bradley J, Simpson E, Olivier P, et al. Field Testing of the Use of Intake24-An Online 24-Hour Dietary Recall System. Nutrients. 2018;10(11):1690.
42. Janssen MF, Pickard AS, Golicki D, Gudex C, Niewada M, Scalone L, et al. Measurement properties of the EQ-5D-5L compared to the EQ-5D-3L across eight patient groups: a multi-country study. Quality of Life Research. 2013;22(7):1717-27.
43. Hawe P, Shiell A, Riley T. Complex interventions: how "out of control" can a randomised controlled trial be? Bmj. 2004;328(7455):1561-3.
44. Rodriguez-Borjabad C, Malo AI, Ibarretxe D, Girona J, Heras M, Ferre R, et al. Efficacy of therapeutic lifestyle changes on lipid profiles assessed by NMR in children with familial and non-familial hypercholesterolemia. Clin Investig Arterioscler. 2020;32(2):49-58.
45. Sylvia LG, Bernstein EE, Hubbard JL, Keating L, Anderson EJ. Practical guide to measuring physical activity. Journal of the Academy of Nutrition and Dietetics. 2014;114(2):199-208.
46. Corder K, van Sluijs EMF, McMinn AM, Ekelund U, Cassidy A, Griffin SJ. Perception Versus Reality: Awareness of Physical Activity Levels of British Children. American journal of preventive medicine. 2010;38(1):1-8.
47. Fusch PIan, L.R. Are we there yet? Data saturation in qualitative research. The Qualitative Report. 2015;20(9):1408-16.
48. Cade JE, Warthon-Medina M, Albar S, Alwan NA, Ness A, Roe M, et al. DIET@NET: Best Practice Guidelines for dietary assessment in health research. BMC medicine. 2017;15(1):202.
49. Jones PJH, Shamloo M, MacKay DS, Rideout TC, Myrie SB, Plat J, et al. Progress and perspectives in plant sterol and plant stanol research. Nutrition reviews. 2018;76(10):725-46.
50. Grover A, Oberoi M, Rehan HS, Gupta LK, Yadav M. Self-reported Morisky Eight-item Medication Adherence Scale for Statins Concords with the Pill Count Method and Correlates

For peer review only

with Serum Lipid Profile Parameters and Serum HMGCoA Reductase Levels. Cureus. 2020;12(1):e6542-e.

For peer review only

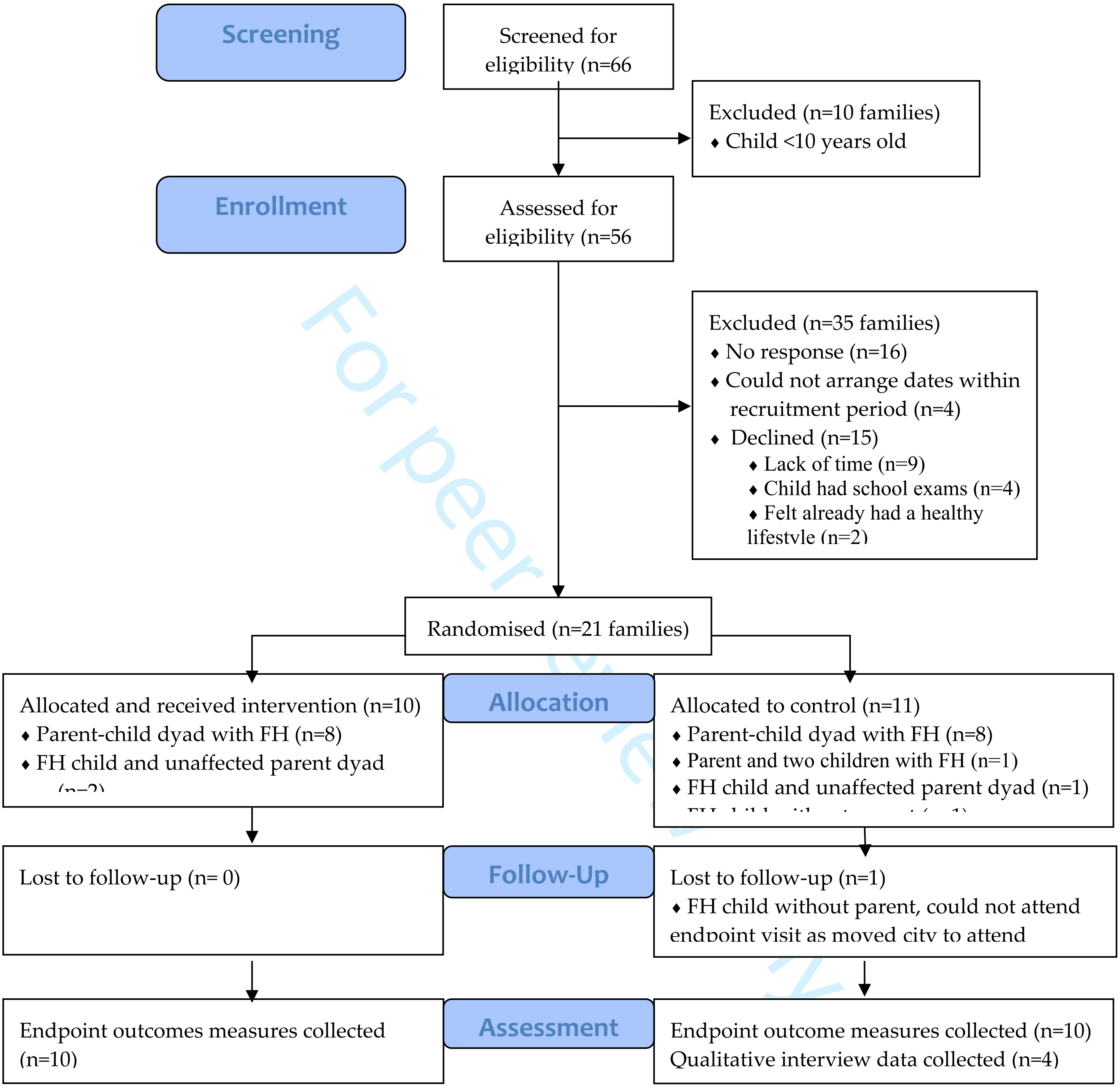


Figure 1. Consolidated Standards of Reporting Trials (CONSORT) diagram of recruitment of families across the three sites.

**Appendix 1. CONSORT 2010 checklist of information to include when reporting a pilot or feasibility trial**

| Section/Topic | Item No | Checklist item | Reported on page # |
|---|---|---|---|
| **Title and abstract** | | | |
| | 1a | Identification as a pilot or feasibility randomised trial in the title | Title |
| | 1b | Structured summary of pilot trial design, methods, results, and conclusions (for specific guidance see CONSORT abstract extension for pilot trials) | *1* |
| **Introduction** | | | |
| Background and objectives | 2a | Scientific background and explanation of rationale for future definitive trial, and reasons for randomised pilot trial | 2-3 |
| | 2b | Specific objectives or research questions for pilot trial | 3 |
| **Methods** | | | |
| Trial design | 3a | Description of pilot trial design (such as parallel, factorial) including allocation ratio | 4 |
| | 3b | Important changes to methods after pilot trial commencement (such as eligibility criteria), with reasons | n/a |
| Participants | 4a | Eligibility criteria for participants | 4 |
| | 4b | Settings and locations where the data were collected | 4 |
| | 4c | How participants were identified and consented | 4 |
| Interventions | 5 | The interventions for each group with sufficient details to allow replication, including how and when they were actually administered | 5 and Appendix 2 |
| Outcomes | 6a | Completely defined prespecified assessments or measurements to address each pilot trial objective specified in 2b, including how and when they were assessed | 5-7 |
| | 6b | Any changes to pilot trial assessments or measurements after the pilot trial commenced, with reasons | n/a |
| | 6c | If applicable, prespecified criteria used to judge whether, or how, to proceed with future definitive trial | n/a |
| Sample size | 7a | Rationale for numbers in the pilot trial | 4 |
| | 7b | When applicable, explanation of any interim analyses and stopping guidelines | n/a |
| Randomisation: | | | |
| Sequence | 8a | Method used to generate the random allocation sequence | 4 |

For peer review only

| | | | |
|---|---|---|---|
| generation | 8b | Type of randomisation(s); details of any restriction (such as blocking and block size) | 4 |
| Allocation concealment mechanism | 9 | Mechanism used to implement the random allocation sequence (such as sequentially numbered containers), describing any steps taken to conceal the sequence until interventions were assigned | 4 |
| Implementation | 10 | Who generated the random allocation sequence, who enrolled participants, and who assigned participants to interventions | 4 |
| Blinding | 11a | If done, who was blinded after assignment to interventions (for example, participants, care providers, those assessing outcomes) and how | 4 |
| | 11b | If relevant, description of the similarity of interventions | n/a |
| Statistical methods | 12 | Methods used to address each pilot trial objective whether qualitative or quantitative | 7 |
| **Results** | | | |
| Participant flow (a diagram is strongly recommended) | 13a | For each group, the numbers of participants who were approached and/or assessed for eligibility, randomly assigned, received intended treatment, and were assessed for each objective | 8 and Figure 1 |
| | 13b | For each group, losses and exclusions after randomisation, together with reasons | 8 and Figure 1 |
| Recruitment | 14a | Dates defining the periods of recruitment and follow-up | 8 |
| | 14b | Why the pilot trial ended or was stopped | 8 |
| Baseline data | 15 | A table showing baseline demographic and clinical characteristics for each group | Table 1 |
| Numbers analysed | 16 | For each objective, number of participants (denominator) included in each analysis. If relevant, these numbers should be by randomised group | Tables 2 and 3 |
| Outcomes and estimation | 17 | For each objective, results including expressions of uncertainty (such as 95% confidence interval) for any estimates. If relevant, these results should be by randomised group | Tables 2 and 3 |
| Ancillary analyses | 18 | Results of any other analyses performed that could be used to inform the future definitive trial | 8-10 |
| Harms | 19 | All important harms or unintended effects in each group (for specific guidance see CONSORT for harms) | n/a |
| | 19a | If relevant, other important unintended consequences | n/a |
| **Discussion** | | | |
| Limitations | 20 | Pilot trial limitations, addressing sources of potential bias and remaining uncertainty about feasibility | 11-13 |
| Generalisability | 21 | Generalisability (applicability) of pilot trial methods and findings to future definitive trial and other studies | 11-13 |

For peer review only

| | | | |
|---|---|---|---|
| Interpretation | 22 | Interpretation consistent with pilot trial objectives and findings, balancing potential benefits and harms, and considering other relevant evidence | 11-13 |
| | 22a | Implications for progression from pilot to future definitive trial, including any proposed amendments | 11-13 |
| **Other information** | | | |
| Registration | 23 | Registration number for pilot trial and name of trial registry | Abstract |
| Protocol | 24 | Where the pilot trial protocol can be accessed, if available | 4 |
| Funding | 25 | Sources of funding and other support (such as supply of drugs), role of funders | 15 |
| | 26 | Ethical approval or approval by research review committee, confirmed with reference number | 4 |

For peer review only

**Appendix 2. Additional details of intervention content and delivery**

Full details of the development of the intervention, and the selection of content and the delivery method, are described elsewhere.[1] This appendix will provide overview of the 1) The behavioural targets of the intervention; 2) The structure and topics covered in the intervention session; 3) Behaviour change techniques (BCTs) included and details of how they were incorporated into the sessions; 4) The BCTs actually delivered in practice to the participants during the initial and follow-up sessions in the study.

**1. Behavioural targets of the intervention**

All available clinical guidelines for the treatment of children and adults with FH include recommendations for dietary intakes and physical activity. The recommendations are very similar across guidelines, although small differences are apparent. The recommendations selected to be included as targets of the behavioural intervention in this research are summarized below:

1. Total daily fat intake ≤30% of TEI
2. Daily saturated fat intake of ≤10% TEI achieved via replacement with monounsaturated and polyunsaturated fats
3. Daily dietary cholesterol intake ≤300mg
4. Consumption of ≥ 5 portions of fruit and vegetables a day
5. Age appropriate fibre intake: 10-year-olds= 20g/day; 11-16 year-olds = 25g/day and 30g/day for ≥17 years
6. Consumption of 2g of plant sterols or stanols per day
7. Reduce time spent engaged in sedentary behaviors
8. Age appropriate physical activity levels:

- Adults aged 19-64 years: ≥ 150 minutes a week of moderate intensity physical activity or ≥ 75 minutes of vigorous intensity physical activity, or a mixture of the two. Additional activity focussing on improving muscle strength should be undertaken twice a week.
- Children aged 5-18 years: ≥ 60 minutes of moderate-to-vigorous physical activity each day, with 3 of these sessions each week being of vigorous intensity and including activities that strengthen muscle and bone.


For peer review only - http://bmjopen.bmj.com/site/about/guidelines.xhtml

These recommendations reflect the U.K. NICE guidelines,[2] as the developed intervention was to be delivered in an U.K. context, in the first instance. However, three additional recommendations were included for reasons described below:

**A) Consumption of 2g/day plant sterol or stanols**

While the U.K. NICE guidelines do not consider plant sterols and stanols to be an essential component of dietary treatment,[2] a recommendation for their intake was included in the intervention for the following reasons: the promising evidence to support the addition of plant sterols or stanols to the cholesterol lowering diet in children and adults with FH;[3] the indication for use in individuals with FH from the age of six in EAS and AHA clinical guidelines for the management of high cholesterol;[4-6] recommendation for use in other and more recent clinical guidelines for the management of FH;[7-11] their inclusion in the resources produced by *HEART UK: The cholesterol charity*,[12] which are recommended for use in the recent U.K. guidelines for the management of FH in children.[13]

**B) Age appropriate fibre intakes**

All the clinical guidelines advocate a Mediterranean dietary pattern and the high fibre content of this dietary pattern may be of particular benefit to individuals with high cholesterol levels.[14-17] Therefore a specific recommendation for fibre intake was included as a specific goal of dietary treatment in this research, using values derived from the guidelines for fiber intake for the general population in the U.K.[18]

**C) Reduce time spent engaged in sedentary behaviours**

Only the NICE guidelines specify the type or amount of physical activity to be included as part of lifestyle treatment which is based upon the advice for the general population.[2] However, the lifestyle treatment section of the NICE guidelines have not been updated since 2008 and therefore do not include reflect the updated physical activity guidelines for the general population which now include advice to reduce sedentary behaviours.[19] This was therefore added as an additional recommendation to be targeted by the intervention.


For peer review only - http://bmjopen.bmj.com/site/about/guidelines.xhtml

**D) No specific target for trans fatty acids**

While not stated in the U.K. NICE guidelines,[2] other clinical guidelines advocate reducing intake of trans fatty acids (TFA).[8,10-11] This was not selected as a recommendation to be targeted in the intervention in this research for two reasons. Firstly, intakes in the U.K. general population are below the maximum recommended intakes of 2% total energy intake.[20] Secondly, as most TFA are found in processed foods, it is recognised that reducing intakes of TFA requires interventions targeting the use of TFA by the food industry.[21-22] It was therefore not deemed to be appropriate or required for the intervention to target intakes of TFA in the present research. Lastly, in light of the lack of research to support the effectiveness of the current recommendations, it was decided that the intervention would not target additional dietary components that have been investigated to a small extent, such as protein or omega-3 fatty acids.[3]

**2. The structure of, and topics covered in, the intervention sessions**

The intervention was delivered to families by a dietitian as an initial 1-hour face-to-face session followed by four telephone or email follow-up sessions. The intervention primarily focused upon providing education about the dietary and physical activity recommendations and the importance of adhering to these in addition to pharmacological treatment for the avoidance of long-term health consequences associated with FH. The dietary and physical activity recommendations to be targeted in the intervention were communicated as six targets, which provided structure for the intervention booklets and intervention sessions. The targets were:

1) Reduce how many foods high in saturated fat you eat and replace with foods rich in unsaturated fats

2) Limit how many foods high in dietary cholesterol you eat per week

3) Increase the number of portions of fruits and vegetables you eat each day

4) Increase how many insoluble and soluble fiber-rich foods you eat each day

5) Have one plant sterol or stanol fortified yogurt drink each day

6) Increase how physically active you are and reduce time spent being sedentary

Achievable goals were set for each of the six dietary and physical activity targets, tailored towards each individuals current physical activity levels, dietary intakes and unique personal and family characteristics. These goals were SMART (specific, measurable, acceptable, realistic, time-based) in nature.[23] The dietitians provided



For peer review only - http://bmjopen.bmj.com/site/about/guidelines.xhtml

verbal and written (in intervention booklets developed specifically for this trial) instructions on how to achieve these targets such as food swaps, cooking techniques, and recipes. The goals were reviewed at each follow-up session alongside further problem solving and action planning sessions to facilitate continued attainment of goals. Parents and children were encouraged to view each other as sources of emotional and social support and other family members encouraged to adopt the lifestyle changes. Individuals will be encouraged to self-monitor their behaviours through the use of self-reflection diaries and checklists to monitor intake of foods fortified with plant sterols and stanols.

The initial session comprised of eight sections, as shown in Table 1 alongside the intended aims for participants. Each follow-up session comprised of four sections: *introduction and overview; review of goals; barriers and solutions; further support and follow up*.

3. **Behaviour change techniques (BCTs) included and details of how they were incorporated into the sessions**

Table 2 displays the BCTs to be included in the initial session and Table 3 displays the BCTs to be included in the follow-up sessions. These 'BCT checklists' were provided to dietitians to provide them with instructions on how to incorporate the BCTs into the sessions and to monitor fidelity. Descriptions of each BCT are provided in full in The BCT Taxonomy (V1).[24] Dietitians were instructed to record any adaptations they made to the intended BCTs- including if they delivered other additional BCTs.



For peer review only - http://bmjopen.bmj.com/site/about/guidelines.xhtml

Table 1. The eight sections of the initial intervention session and participant aims

| Section | Aim(s) for participants |
|---|---|
| **1. Introduction & 2. Scientific rationale** | • To understand the importance of diet and physical activity in the management of FH and for their overall health.<br>• To be aware of the importance placed on diet and physical activity by national and international guidelines for FH and the current recommendations in England that all FH patients should receive individualised advice about diet & physical activity<br>• To understand that the earlier treatment for FH starts, the more effective it is and this is why it is important to optimise nutrition and physical activity from a young age |
| **3. Education about dietary guidelines** | • To know what a healthy balanced diet looks like, including the food groups and the proportion each one should make to diet<br>• To know what the five diet targets of the intervention are<br>• To understand why each target is important for their health and for the management of their FH<br>• To understand what foods to include/exclude and/or increase/decrease consumption of to achieve targets |
| **4. Education about physical activity guidelines** | • To know what the physical activity and sedentary behaviour recommendations are for their age<br>• To know why these recommendations are important for their health and for management of their FH<br>• To know what the different levels of physical activity are (low, moderate and high) and what types of physical activity fall into which group<br>• To understand how they can incorporate more physical activity into their lifestyle to help increase levels to recommended amounts, or more in addition to reducing sedentary behaviours. |
| **5. Goal setting** | • To agree with dietitian upon SMART goals for each of the targets. These will be changes to their lifestyle that they agree to make over the following 12 weeks to achieve nutritional intakes and physical activity levels closer to the guidelines |
| **6. Barriers and solutions** | • To identify potential barriers that may prevent them from meeting the goals set<br>• To identify, through discussion with dietitian and other family member, solutions to these barriers |
| **7. Follow up arrangements & 8. Wrap-up** | • To understand what will happen over the following 12 weeks<br>• To understand the purpose of the weekly reflection diaries and know how to fill them out<br>• To know what will be discussed during the follow-up sessions<br>• An opportunity to ask any unanswered questions<br>• To receive summary of session discussion & receive encouragement and motivation from dietitian |

For peer review only

**Table 2. BCT checklist for initial session of intervention to be completed by dietitians**

| Behaviour change technique | How to be used within intervention | Included? |
|---|---|---|
| Action planning | Individual will be prompted to develop specific planning of how they will achieve each goal set i.e. *if increasing fibre intake then the food swap or additional food to be included in diet will be specified, along with what meal or snack they will include it in and how many times per day or week.* | |
| Behaviour substitution | Individuals encouraged to set goals that involve swapping something they currently do everyday with something that will help them achieve the guidelines to help establish new healthy habits i.e. *walking to and from work instead of driving* or *always having a piece of fruit when they make their morning cup of tea instead of a biscuit*. | |
| Behavioural practice/rehearsal | Prompt individual to practice cooking during intervention and adults to encourage child to help them. | |
| Biofeedback | Individual to be provided with weight, body fat % and blood pressure before starting the intervention to prompt adoption of guidelines- either to improve these figures or maintain them. | |
| Comparative imaging of future outcomes | Dietitian to prompt individual to think about what the possible health outcomes would be if they choose to follow guidelines compared to if they chose not to- with emphasis on what this would mean for their children or parents. | |
| Credible sources | • Intervention delivered by a dietitian who will explain the training they have undertaken to gain that title- to help individual recognise that their advice is credible. All individuals will also be informed that their doctor is aware and supportive of them receiving the intervention as they view it as being part of their clinical care.<br>• Individual to be receive verbal and written advice about the guidelines which will include information about where the guidelines have come from- national committees across several countries who have reviewed all the available scientific evidence and come to same conclusion about the guidelines that individuals with FH should be following. | |
| Demonstration of the behaviour | Individual to be signposted to resources which can aid with demonstration of the behaviour i.e. *step by step recipe videos or pictorial guides and parents encouraged to demonstrate cooking skills to child.* | |
| Feedback on outcomes of behaviour | Individual to receive feedback about how their dietary intakes and physical activity levels compare to the guidelines at the start and end of the intervention as evidence of their capability of adhering to the guidelines to promote maintenance of behaviours after intervention ended.<br>Note- the follow-up information will be delivered at research contact 3. Provide initial information in initial session. | |
| Framing/re-framing | • Guidelines communicated to individual as being specifically for individuals with FH as opposed to general healthy lifestyle guidelines provided to all individuals. The inclusion of two specific dietary guidelines for individuals with FH such as eating foods fortified with plant stanols/sterols and reducing dietary cholesterol intakes will help individuals to buy into the idea that following the guidelines is part of their identity of having FH. The benefits of following the guidelines in the management of their FH will be emphasised, in addition to general overall health benefits.<br>• Dietary guidelines to be communicated as a healthy lifestyle rather than a restrictive diet, with all foods permitted. Emphasis will be put on foods to add into the diet (fruits, vegetables, fibre rich foods, plant sterols/stanols) to help individual view dietary choices are positive and enjoyable.<br>• Physical activity to be communicated positively, with emphasis placed upon finding activities that the individual enjoys doing, either alone or with friends or family, rather than it being a chore they have to try and fit into their day without enjoying it.<br>• Individual prompted to view the guidelines as behaviours that can help reduce their risk of developing symptoms as their family members have, or reduce the likelihood of experiencing further symptoms. They are something the individual can do to help take control of their health. | |
| Goal setting (behavioural) | • Individual prompted to set their own goals which they feel are achievable-which take into consideration their food preferences, fitness levels, readiness to change and lifestyles. The goals will be SMART. Parents will be encouraged to help child with setting their goals. | |

| | | |
|---|---|---|
| Graded tasks | • Child to be prompted to help with food preparation, starting with simple task such as preparing vegetables, progressing to more complex tasks until able to prepare full meal.<br>• Adults to be prompted to start cooking meals- starting with simple recipes and progressing to more complex ones. | |
| Identification of self as a role model | • Parents encouraged to view themselves as role models for their children and make their behaviours part of everyday life for their children to help them foster adoption of healthy habits from a young age. Dietitian to communicate the benefits engaging in the behaviours could bring to these significant others.<br>• Children also encouraged to view themselves as role models for their parents, other siblings and friends. | |
| Information about antecedents | Individual prompted to consider specific contexts in which they find it hard to adhere to dietary guidelines and identify triggers for this behaviour. The findings will be addressed in action planning and problem-solving session of the intervention. | |
| Information about emotional consequences | • Dietitian to discuss with individual the potential improvement in their mood that they could experience if they choose to engage in dietary behaviours.<br>• Dietitian to emphasis to individual that if they find foods that they enjoy that fit the guidelines, they will also experience the same enjoyment of these foods. | |
| Information about health consequences | • The intervention starts with explanation from dietitian about the importance of lifestyle guidelines in the management of FH. It will be explained that despite use of medication, many individuals with FH may still be at higher risk of cardiovascular disease and adherence to lifestyle guidelines can help reduce this risk. The benefits to their overall health will also be communicated.<br>• Individuals to be informed about the 'silent' nature of cholesterol and the importance of keeping cholesterol low for your whole life, before any symptoms occur. | |
| Instruction on how to perform behaviour | • Individuals will receive detailed instructions about food swaps and cooking methods. The advice given will provide instructions to individual about how they can achieve the desired behaviours in their current *context i.e. cheaper options for suggested food swaps, suggested food swaps one they can obtain in the current place they shop, easy and quick options for meals if individual has limited time to cook and suggestions of physical activity they can fit into their current routines such as walking instead of bus/car.*<br>• The dietary intake data will be analysed ahead of intervention to establish any existing eating habits that are indicative of misinformation i.e. *cooking with coconut oil.* | |
| Problem solving | During intervention 'barriers and solutions' section, dietitian will encourage individual to think of situations in which they feel they will struggle to engage in the desired behaviours (*i.e. social situations*) and think of solutions to overcome these. | |
| Prompts and cues | • Individual advised to leave intervention booklet in a place they regularly eat to prompt them to engage in the dietary goals.<br>• Set reminders on phones/fitness monitors to move regularly. | |
| Restructuring the physical environment | • Advise individual to keep food choices they enjoy eating, that are in line with the guidelines, in the house/at work/in car to encourage consumption of these<br>• Individuals encouraged to socialise with friends and family in places that facilitate engagement of desired behaviours i.*e. meet in the park* or *choose restaurants in which there are suitable options for them.* | |
| Restructuring the social environment | The intervention is delivered at a family-based level with parent and child making dietary and physical activity choices together. Any other family members will also be encouraged to make the changes as well to facilitate a home environment that encourages adherence to the dietary and physical activity guidelines. | |
| Social support (emotional) | • Dietitian to provide emotional support during intervention- discussing with them what else is going on in their life and how this is influencing their ability to adhere to guidelines.<br>• Individual also encouraged to seek support from the family member(s) they are taking part in the intervention with- to view it as a 'team effort' and provide support and encouragement to each other.<br>• Dietitian to encourage parent and child to provide support to each other throughout the intervention and encourage each other to engage in the guidelines. | |

| | | |
|---|---|---|
| | • Individuals also encouraged to seek the emotional support of friends and family. | |
| Social support (practical) | • Dietitian to provide practical support during the session- helping individual to identify methods to help encourage the behaviour *i.e. online recipes or suggestions on how they could obtain practical support from friends* or *having their parent prepare help make their lunch.*<br>• The dietitian will encourage the whole family (both with and without FH) to engage in the dietary and physical activity guidelines to provide support to the individuals.<br>• Individuals encouraged to seek support from family members (including those not taking part in intervention) and friends i.*e. arranging to go to the gym with a friend or having partner/sibling help with food shopping or meal preparation.* | |
| Verbal persuasion about capabilities | Dietitian to encourage individual and tell them that they are capable of changing their behaviours. | |
| Self-monitoring of behaviour (prompt) | Individuals asked to complete weekly reflection diaries in which they record whether they have been able to meet the goals set for lifestyle behaviours.<br>They will also be asked to keep checklists of plant stanol/sterol drink consumption. | |

**Table 3**. **BCT checklist for intervention follow-up sessions completed by dietitians**

| Behaviour change technique | Instructions for use within intervention | Included? |
|---|---|---|
| Action planning | • Individual will be prompted to develop specific planning of how they will continue to, or start to, achieve each goal set at previous and current session i.e. if increasing fibre intake then the food swap or additional food to be included in diet will be specified, along with what meal or snack they will include it in and how many times per day or week.<br>• Follow up sessions are opportunity to review action plans set a previous session and identify what has worked and what needs more planning. | |
| Problem solving | • During 'barriers and solutions' section, dietitian will review with individual the barriers and solutions they discussed at previous session. Together they will identify what they will do next if barrier still preventing them from achieving goal.<br>• Dietitian will encourage individual to think of other situations in which they feel they will struggle to engage in the desired behaviours between now and next follow up session and think of solutions to overcome these. | |
| Social support (emotional) | • Dietitian to provide emotional support during follow-up sessions- discussing with them what else is going on in their life and how this is influencing their ability to adhere to guidelines.<br>• Individuals encouraged to seek the emotional support of friends and family<br>• Individual encouraged to seek support from the family member(s) they are taking part in the intervention with- to view it as a 'team effort' and provide support and encouragement to each other to engage in the guidelines | |
| Social support (practical) | • Dietitian provide practical support during follow-up sessions- helping individual to identify methods to help encourage the behaviour i.e. links to online resources or suggestions on how they could obtain practical support from friends or family i.e. having their parent prepare help make their lunch<br>• Individuals encouraged to seek support from family members (including those not taking part in intervention) and friends i.e. arranging to go | |



For peer review only - http://bmjopen.bmj.com/site/about/guidelines.xhtml

| | | |
|---|---|---|
| | to the gym with a friend or having partner/sibling help with food shopping or meal preparation. | |
| Verbal persuasion about capabilities | Dietitian to encourage individual and tell them that they are capable of changing their behaviours. | |
| Focus on past success | Dietitian to focus on any successful changes individuals have made and use these to provide encouragement and motivation to individual to carry on and make more changes. | |
| Review behavioural goal(s) | At each follow-up session, dietitian and individual will review the goals set at previous session or follow-up. Together they will agree to either: keep goal the same, modify the goal or create new goal. These decisions will be based upon individuals levels of achievement and willingness to change. | |
| Self-monitoring of behaviour (prompt to) | Individuals asked to complete weekly reflection diaries in which they record whether they have been able to meet the goals set for lifestyle behaviours which will be discussed during follow-ups. They will also be asked to keep checklists of plant stanol drink consumption which will be reviewed at each follow up session. | |
| Self-monitoring of behaviour (as reported by individuals) | Individuals self-reported completion of the weekly self-reflection diaries and plant stanol checklist. | |

For peer review only - http://bmjopen.bmj.com/site/about/guidelines.xhtml

4. The BCTs actually delivered in practice to the participants during the initial and follow-up sessions.

The BCTs intended to have been delivered in the initial and follow up sessions are indicated with a tick in Table 4 alongside the percentage of families who received each one in practice. Further details of how each BCT was to be delivered in the intervention are displayed in Table 2 and Table 3. Overall, the fidelity was high with the majority of BCTs delivered as intended across all sessions and sites. Four BCTs (*demonstration of the behaviour*; *graded tasks*, *problem solving* and *self-monitoring of behaviour prompt)* were not delivered to all families. In all instances, this was reported by the dietitians to be due to it not being applicable to the individual family. For instance, the dietitian reported it was not appropriate to prompt families, who had previously stated they were not, or did not want to, use the self-reflection resources at previous follow-up sessions. The families stated they did not have time or did not feel it was necessary to help them achieve their goals. Two further BCTs *biofeedback* and *feedback on outcomes of behaviour* were not delivered to families (n=2) at the London study site as the dietitian did not have access to the dietary intake or anthropometric data ahead of the initial session.

In response to the needs of each individual family, the dietitians reported the occasional use of additional BCTs during the follow-up sessions, as indicated by the plus sign in Table 4. The dietitians reported using these BCTs to help families overcome barriers they were facing in achieving their goals. For instance, *instruction on how to perform the behaviour* was delivered to 20% of families during follow-up one who had requested recipes or cooking advice.

For peer review only

Table 4. The BCTs intended to be included in the initial and follow-up sessions (✓), the additional BCTs delivered (+) and the percentage of families who received each BCT in each session (%)

| Behaviour change technique (BCT)* † | Initial session (n=10) | Follow-up 1 (n=10) | Follow-up 2 (n=10) | Follow-up 3 (n=10) | Follow-up 4 (n=10) |
|---|---|---|---|---|---|
| **Action planning** | ✓ (100%) | ✓ (100%) | ✓ (100%) | ✓ (100%) | ✓ (100%) |
| **Behaviour substitution** | ✓ (100%) | + (20%) | + (10%) | + (5%) | |
| **Behavioural practice/rehearsal** | ✓ (100%) | | | | |
| **Biofeedback** | ✓ (80%) | | | | |
| **Comparative imaging of future outcomes** | ✓ (100%) | | | | |
| **Credible sources** | ✓ (100%) | | | | |
| **Demonstration of the behaviour** | ✓ (0%) | + (5%) | | | |
| **Feedback on outcomes of behaviour** | ✓ (90%) | | | | |
| **Framing/re-framing** | ✓ (100%) | + (25%) | + (25%) | + (5%) | |
| **Goal setting (behavioural)** | ✓ (100%) | | | | |
| **Graded tasks** | ✓ (30%) | | | | |
| **Identification of self as a role model** | ✓ (100%) | | | | |
| **Information about antecedents** | ✓ (100%) | | | | |
| **Information about emotional consequences** | ✓ (100%) | | | | |
| **Information about health consequences** | ✓ (100%) | | | | |
| **Instruction on how to perform behaviour** | ✓ (100%) | + (20%) | + (10%) | + (11%) | |
| **Problem solving** | ✓ (100%) | ✓ (100%) | ✓ (100%) | ✓ (84%) | ✓ (100%) |
| **Prompts and cues** | ✓ (100%) | | + (5%) | + (5%) | |
| **Restructuring the physical environment** | ✓ (100%) | + (10%) | | | |
| **Restructuring the social environment** | ✓ (100%) | | | | |
| **Social support (emotional)** | ✓ (100%) | ✓ (100%) | ✓ (100%) | ✓ (100%) | ✓ (100%) |
| **Social support (practical)** | ✓ (100%) | ✓ (100%) | ✓ (100%) | ✓ (100%) | ✓ (100%) |
| **Verbal persuasion about capabilities** | ✓ (100%) | ✓ (100%) | ✓ (100%) | ✓ (100%) | ✓ (100%) |
| **Focus on past success** | | ✓ (100%) | ✓ (100%) | ✓ (100%) | ✓ (100%) |

For peer review only

| | | | | | |
|---|---|---|---|---|---|
| **Review behavioural goal(s)** | | ✔ (100%) | ✔ (100%) | ✔ (100%) | ✔ (100%) |
| **Self-monitoring of behaviour (prompt to)** | ✔ (90%) | ✔ (100%) | ✔ (100%) | ✔ (68%) | ✔ (50%) |

*Details of how each BCT was delivered in the intervention are available in Table 2 and Table 3.
† Further details of the BCTs are available on the BCT taxonomy (BCTTv1)[24]

For peer review only

References

1.Kinnear FJ, Wainwright E, Bourne JE, Lithander FE, Hamilton-Shield J, Searle A. The development of a theory informed behaviour change intervention to improve adherence to dietary and physical activity treatment guidelines in individuals with familial hypercholesterolaemia (FH). BMC health services research. 2020;20(1):27.
2.NICE clinical guideline 71: Familial hypercholesterolemia: identification and management. [Internet]. 2008 [cited 19/01/2018]. Available from: http://nice.org.uk/guidance/cg71.
3.Malhotra A, Shafiq N, Arora A, Singh M, Kumar R, Malhotra S. Dietary interventions (plant sterols, stanols, omega-3 fatty acids, soy protein and dietary fibers) for familial hypercholesterolaemia. Cochrane Database Syst Rev. 2014(6):CD001918.
4.Gylling H, Plat J, Turley S, Ginsberg HN, Ellegard L, Jessup W, et al. Plant sterols and plant stanols in the management of dyslipidaemia and prevention of cardiovascular disease. Atherosclerosis. 2014;232(2):346-60.
5.Mach F, Baigent C, Catapano AL, Koskinas KC, Casula M, Badimon L, et al. 2019 ESC/EAS Guidelines for the management of dyslipidaemias: lipid modification to reduce cardiovascular risk: The Task Force for the management of dyslipidaemias of the European Society of Cardiology (ESC) and European Atherosclerosis Society (EAS). European heart journal. 2019;41(1):111-88.
6.Grundy Scott M, Stone Neil J, Bailey Alison L, Beam C, Birtcher Kim K, Blumenthal Roger S, et al. 2018 AHA/ACC/AACVPR/AAPA/ABC/ACPM/ADA/AGS/APhA/ASPC/NLA/PCNA Guideline on the Management of Blood Cholesterol. Circulation.0(0):CIR.0000000000000625.
7.Goldberg AC, Hopkins PN, Toth PP, Ballantyne CM, Rader DJ, Robinson JG, et al. Familial hypercholesterolemia: screening, diagnosis and management of pediatric and adult patients: clinical guidance from the National Lipid Association Expert Panel on Familial Hypercholesterolemia. J Clin Lipidol. 2011;5(3 Suppl):S1-8.
8.Nordestgaard BG, Chapman MJ, Humphries SE, Ginsberg HN, Masana L, Descamps OS, et al. Familial hypercholesterolaemia is underdiagnosed and undertreated in the general population: guidance for clinicians to prevent coronary heart disease : Consensus Statement of the European Atherosclerosis Society. European heart journal. 2013;34(45):3478-90.
9.Wiegman A, Gidding SS, Watts GF, Chapman MJ, Ginsberg HN, Cuchel M, et al. Familial hypercholesterolaemia in children and adolescents: gaining decades of life by optimizing detection and treatment. European heart journal. 2015;36(36):2425-37.
10.Watts GF, Gidding S, Wierzbicki AS, Toth PP, Alonso R, Brown WV, et al. Integrated guidance on the care of familial hypercholesterolaemia from the International FH Foundation. International journal of cardiology. 2014;171(3):309-25.
11.Gidding SS, Champagne MA, de Ferranti SD, Defesche J, Ito MK, Knowles JW, et al. The Agenda for Familial Hypercholesterolemia: A Scientific Statement From the American Heart Association. Circulation. 2015;132(22):2167-92.
12.HEART UK: The Cholesterol Charity. Familial hypercholesterolaemia: An education booklet for people with familial hypercholesterolaemia (FH). London HEART UK: The cholesterol charity; 2019.
13.Ramaswami U, Humphries SE, Priestley-Barnham L, Green P, Wald DS, Capps N, et al. Current management of children and young people with heterozygous familial hypercholesterolaemia - HEART UK statement of care. Atherosclerosis. 2019;290:1-8.

For peer review only

14.McRae MP. Dietary Fiber Is Beneficial for the Prevention of Cardiovascular Disease: An Umbrella Review of Meta-analyses. Journal of Chiropractic Medicine. 2017;16(4):289-99.
15.Brown L, Rosner B, Willett WW, Sacks FM. Cholesterol-lowering effects of dietary fiber: a meta-analysis. The American journal of clinical nutrition. 1999;69(1):30-42.
16.Whitehead A, Beck EJ, Tosh S, Wolever TM. Cholesterol-lowering effects of oat β-glucan: a meta-analysis of randomized controlled trials. The American journal of clinical nutrition. 2014;100(6):1413-21.
17.Ho HV, Sievenpiper JL, Zurbau A, Blanco Mejia S, Jovanovski E, Au-Yeung F, et al. The effect of oat β-glucan on LDL-cholesterol, non-HDL-cholesterol and apoB for CVD risk reduction: a systematic review and meta-analysis of randomised-controlled trials. Br J Nutr. 2016;116(8):1369-82.
18.Nutrition SACo. Carbohydrates and Health. London: The Stationery Office (TSO); 2015.
19.Department of Health and Social Care. UK Chief Medical Ofiicers' Physical Activity Guidelines London: UK Government Department of Health and Social Care; 2019.
20.Public Health England. National Diet and Nutrition Survey Results from Years 7 and 8 (combined) of the Rolling Programme (2014/2015 to 2015/2016). London: Public Health England and Food Standards Agency 2018.
21.Hyseni L, Bromley H, Kypridemos C, O'Flaherty M, Lloyd-Williams F, Guzman-Castillo M, et al. Systematic review of dietary trans-fat reduction interventions. Bulletin of the World Health Organization. 2017;95(12):821-30G.
22.Hendry VL, Almíron-Roig E, Monsivais P, Jebb SA, Neelon SE, Griffin SJ, et al. Impact of regulatory interventions to reduce intake of artificial trans-fatty acids: a systematic review. American journal of public health. 2015;105(3):e32-42.
23.Ogbeiwi O. Why written objectives need to be really SMART. British Journal of Healthcare Management. 2017;23(7):324-36.
24.Michie S, Richardson M, Johnston M, Abraham C, Francis J, Hardeman W, et al. The behavior change technique taxonomy (v1) of 93 hierarchically clustered techniques: building an international consensus for the reporting of behavior change interventions. Ann Behav Med. 2013;46(1):81-95.

For peer review only

**Appendix 3 Additional details of outcome measures used in study**

*Behavioural outcomes (study objectives 5 & 6)*

Behavioural outcomes were collected in the week preceding research contact two (baseline) and research contact three (endpoint).

1. Dietary intakes: participants recorded their dietary intake on four non-consecutive days (including one weekend day) using Intake24, a validated online 24-hour recall tool.[1,2] Data were downloaded from Intake24 as an Excel file before being cleaned and analysed, in line with Intake24 data processing guidelines. After data quality checks, data not meeting the validity criteria (completion rate <2 minutes; daily energy intake <2000KJ or >16000KJ) were excluded and only participants with two weekdays and one weekend day of valid intake were included in the analysis. Using pivot tables, daily intakes of macronutrients were calculated. Intakes of fruits and vegetables were presented as mean portions consumed per day by dividing the sum intake of fruit and vegetables by 80, per participant and per day. The value of 80 was chosen as a single portion of fruit and vegetables, in U.K. national guidelines, is considered to be 80g. Dried fruit, fruit juices and pulses were included but a portion was considered to be 30g, 150ml and 80g respectively, and fruit juices and pulses were capped at one portion per day. Fruits and vegetables in composite dishes were not included as there is not yet a validated method to calculate this using the Intake24 database. In line with the specific dietary goals of the intervention described in appendix 2, mean daily intakes of fat, saturated fat, monounsaturated fats, polyunsaturated fats, cholesterol, fiber, fruit and vegetable portions, and plant sterol or stanol fortified foods were chosen as outcome measures.

2. Physical activity levels: average daily minutes of free-living moderate and vigorous physical activity (MVPA) was measured by asking participants to wear an ActiGraph GT3X+ accelerometer monitor (ActiGraph, Pensacola, USA) during waking hours for seven consecutive days. Participants also completed logbooks to capture waking/sleeping times and any activities during which the monitor was taken off. Raw data were downloaded and analysed, in 15 and 60-second epochs for children and adults respectively, using the manufacturer's software (ActiLife software v6.13.4; ActiGraph, Pansacola, FL, USA). Logbook data and an algorithm, which detected non-wear as periods of 60 minutes or more of consecutive zeros with no allowance


For peer review only - http://bmjopen.bmj.com/site/about/guidelines.xhtml

for interruptions, was used to identify valid weartime periods. A second researcher (AET) analysed the data from 10 participants (five adults and five children) to ensure consistency and validity of the decisions made by FJK. To determine MVPA, the cut-points developed by Freedson[3] and Evenson[4] were used for adults and children, respectively. At least four valid days (defined as ≥ 8 hours weartime), including at least one weekend day, were required for analysis.

3. Sedentary time: participants wore an activPAL3 accelerometer (PAL Technologies Ltd., Glasgow, UK) for seven consecutive days to estimate the average daily time spent sedentary (sitting or lying).[5] Accelerometers were made waterproof using a nitrile sleeve and were attached to the front of the thigh using a waterproof hypoallergenic medical dressing (Hypafix), enabling participants to wear the monitor continuously for 24 hours/day, removing only for swimming. Data was downloaded using the manufacturer's software (PAL software v7.2.38; PAL Technologies Ltd., Glasgow, UK) and was cleaned and processed in ProcessingPAL, a Java application which utilises a validated algorithm to isolate waking weartime.[6] Logbooks were used to aid identification of the non-wear periods and sleep time, which were removed from analysis. A second researcher (AET) analysed the data from 10 participants (five adults and five children) to ensure consistency and validity of the decisions made by FJK. At least four valid days (defined as ≥10 h of worn waking hours, <95% of time spent in any one position and ≥500 steps), including one weekend day was required for analysis.



For peer review only - http://bmjopen.bmj.com/site/about/guidelines.xhtml

Table 1. Data processing and analysis decisions for data collected using Actigraph accelerometers

| Factor to consider | Description | Approach taken in this study: *Adults* | Approach taken in this study: *Children* |
|---|---|---|---|
| **EPOC length** | EPOC length is the time period over which the ActiGraph counts are totalled and can range between 1 and 60 seconds. | • 60 second epochs | • 15 second epochs |
| **Cut-points for classification of activity intensity** | To interpret the counts per epoch, thresholds are applied to define different intensities of PA known as cut-points. There are several sets of cut-points available. | • Freedson et al[3] | • Evenson et al[4] |
| **Classification of non-wear time** | Periods of time that participant is not wearing Actigraph should be detected and deleted from the data to avoid misclassifying non-wear time as sedentary time. There are a variety of available algorithms to detect non-wear time, which differ in the criteria they use. | • An algorithm which detects non-wear as periods of 60 minutes or more of consecutive zeros with no allowance for interruptions<br>• Participants are asked to complete logbooks to capture non-wear occasions and reasons, to cross check results from algorithm<br>• Self-reported PA during non-wear times (e.g. swimming or contact sports) were not included in analysis | |
| **Valid day & minimum number of days criteria** | The number of hours of weartime required to define a valid day, and the number of these valid days required for data to be included in the analysis. | • A valid day is defined as ≥ 8 hours (480 minutes)<br>• Four valid days (including one weekend day) were required for inclusion in analysis | |
| **Uni- or tri-axial counts** | The ActiGraph detects activity counts across three axes (vertical, mediolateral and anteroposterior). Researchers can choose to use the vector magnitude (VM) score, which includes activity counts detected across all axes, or use activity counts detected across vertical axis only. | • Activity counts detected across vertical axis were analysed to match the approach used in the studies that developed the MVPA cut-points | |



For peer review only - http://bmjopen.bmj.com/site/about/guidelines.xhtml

*Clinical outcomes (Study objectives 5 and 6)*

Clinical outcomes were collected from participants at research contact two (baseline) and three (endpoint) by members of the research team.

1. Anthropometry and body composition: Height was measured using a stadiometer and weight, body fat percentage and fat free mass using bioelectrical impedance scales (Tanita™ MC-780MA). At sites where this equipment was unavailable, weight was measured using medical scales. These outcomes were used to calculate the body mass index (BMI) for adult participants. The LMS method was used to express the BMIs of child participants as BMI Z-scores and percentiles.[7] This was carried out using the lmsGrowth excel add-in[8] and the British 1990 reference population.[9] The 91$^{st}$ and 98$^{th}$ BMI percentiles were applied to define overweight and obesity as recommended for clinical interventions.[10]

2. Resting arterial blood pressure: The mean of two sphygmomanometer readings (three if 1$^{st}$ and 2$^{nd}$ differed by more than 10mmHg) was calculated to provide systolic and diastolic blood pressure outcomes. Participants were seated during the measurements, which were taken after a period of at least five minute of rest to allow values to return to resting levels.

3. HRQoL: Children completed an age appropriate Pediatric Quality of Life Inventory ™ (PedsQL™) Version 4.0 and adults completed an EuroQol Group EQ-5D-3L health questionnaire.[11,12] For children, the completed inventories were scored using the recommended scoring system[13] to produce a score for HRQoL and a breakdown of the component physical and psychosocial (emotional, social and school) functioning scores. For adults, the recommended scoring system was used[12] to produce a five-digit health state profile representative of five dimensions of health which was converted into a single index value using a validated value set created for use in the United Kingdom.[14] The visual analogue scale data was presented as a single value between 1 and 100.

4. Blood lipid profile: 25ml fasted blood samples were collected and a proportion was analysed immediately in the local NHS laboratory. Analysis was done by routine homogeneous enzymatic methods using Cobas reagents (Cholesterol Gen 2;



For peer review only - http://bmjopen.bmj.com/site/about/guidelines.xhtml

Triglyceride Gen 1; HDL Gen 3 or 4) on Roche/Hitachi c701/2 analysers. LDL-C concentration was then calculated using the Friedewald equation.[15] The remaining sample was processed and stored at -80°C as plasma or serum. Subsequent batch metabolomics analysis to determine 201 metabolomics is planned, but not presented in this manuscript due to laboratory closure during the COVID-19 outbreak.

## References

1.Simpson E, Bradley J, Poliakov I, Jackson D, Olivier P, Adamson AJ, et al. Iterative Development of an Online Dietary Recall Tool: INTAKE24. Nutrients. 2017;9(2):118.

2.Bradley J, Simpson E, Poliakov I, Matthews J, Olivier P, Adamson A, et al. Comparison of INTAKE24 (an Online 24-h Dietary Recall Tool) with Interviewer-Led 24-h Recall in 11–24 Year-Old. Nutrients. 2016;8(6):358.

3.Freedson PS, Melanson E, Sirard J. Calibration of the Computer Science and Applications, Inc. accelerometer. Medicine & Science in Sports & Exercise. 1998;30(5):777-81.

4.Evenson KR, Catellier DJ, Gill K, Ondrak KS, McMurray RG. Calibration of two objective measures of physical activity for children. J Sports Sci. 2008;26(14):1557-65.

5.Atkin AJ, Gorely T, Clemes SA, Yates T, Edwardson C, Brage S, et al. Methods of Measurement in epidemiology: Sedentary Behaviour. International Journal of Epidemiology. 2012;41(5):1460-71.

6.Winkler EA, Bodicoat DH, Healy GN, Bakrania K, Yates T, Owen N, et al. Identifying adults' valid waking wear time by automated estimation in activPAL data collected with a 24 h wear protocol. Physiological measurement. 2016;37(10):1653-68.

7.Green PJ, Cole TJ. Smoothing reference centile curves: the LMS method and penalized likelihood. Statistics in Medicine. 1992;11:1305-19.

8.Pan HaC, T User's guide to lmsGrowth. London, U.K. : Medical Research Council; 2012.

9.Cole TJ, Freeman JV, Preece MA. Body mass index reference curves for the UK, 1990. Archives of Disease in Childhood. 1995;73(1):25.

10.Scientific Advisory Committee on Nutrition (SACN). SACN/RCPCH defining child underweight, overweight and obesity in the UK In: England PH, editor. London: Public Health England; 2012.

11.Rabin R, de Charro F. EQ-5D: a measure of health status from the EuroQol Group. Ann Med. 2001;33(5):337-43.

12.EuroQoL Research Foundation. EQ-5D-3L User Guide Basic information on how to use the EQ-5D-3L instrument. Version 6.0. . 2018.

13.Varni JW. Scaling and scoring of the Pediatric Quality of Life Inventory ™ PedsQL ™. 2017 [Available from: http://www.pedsql.org/PedsQL-Scoring.pdf.

14.Dolan P. Modeling valuations for EuroQol health states. Medical care. 1997;35(11):1095-108.

15.Friedewald WT, Levy RI, Fredrickson DS. Estimation of the concentration of low-density lipoprotein cholesterol in plasma, without use of the preparative ultracentrifuge. Clin Chem. 1972;18(6):499-502

For peer review only

Kinnear *et al. Pilot and Feasibility Studies* (2020) 6:42
https://doi.org/10.1186/s40814-020-00584-3

Pilot and Feasibility Studies

STUDY PROTOCOL Open Access

# Nutrition and physical activity intervention for families with familial hypercholesterolaemia: protocol for a pilot randomised controlled feasibility study

Fiona J. Kinnear[1*], Julian P. Hamilton-Shield[1], David J. Stensel[2,3], Graham Bayly[4], Aidan Searle[1], Alice E. Thackray[2,3] and Fiona E. Lithander[1]

## Abstract

**Background:** Untreated heterozygous familial hypercholesterolaemia (FH) causes high low-density lipoprotein cholesterol (LDL-C) levels and increased cardiovascular disease (CVD) risk. Despite pharmacological treatment, many treated individuals remain at higher CVD risk than non-affected individuals. This may be due to LDL-C targets not being met and presence of other CVD risk factors. Adhering to dietary and physical activity (PA) recommendations developed for individuals with FH may further reduce CVD risk. However, there is insufficient research to support the efficacy of adhering to these guidelines on LDL-C and other CVD risk factors. The need for studies to investigate the effectiveness of nutrition and PA interventions in the FH population has been widely recognised and recommended. This paper describes the protocol of a pilot, randomised controlled trial designed to evaluate the feasibility and acceptability of a specifically developed nutrition and PA intervention aimed at improving the dietary intakes and PA levels of families with FH.

**Methods:** A two-arm randomised waitlist-controlled pilot trial will be conducted across three National Health Service (NHS) sites in England, UK. Twenty-four young people with FH, aged 10–18 years, and their affected parent, will be recruited and randomly assigned to the intervention or waitlist and usual care control. The primary aim is to provide evidence for the feasibility and acceptability of delivering the intervention, explored quantitatively (rates of recruitment, retention and outcome measure completeness) and qualitatively (qualitative interviews). The secondary aim is to provide evidence for the potential efficacy of the intervention on dietary intake, PA, sedentary time, body composition, CVD risk factors and quality of life determined at baseline and endpoint assessments. The intervention will involve an hour-long consultation with a dietitian at baseline and four follow-up contacts across the 12-week intervention. It has been specifically developed for use with individuals with FH and incorporates behavioural change techniques to target identified enablers and barriers to adherence in this population.

(Continued on next page)

* Correspondence: Fiona.kinnear@bristol.ac.uk
[1]NIHR Bristol Biomedical Research Centre (Nutrition Theme), University Hospitals Bristol NHS Foundation Trust and the University of Bristol, Bristol, UK
Full list of author information is available at the end of the article

© The Author(s). 2020 **Open Access** This article is licensed under a Creative Commons Attribution 4.0 International License, which permits use, sharing, adaptation, distribution and reproduction in any medium or format, as long as you give appropriate credit to the original author(s) and the source, provide a link to the Creative Commons licence, and indicate if changes were made. The images or other third party material in this article are included in the article's Creative Commons licence, unless indicated otherwise in a credit line to the material. If material is not included in the article's Creative Commons licence and your intended use is not permitted by statutory regulation or exceeds the permitted use, you will need to obtain permission directly from the copyright holder. To view a copy of this licence, visit http://creativecommons.org/licenses/by/4.0/. The Creative Commons Public Domain Dedication waiver (http://creativecommons.org/publicdomain/zero/1.0/) applies to the data made available in this article, unless otherwise stated in a credit line to the data.

For peer review only - http://bmjopen.bmj.com/site/about/guidelines.xhtml

(Continued from previous page)

**Discussion:** This trial will estimate the feasibility and acceptability of the nutrition and PA intervention delivered to young people and parents with FH. If appropriate, this study can be used to inform the design of an adequately powered definitive trial.

**Trial registration:** ISRCTN, ISRCTN24880714. Registered 07/06/2018, http://www.isrctn.com/ISRCTN24880714.

**Keywords:** Familial hypercholesterolaemia, Diet, Physical activity, Cardiovascular disease, Intervention, Dietetics, Paediatrics, Primary prevention, Qualitative, Feasibility

## Background

Heterozygous familial hypercholesterolaemia (FH) is an autosomal dominant hereditary disorder, characterised by markedly elevated levels of low-density lipoprotein cholesterol (LDL-C) from birth [1]. The lifelong exposure to elevated LDL-C levels confers a substantially increased risk of premature cardiovascular disease (CVD) and associated mortality [2] to the 1 in 250 people affected worldwide [3]. While the introduction of pharmacological treatment has dramatically reduced the incidence of CVD in individuals with FH, they remain at increased risk [4–7]. The residual CVD risk may be attributed to the large number of adults and young people that do not reach LDL-C treatment targets, even on maximally tolerated doses [8, 9]. Additionally, the presence of other risk factors such as hypertension, obesity and type II diabetes have been shown to be independently associated with their CVD risk [6, 10–14]. Efforts are currently focussed on improving the low overall detection of this disorder, which is estimated to be identifying only 1–15% of those at risk worldwide [15]. It is therefore crucial to optimise the treatment provided to this rapidly increasing vulnerable patient population group.

Lifestyle advice is considered an important adjuvant to pharmacological treatment for individuals with FH. All individuals are recommended to receive individualised advice about diet, physical activity and the maintenance of a healthy weight from a healthcare professional (HCP) with specific expertise [16–18]. Dietary and physical activity recommendations are based on the same principles as advised for the general population, with additional emphasis to reduce intakes of foods high in fat, saturated fat and cholesterol and to consume foods with LDL-C-lowering effects such as plant phytosterols/phytostanol s[16–18].. Despite these recommendations, comprehensive systematic reviews have concluded that there is insufficient data to make any conclusions about the effectiveness of any dietary or physical activity intervention upon CVD risk or surrogate outcomes in adults or young people with FH [19, 20]. Extrapolating the results of such interventions carried out in other patient populations is inappropriate as this does not allow for consideration of the unique physiological and psychological factors present in individuals with FH. Randomised controlled trials (RCTs) to investigate the effectiveness of dietary and physical activity interventions within FH cohorts have been recommended to address this research gap [18–21].

An understanding of how and why individuals with FH display certain behaviours and an awareness of the factors influencing their decisions to adhere to treatment should be considered in the development of an intervention. Adherence to lifestyle recommendations in affected individuals is sub-optimal whereby less than 50% of adults reported following the recommended lifestyle advice [22]. Additionally, overweight and obesity has been reported in cohorts of adults and young people with FH, suggesting that the advice is not being followed by all [9, 14, 23]. Given the chronic nature of FH, it is essential that an intervention enables individuals to make lifelong behavioural changes, rather than for the short duration of a clinical trial. As the greatest reduction in CVD risk is achieved when treatment is started at a young age [4, 24], young people stand to gain the most for receiving a lifestyle intervention. Given the increasing diagnostic rates and high prevalence of FH, any intervention to be evaluated should also be pragmatic and feasible to provide within the constraints of current healthcare services.

In the current research, we propose to develop and evaluate a family-based nutritional and physical activity intervention to enable young people with FH, and their affected parent, to achieve dietary and physical activity recommendations thus reducing their CVD risk. As there is currently insufficient evidence to support a large, full-scale RCT to evaluate such an intervention, a pilot RCT is needed as advised in the British Medical Research Council (MRC) framework for the development and evaluation of complex interventions [25, 26].

### Study aims and objectives

The primary aim of this study is to provide evidence for the feasibility and acceptability of conducting a future adequately powered, randomised controlled trial to evaluate a specifically designed nutrition and physical activity intervention in young people and their affected

For peer review only

parent with FH. The primary objectives of the study are to explore the following questions:

1. Will young people and their parent be willing to participate in the proposed family-based intervention?
2. Will families accept randomisation to the control or intervention group, adhere to research methods in their allocated group and complete the study?
3. Are the research methods that are used to capture data fit for purpose?
4. Is there sufficient protocol fidelity in the intervention group?
5. Upon completion of the study, was the overall experience of the intervention and/or the study research processes positive and potentially reproducible in an adequately powered trial?

The secondary aim of this study is to estimate the potential efficacy of the intervention on the dietary intake, physical activity and sedentary time, body composition, selected CVD risk factors and quality of life of young people and their affected parent with FH.

## Methods

This protocol is reported in accordance with SPIRIT (Standard Protocol Items: Recommendations for Interventional Trials) guidance and a completed checklist is available in Additional File 1.

### Study design and setting

This pilot study is a two-arm, randomised waitlist-controlled trial comparing a specifically developed nutrition and physical activity intervention against usual-care waitlist control amongst young people with FH. This study aims to recruit family units, each comprised of a young person-parent dyad. This is in recognition of the autosomal dominant presentation of FH which means all affected young people will have one affected parent. This will also facilitate the proposed family-based delivery of the intervention. This study will be conducted across three National Health Service (NHS) Foundation Trust sites in England, UK: University Hospitals Bristol, Royal United Hospitals Bath and St. George's University Hospitals London (Fig. 1).

### Study population

In this family-based intervention, young person(s) aged 10–18 years and their affected parent with FH will be invited to participate. A family unit will be comprised of at least one adult and one young person with FH, but there may also be family units in which additional young family members with FH take part.

#### *Eligibility criteria*

Individuals aged 10–18 years with a genetically confirmed clinical diagnosis of heterozygous FH who receive their care from the paediatric or adult lipid clinics at the three NHS Foundation Trust sites will be invited to join the study. Their parent (≥ 18 years), with a genetically confirmed clinical diagnosis of heterozygous FH, will also be invited to join the study.

Pregnant female patients, or those planning pregnancy, and individuals who are unable to give informed consent or assent for those aged 10–15 years are not eligible for participation. Patients with a diagnosis of homozygous FH and patients not established on their current treatment regimen for at least 1 month prior to recruitment to the study are not eligible for participation.

If a young person with FH wishes to join the study but their affected parent is deceased or does not want, or is not eligible to participate, then the young person will still be able to participate. In this instance, a non-affected parent or main carer of the young person will be invited to take part to facilitate the family-based aspect of the study if they wish. However, blood samples will not be collected from these non-affected participants. In instances where the non-affected parent or main carer also does not wish to take part, the young person with FH will still be able to participate on their own.

### Recruitment

#### *Identification*

All young people receiving care from paediatric or adult lipid clinic services at participating sites will be screened for eligibility. The eligibility of their affected parent will then be considered. This will be carried out by a member of the hospital care team and will be repeated every 3 months during the recruitment phrase. This will be conducted at both paediatric and adult clinics because young adults often transition to adult services before the age of 18 years.

#### *Invitation to participate*

A study invitation letter and an age-appropriate participant information sheet (PIS) will be sent to the families of young people who are identified and deemed to meet the inclusion criteria from review of medical records. A PIS will also be included for the parent of the identified young person. Contact details of the research team are provided in this PIS and families interested in participation are asked to make contact. The study will also be described, and a PIS provided, to young people and their parent(s) attending either paediatric or adult lipid clinics. For families expressing an interest in participation, a meeting or phone call will be arranged with a member of the research team.

For peer review only - http://bmjopen.bmj.com/site/about/guidelines.xhtml

Kinnear *et al. Pilot and Feasibility Studies* (2020) 6:42 

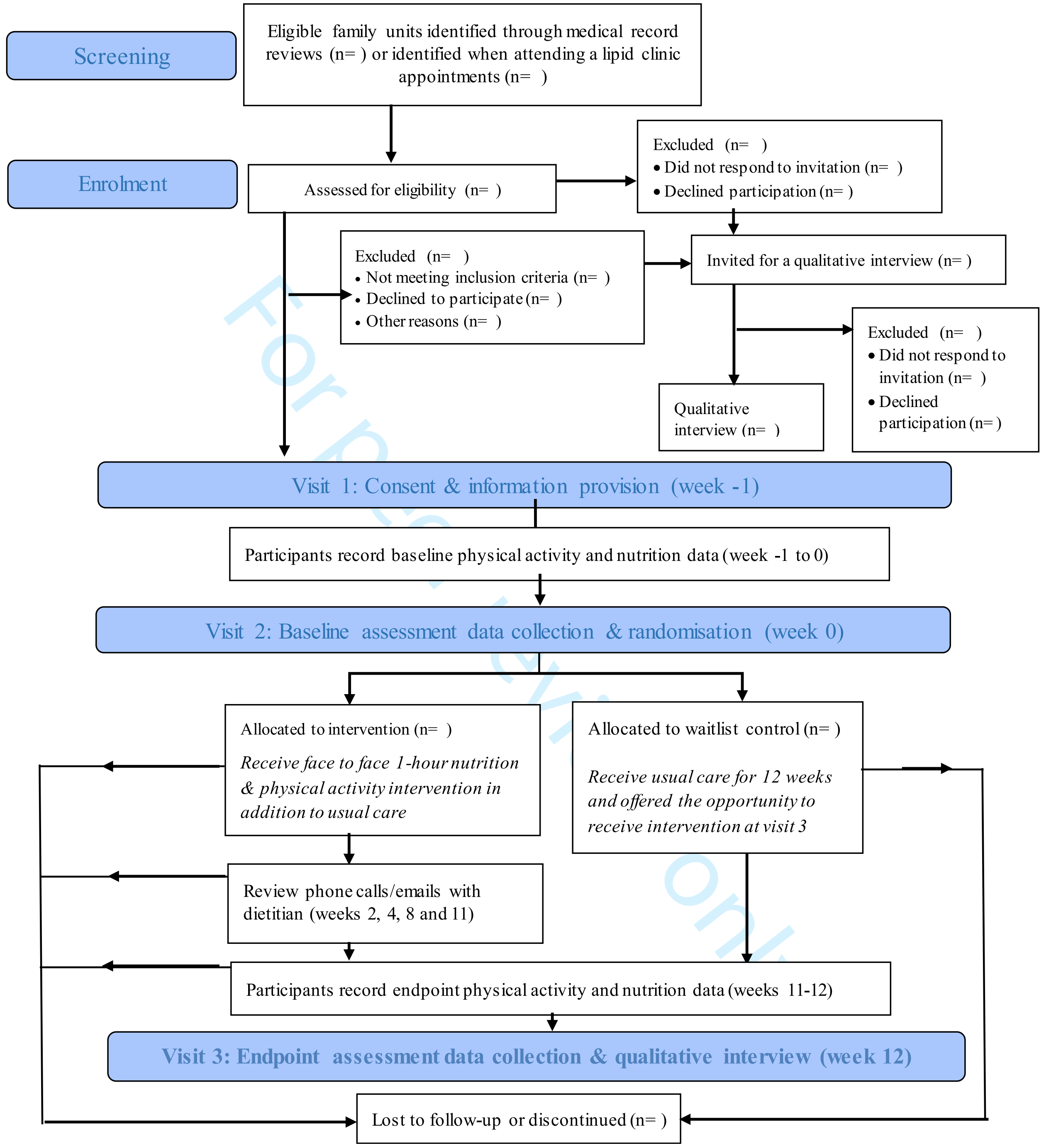


**Fig. 1** Study flow diagram

#### Sample size

This is an exploratory feasibility study and therefore no power calculation has been used to determine sample size because it is not designed or powered to address the effectiveness of the intervention being evaluated [25, 27, 28]. Sample sizes of between 24 and 50 have been recommended for feasibility studies wishing to provide a standard deviation estimation to inform the sample size calculation of a larger RCT [27, 29, 30]. The target sample size of 24 family units in this study has been based upon this recommendation in addition to knowledge of the current local FH population.

#### Randomisation and blinding

Randomisation, stratified by study site, will allocate family units to either usual care and waitlist (control) or to the

For peer review only - http://bmjopen.bmj.com/site/about/guidelines.xhtml

nutrition and physical activity intervention (intervention) on a 1:1 basis. To facilitate recruitment and retention, family units allocated to the control arm will be placed on a waitlist to receive the intervention at the end of the 12-week study period. Randomisation will be carried out by an independent person using prepared, password-protected randomised lists, following obtainment of informed consent to participate. Family units will be notified of their assigned group at research visit two, after baseline data collection. Due to the nature of the intervention, delivery and study design, it is not possible to blind participants, clinical staff or research staff to the randomisation results. Upon completion of the study, participants in the control group will be asked if they had any communication with other young person-parent dyads in the study. This will assess potential intervention contamination bias which will be taken into consideration in the analysis. The anticipated likelihood of this occurring is low due to the infrequency of outpatient appointments (usually once a year) that families attend.

### Intervention

Family units randomised to the intervention group will receive an individualised, family-based nutrition and physical activity intervention developed specifically for this study. It will be delivered to the family at research visit two by a dietitian and will last approximately 1 h. This will be followed up with four phone calls or email correspondence (according to participant preference) at weeks 2, 4, 8, and 11 of the intervention. Age-specific intervention booklets (10–13 years and ≥ 14 years) have been developed for use in the intervention. These will be provided to the participants at research contact two to enable participants to refer to the intervention information during the 12-week intervention and follow-up sessions. If a young person is participating without a parent, the intervention content and delivery will be changed to facilitate this.

#### *Intervention content and delivery*

The development and content of the intervention has been described in full elsewhere [31], but will be described briefly. Intervention aims to enable participants to achieve the dietary intakes and physical activity levels currently recommended for people with FH. A literature review of the available guidelines for the management of FH was conducted to determine the following targets for daily dietary intakes and weekly physical activity levels:

1. Total fat intake ≤ 30% of total energy intake (TEI)
2. Saturated fat intake of ≤v10% TEI achieved via replacement of saturated fats with monounsaturated and polyunsaturated fats
3. Dietary cholesterol intake ≤ 300 mg
4. Consumption of ≥ 5 portions of fruit and vegetables
5. Age appropriate fibre intake: 10-year-olds = 20 g/day; 11–16 year-olds = 25 g/day and 30 g/day for ≥ 17 years
6. 2 g of plant stanol/sterols
7. Reduce time spent engaged in sedentary behaviours
8. Age appropriate physical activity levels:

- Adults: ≥ 150 min a week of moderate intensity physical activity or ≥ 75 min of vigorous intensity physical activity, or a mixture of the two. Additional activity focussing on improving muscle strength should be undertaken twice a week.
- Young people: ≥ 60 min of moderate-to-vigorous physical activity (MVPA) each day, with three of these sessions each week being of vigorous intensity and including activities that strengthen muscle and bone.

Evidence suggests that behaviour change interventions based on theory are more successful than those developed without a theoretical base [32]. As there are many theoretical models, but no consensus on which one is superior, the theoretical domains framework (TDF) was used in the development of this intervention [33]. The TDF brings together 33 psychological theories that are relevant to behaviour change and sorts them into 84 constructs (the component parts of theories) which have been organised into 14 domains (broader areas in which a theory may be applied, e.g. motivation). This TDF can be used as a framework to identify barriers and facilitators to achieving behaviour change and has been validated for use in developing theoretically informed interventions [33]. To develop an understanding of behavioural factors influencing adherence to treatment in people with FH a qualitative evidence synthesis was conducted by our team. This synthesis identified enablers and barriers to treatment adherence in young people and adults with FH [34] which were then mapped onto the TDF domains. Appropriate behaviour change techniques (BCTs) to target these domains were selected using a 93-item BCT taxonomy [35]. The utility of these BCTs in this intervention were considered in relation to the APPEASE criteria: affordability, practicality, effectiveness, cost-effectiveness, acceptability, side-effects/safety and equity [36]. A total of 26 BCTs have been incorporated into the intervention. The intervention will be delivered to all participants by one of two dietitians.

#### *Control group*

Family units assigned to the control group will be informed that they are on a waitlist to receive the intervention at the end of the 12-week study period and

For peer review only

receive usual care. At the three NHS sites involved, usual care is comprised of an annual appointment with a lipid clinic doctor and continuation on their pharmacological treatment, if applicable. Young people are not offered the opportunity to receive dietetic advice when attending paediatric outpatient visits and young people and adults are given an option to receive advice at their first adult clinic outpatient visit. Therefore, it is unlikely that any participant on the waitlist control will receive dietetic advice during the 12-week intervention period; however, this will be monitored. These family units will not be contacted during the 12-week intervention period, except before week 12 to arrange the endpoint dietary and physical activity assessments ahead of research visit three.

### Primary aim outcomes

#### *Feasibility and acceptability outcomes (study objectives 1, 2 and 3)*

The feasibility and acceptability will be estimated through recruitment, randomisation and retention rates, attendance at study visits, completion rates of questionnaires, completeness of dietary intake and physical activity data and rates of successful collection of clinical measurements (blood samples, anthropometric measurements and blood pressure).

#### *Intervention fidelity and evaluation outcomes (study objective 4)*

To explore if the intervention is delivered as intended, the dietitians will complete checklists and record detailed reflections for each intervention and follow-up session. Details of the goals set, and self-reported attainment by participants during follow-up sessions, will be recorded in case report forms (CRFs). Intervention intensity will be estimated through recording the number and duration of any face to face, email and telephone contact with participants. The potential mechanisms of impact will be explored through descriptive analysis of this data, along with qualitative data collection from participants in the intervention group. Exploration of these outcomes will enable the intervention to be refined before utilisation in future studies.

#### *Qualitative outcomes (study objective 5)*

The acceptability of the intervention and the research methods used will be explored through additional data regarding the acceptability of the intervention and research methodology collected from semi-structured qualitative interviews. The sub-sample selected for interview will comprise of up to 30 participants based on sample sizes in previous similar studies [37–42]. It will also include participants who did not want to take part in the full research study but consented to the qualitative component of the study (Fig. 1). A purposive sampling approach, using a maximum variation method, will be taken to select participants for the sub-sample according to individual characteristics, opinions and experiences relevant to this study [43, 44]. Separate topic guides have been developed for adults and young people to provide some structure to the interviews.

The qualitative data will also be used to help understand how and why the intervention was effective or ineffective and if the intervention will be successful in a wider context [26, 45, 46]. It will also be used to help improve the design and conduct of any future trials [26, 47].

### Secondary aim outcomes

The potential efficacy of the developed intervention upon dietary intakes, physical activity and sedentary time, body composition, CVD risk factors and quality of life will be estimated. This will be done through measurement of changes in a range of clinical and behavioural outcomes before and after the intervention period. Results will also be used to estimate the variability of each outcome measure and to identify the suitability of the outcome tools/measures chosen to detect any changes. When possible, standard deviation of the outcome measure will be calculated to inform the sample size calculation of a future large-scale RCT.

Behavioural outcomes (dietary intake, physical activity, sedentary time) will be assessed in the week immediately preceding research visits two and three (Fig. 1). Anthropometric, clinical and biochemical outcomes, and quality of life measures will be collected at baseline and endpoint assessments conducted at research visits two and three (Fig. 1). Further details of each outcome are provided below and in Table 1.

#### *Dietary intake*

Participants will record their dietary intake over four non-consecutive days (including one weekend day) using INTAKE24, an online 24-h recall tool. INTAKE24 is a validated, self-completed computerised dietary recall system based on multiple-pass 24-h recall developed specifically for young people. It is a secure tool, accessed by individually issued log-on details. It has been found to produce mean daily energy and nutrient intakes comparable with interviewer-led 24-h recalls in young people aged 11–24 years, at a significantly lower cost [48]. This method offers several benefits over traditional paper-based methods, such as pre-programmed completeness checks, prompts and food photographs to improve data capture. The automated coding system ensures consistency of coding and removes the risk of data entry errors. Furthermore, newer technology-based methods of dietary assessment have been demonstrated to be preferred by young people and adults compared to traditional methods [49–52]. Participants will

For peer review only

**Table 1** Outcome measures, data collection methods and time points

| Study aim | Objectives being explored | Outcome measures | Data collection method/tool | Time point collected (weeks) | | | | |
|---|---|---|---|---|---|---|---|---|
| | | | | − 1 | − 1 to 0 | 0 | 11–12 | 12 |
| Primary: Feasibility and acceptability | 1. Will young people and their parent be willing to participate in the proposed family-based intervention?<br>2. Will families accept randomisation to the control or intervention group, adhere to research methods in their allocated group and complete the study?<br>3. Are the research methods that are used to capture data fit for purpose? | Recruitment, retention, randomisation and adherence to protocol rates | Screening, recruitment and randomisation logs, monitoring of study visit attendance, successful collection of clinical outcome measures and withdraw/loss to follow up records. | Continuous during data collection period ⟷ | | | | |
| | 4. Is there sufficient protocol fidelity in the intervention group? | Intervention intensity and adherence to intervention protocol and delivery | Self-completed checklists and recording of the number & duration of contact with participants | | | | | |
| | 5. Upon completion of the study, was the overall experience of the intervention and/or the study research processes positive and potentially reproducible in an adequately powered trial? | Self-reported experiences and beliefs of participants regarding the study methodology &/or intervention | Semi-structured qualitative interviews | | | | | x |
| Secondary: Potential efficacy | No specific objectives stated for this secondary study aim | Patient characteristics: medication, education levels, smoking status and FH genetic variant | CRF developed for study | x | | x | | x |
| | | QoL score (HRQoL and component physical, emotional, social and school functioning scores for young people and single index value and VAS rating for adults) | Age appropriate PedsQL™ QoL inventory or EUROQOL 5D-3L health questionnaire | | | x | | x |
| | | Mean fat, saturated fat, monounsaturated fat, polyunsaturated fat, fibre, plant sterol/stanols, cholesterol and fruit and vegetable intake per day (grams and % of daily energy intake) | INTAKE24 online dietary recall system completed for 4 days | | x | | x | |
| | | MVPA (min/day) | ActiGraph GT3X+ accelerometer worn for 7 days | | x | | x | |
| | | Time spent sitting/lying, standing and walking (min/day) | activPAL3 accelerometer worn for 7 days | | x | | x | |
| | | Self-reported physical activity levels (MET minutes per week and categorised into high, medium or low level groups for adults and assigned score between 1 and 5 for young people, in which 1 indicates low levels and 5 high levels, of PA) | Age appropriate IPAQ, PAQ-A or PAQ-C questionnaire | | x | | x | |

For peer review only

**Table 1** Outcome measures, data collection methods and time points *(Continued)*

| Study aim | Objectives being explored | Outcome measures | Data collection method/tool | Time point collected (weeks) | | | | |
|---|---|---|---|---|---|---|---|---|
| | | | | − 1 | − 1 to 0 | 0 | 11–12 | 12 |
| | | Anthropometric measurements: height (cm), weight (kg), BMI (kg/m$^2$), body composition including body fat (%) and fat free mass (%) | Tanita body composition analyser and stadiometer | | | x | | x |
| | | Systolic and diastolic blood pressure (mmHg) | Mean of two measures by a sphygmomanometer (three measures if 1st and 2nd differ by more than 10mmHg) | | | x | | x |
| | | Serum concentrations (mmol/l) and component lipid breakdown (mmol/l and %) of different classes (very large, large, medium and small) of VLDL, LDL, HDL particles; mean diameter of VLDL, LDL & HDL particles (nm); serum concentration of total cholesterol, LDL, HDL, HDL2, HDL3, remnant cholesterol, triglycerides (mmol/l) and Apolipoproteins (g/l). | Metabolomic analysis of serum samples obtained from processing of 25ml whole blood samples | | | x | | x |

*CRF* case report form, *QoL* quality of life, *VAS* visual analogue scale, *MVPA* moderate-to-vigorous physical activity, *IPAQ* International Physical Activity Questionnaire, *PAQ-A* the Physical Activity Questionnaire for Adolescents, *PAQ-C* the Physical Activity Questionnaire for older Children, *BMI* body mass index, *VLDL* very large density lipoprotein, *LDL* low-density lipoprotein, *HDL* high-density lipoprotein

be asked about their consumption of supplements and plant stanol/sterol fortified foods at research visits 2 and 3.

The data will be analysed to produce mean daily intakes of fat, saturated fat, monosaturated fat, polyunsaturated fat, cholesterol, plant stanols/sterols, fibre and fruits and vegetables.

#### *Physical activity levels*

Free-living MVPA will be measured using an ActiGraph GT3X+ accelerometer (ActiGraph, Pensacola, USA), worn by participants on an elasticated belt on the waist above the midline of the right thigh for seven consecutive days, except for water-based activities. This is a tri-axial device that detects the frequency and amplitude of acceleration in three axes. Participants will also be asked to complete a physical activity log book, to capture waking/sleeping times and any activities which required the monitor to be taken off. When processing the data, age- and sex-specific cut points will be applied to define sedentary time, light, moderate and vigorous physical activity during waking hours. Total daily time spent in MVPA will be obtained by totalling the duration of all moderate and vigorous physical activity bouts for each day. This value will then be averaged over the number of valid days to determine mean time spent in MVPA per day.

Self-report measures of physical activity have been found to overestimate MVPA in young people [53] and adults [54]. However, self-report and objective measures have not been compared within populations of individuals with FH. Self-report measures are more practical and less burdensome for study participants and may be a more appropriate outcome measure to use in a future larger scale trial. Thus, each participant will also complete age appropriate physical activity self-report questionnaires. For adults, the International Physical Activity Questionnaire (IPAQ) will be used which is a validated self-report tool for measuring physical activity in adults aged 18–64 years old [55]. The Physical Activity Questionnaire for Older Children (PAQ-C) [56] and Adolescents (PAQ-A) [57] will be used with participants aged 10–14 years and 15–18 years old respectively, as recommended by a comprehensive systematic review and expert panel consensus [58]. These questionnaires will be scored to assign individuals into one of five categories which indicate low to high levels of physical activity. These will be compared to the objective MVPA measurements.

#### *Sedentary time*

This will be measured using an activPAL3 accelerometer (PAL Technologies Ltd., Glasgow, UK) worn on the front of the thigh. The activPAL3 will be made waterproof using a nitrile sleeve and a waterproof hypoallergenic medical dressing will be used to attach it to the leg, enabling participants to wear it continuously for 24 h/day over 7 days. It contains a tri-axial accelerometer

For peer review only

which responds to signals related to gravitational forces and provides information on thigh inclination [59]. This allows for precise differentiation between postures in prolonged free-living activities [60] and represents a valid measure of time spent sitting/lying, standing and walking in adults [61, 62] and young people [63–65]. Total daily time spent sitting/lying, standing and walking will be obtained for each day and will be averaged over the number of valid days across all waking hours.

#### Participant characteristics

Information about smoking status, education, medication and FH genetic variation will be obtained from participants and used to characterise the intervention and control groups.

#### Anthropometric and other clinical measurements

Height and weight will be measured to determine body mass index (BMI). Resting systolic and diastolic blood pressure will be estimated by averaging two separate measurements using a sphygmomanometer. Blood pressure and BMI have been chosen as outcome measures due to research finding these to be independently associated with CVD risk in adults with FH [66]. There has been no similar research carried out in young people with FH; however, BMI and blood pressure have been reported to independently influence future CVD risk in young adults without FH [67].

Body composition will be measured using bioelectrical impedance (Tanita™ MC-780MA) with attention paid specifically to fat and fat-free mass. While no research to date has investigated the role of these outcomes measures upon CVD risk in individuals with FH, they have been found to influence the serum blood lipid levels of young people without FH [68]. Therefore, these measures will help to characterise the participants in the control and intervention groups and allow for interpretation of any changes in serum lipid levels observed in participants before and after the intervention period. Furthermore, changes in these outcomes have been found to moderate the influence of physical activity interventions upon blood lipid measures in adults without FH [69, 70] highlighting the need for future research to control for such measures.

#### Quality of life

The need for further research to explore the potential detrimental impacts that individuals with FH may experience as a result of making changes to their diet and physical activity levels was highlighted in a Cochrane systematic review [20]. Therefore, the Quality of life (QoL) of all participants will be measured.

Young person participants will be asked to complete an age-appropriate Pediatric Quality of Life Inventory ™ (PedsQL™) Version 4.0. The PedsQL 4.0 Generic Core Scales will be used which have been validated for measuring the health-related quality of life (HRQoL) of young people aged 2 to 18 years with chronic health conditions [71]. This multidimensional self-report scale consists of 23 items, covering four domains of HRQoL: physical, emotional, social and school. The completed inventories will be scored using the recommended scoring system [72] to produce a score for HRQoL and a breakdown of the component physical, emotional, social and school functioning scores.

Adult participants will be asked to complete an EuroQol Group EQ-5D-3L health questionnaire as recommended by NICE [73] and validated for use in European adults [74]. Participants will be asked to rate their degree of impairment in five health domains (mobility, self-care, usual activities, pain/discomfort and anxiety/depression) using one of three responses (no problems, some problems and extreme problems). The recommended scoring system will be used [75] which produces a five-digit health state profile representative of five dimensions of health. This will then be converted into a single index value using a validated value set created for use in the UK [76]. Participants will also be asked to rate their perceived health by drawing a line to a point on a visual analogue scale (VAS) between the endpoints of 'best imaginable health state' and 'worst imaginable health state'. The VAS data will be presented as a single value between 1 and 100.

#### Blood lipidomic markers

The primary aim in the treatment of FH is a reduction in LDL-C concentrations [15]. However, it is recognised that focussing on a single lipid marker does not adequately reflect an individual's overall risk of CVD or the clinical effect of any pharmacological or lifestyle treatment [17, 77]. Therefore, several lipidomic markers have been selected as outcome measures (Table 1), although these may change as the literature develops. These have been chosen on the basis of their proposed role in the prediction and development of CVD risk [17, 78] and/or sensitivity to change via dietary intakes and physical activity as proposed in previous research [77, 79].

A maximum of 25 ml whole blood will be collected from each participant which is within the safe limits advised by the World Health Organisation (WHO) for use in child health research [80]. Blood samples will be collected, stored and analysed in accordance with the Human Tissue Act 2004. Participants will be requested to fast for a minimum of 6 h and to avoid strenuous physical activity for 12 h prior to sample collection.

Samples will be sent to the laboratory of the hospital in which they were collected and will be processed

For peer review only - http://bmjopen.bmj.com/site/about/guidelines.xhtml

according to the laboratory Standard Operating Procedures and under the supervision of the laboratory team. Once centrifuged, the sample will be split whereby some of the sample will be analysed immediately to determine the full lipid profile (LDL-C, HDL and total cholesterol) in the hospital laboratory. The remainder of the sample will be stored at − 80 °C as plasma in anonymously labelled microtubes in the hospital laboratory prior to transport, contained in dry ice, to the University of Bristol MRC Integrative Epidemiology Unit (IEU) Metabolomics Facility. These samples will undergo batch metabolomic analysis at the end of the data collection period to determine the levels of lipidomic markers.

## Data collection

### *Quantitative data*

All quantitative participant data will be collected on anonymised purpose-designed CRFs or questionnaires. These data will then be inputted into a study database (Microsoft Access) by a member of the research team. The database is password-protected and accessible only by the chief and co-investigators.

Data from the activPAL and ActiGraph GT3X+ accelerometers will be downloaded using custom software (PAL Technologies Ltd., Glasgow, UK and ActiGraph, Pensacola, USA, respectively). Raw data from the ActiGraph GT3X+ accelerometer will be downloaded and analysed using the manufacturer's software (ActiLife software v6.13.4; ActiGraph, Pansacola, FL, USA). The activPAL3 data will be downloaded using the manufacturer's software (PAL software v7.2.38; PAL Technologies Ltd., Glasgow, UK), and the raw data will be cleaned and processed using a validated algorithm [81]. The participant's physical activity log-books will be used to aid identification of non-wear periods and sleep time; these periods will be removed from subsequent analysis. Data collected using the INTAKE24 online tool will be downloaded and analysed manually using Excel and statistical software.

### *Qualitative data*

The audio-recorded qualitative data collected will be transcribed verbatim by a member of the research team, or an individual from a University of Bristol approved transcription company, and stored using NVivo (NVivo10, QSR International, 2012) data management software.

## Withdrawal

Each participant will have the right to withdraw from the study at any time. The study design aims to minimise attrition by not overburdening participants but if there is significant attrition (loss of more than 15 individuals) over the study period, additional individuals with similar characteristics to those lost will be recruited. Participants will be withdrawn from the study if their hospital FH care team feel that their participation is negatively affecting their clinical status. This includes participants who become pregnant during the study as they will be required by necessity to stop statin treatment. A member of the hospital FH care team will inform the research dietitian of these cases. In all cases, data collected up until the point of withdrawal will be included in the analyses, unless the participant expresses a wish for their data to be destroyed. If a reason for withdrawal is given, this will be documented in the participants CRF.

## Analysis plan

Data collected from young people will be analysed separately from the data collected for adult parent participants.

### *Quantitative data*

Recruitment, randomisation and retention rates, attendance at study visits, completion rates of questionnaires, completeness of dietary intake and physical activity data, rates of successful collection of clinical measurements and categorisation results of self-reported physical activity levels will be descriptively analysed. The other quantitative variables of interest (Table 1) are continuous. Therefore, where possible, groups will be summarised by means and standard deviations (S.D.) for (i) baseline, (ii) end point assessment and (iii) for the changes over the intervention period. This will be carried out for individuals in the control and intervention groups.

Comparisons will be made between the endpoint assessment means in the intervention and control groups with or without adjustment for the baseline values and stratification, as appropriate. 95% confidence intervals (C.I.) will be reported for mean differences. Comparisons may be made between the intervention and control groups in respect of the mean changes from baseline if this is found to improve precision [82].

Additionally, the dietary intake and physical activity data at both baseline and endpoint assessment will be compared to the target levels determined for the intervention (outlined previously in 'intervention content') and will be descriptively analysed for participants in the control and intervention groups.

### *Qualitative data*

Interview transcripts will be analysed using thematic analysis [83, 84]. NVivo (NVivo10, QSR International, 2012) data management software will be used to manage the transcript data. Thematic analysis involves coding the interview transcripts, using an inductive approach. This step will be carried out by two members of the research team who will independently code data on a subsample of the data set. The analysts will then discuss their initial coding and endeavour to reach consensus

For peer review only - http://bmjopen.bmj.com/site/about/guidelines.xhtml

about the codes assigned with the aim of developing a definitive coding frame for the complete data set. Once all transcripts have been coded inductively, the research team members will work deductively to group codes into themes associated with the research objectives of this study. A descriptive, narrative approach will then be taken to explore the findings in the context of the trial research aims and their experiences of participation in the trial. The findings will be presented using anonymised participant quotes to illustrate any identified themes.

### Harms

There are no known risks to the participants in this study. Any adverse event, regardless of relationship with the study treatment, will be recorded and reported in accordance with University Hospitals Bristol NHS Foundation Trust and the University of Bristol (as sponsor) guidance.

### Data monitoring

A Data and Safety Monitoring Board has not been formally established, due to the low risk nature of the intervention. All adverse events will be reported to the chief investigator (FJK) and reviewed at monthly meetings with co-investigators (JPHS and FEL) to determine whether the study should be discontinued because of participant safety.

### Auditing

The University of Bristol, as Sponsor of this study, has a Service Level Agreement in place with University Hospitals Bristol NHS Foundation Trust. As part of this agreement, University Hospitals Bristol NHS Foundation Trust will undertake monitoring of research projects where University of Bristol is fulfilling the responsibilities of Research Sponsor. A minimum of 10% of projects will be monitored. If this study is selected for audit, it would be carried out independently to the study team and Sponsor.

## Ethics and Dissemination

### *Research ethics approval*

The study has been approved by the South West-Cornwall & Plymouth Research Ethics Committee (REC) (Reference: 18/SW/0121).

### *Protocol amendments*

Any amendments to the protocol will be submitted to the REC and Health Research Authority (HRA) for approval, after permission is sought from the Sponsor. Only once the amendment has been approved by the REC and HRA and has received confirmation of continued capacity and capability from NHS sites (or acknowledged in the case of a minor amendment) will the amended protocol be implemented. Deviations to the protocol outlined in the published protocol will be highlighted in any subsequent publication of the study findings.

### *Consent and assent*

Written informed consent is required from all participants to participate in this study. For young persons aged 10–15 years, this will be obtained from their parent or guardian, with written informed assent also collected from the young person. Informed consent will be sought when patients attend the hospital for a routine clinic appointment or at the first research visit, prior to commencing any data collection. A member of the research team will be responsible for the consent process. There will be an opportunity to discuss any remaining questions the participant may have with a member of the research team prior to signing the consent or assent form. A copy of the signed assent and/or consent form will be filed in the participants' medical records to ensure that the hospital FH care teams are aware of their involvement in the study. Eligible individuals who decline participation in the study will be offered the option to undertake the qualitative component of the research study only. These individuals will receive a different PIS outlining what is involved in this part of the study. These individuals will be required to give informed consent or assent prior to collection of any data.

All individuals will be advised that participation in the study is voluntary and that they have the right to withdraw at any time, without the need for explanation and that their decision will not impact upon the care they receive.

### *Confidentiality*

All data will be anonymised, with participants identified by a unique study number. Confidentiality will be ensured through the application of the University of Bristol information security and data handling policies. These are compliant with the Data Protection Act 2018 and conform to the security standards of the NHS IG toolkit. All participant information (i.e. names, addresses, dates of birth etc.) will be stored separately from data collected during the study (i.e. dietary intake) and participants will be identified by a unique study number. Personal identifiable paper records will be stored separately from anonymised paper records and CRFs. All paper records will be stored in secure storage facilities in University Hospitals Bristol, Royal United Bath Hospital or St. George's Hospitals NHS trust sites. No personal data will leave the sites until archiving at the end of the study. Electronic data will be stored securely using password-protected databases on a University of Bristol computer in the NIHR Biomedical Research Centre (Nutrition Theme), Level 3 University Hospitals Bristol NHS Trust Education and Research Centre. This is a secure site within the Trust. The University of Bristol computer hard drive is also encrypted. Only members of the study team will have access to the dataset.

For peer review only

*Dissemination policy*

Summaries of preliminary findings will be produced for study participants and the individual results for each participant will be recorded in their medical records.

Manuscripts reporting the findings will be produced for publication in relevant, peer-reviewed international journals. As this is a feasibility study, the results will be used to inform and develop a larger RCT, if appropriate, which will be adequately powered to detect clinically significant changes in outcome measures.

## Patient and public involvement

Two events have taken place with patient and public involvement:

1. Seven individuals, three young people and four adults, with FH under the care of the University Hospitals Bristol NHS foundation Trust have participated in the development work underpinning this research. They gave feedback about the initial design of the intervention and data collection methods.
2. Ten young people (aged 11–17 years) from the Bristol Young Persons Advisory Group (YPAG) also participated in the development work underpinning this research. This group is comprised of young people who are interested in healthcare and research who meet regularly in Bristol to help researchers with their projects. This group reviewed and gave feedback on the participant facing documents, data collection methods and details of the intervention involved with this study.

## Discussion

Dietary intakes and physical activity are considered key components of managing the CVD risk for individuals with FH. However, there is a recognised lack of research to support the efficacy of adhering to the current guidelines for promoting a favourable CVD risk factor profile. Furthermore, the best approach to promote behavioural changes in this population is yet to be determined. This paper describes the protocol of a pilot, randomised waitlist-controlled trial designed to estimate the feasibility and acceptability of conducting a family-based nutrition and physical activity intervention to young people, and their affected parent, with FH. The data obtained in this trial could be used to inform the development of a future clinical trial powered to detect clinically significant changes in the chosen outcome measures, if deemed appropriate.

### Study status

The study is now closed for recruitment and data collection was completed in March 2020.

## Supplementary information

**Supplementary information** accompanies this paper at https://doi.org/10.1186/s40814-020-00584-3.

**Additional file 1.** SPIRIT 2013 statement checklist: recommended items to address in a clinical trial protocol and related documents.

### Abbreviations

FH: Familial hypercholesterolaemia; LDL-C: Low-density lipoprotein cholesterol; CVD: Cardiovascular disease; HCP: Healthcare professional; RCT: Randomised controlled trial; MRC: Medical Research Council; SPIRIT: Standard protocol items: recommendations for interventional trials; NHS: National Health Service; PIS: Participant information sheet; TEI: Total energy intake; MVPA: Moderate-to-vigorous physical activity; TDF: Theoretical domains framework; BCT: Behaviour change technique; CRF: Case report form; IPAQ: International physical activity questionnaire; PAQ-C: The physical activity questionnaire for older children; PAQ-A: The physical activity questionnaire for adolscents; BMI: Body mass index; QoL: Quality of life; HRQoL: Health-related quality of life; NICE: National Institute for Health and Care Excellence; VAS: Visual analogue scale; WHO: World Health Organisation; IEU: Integrative Epidemiology Unit; CI: Confidence intervals; REC: Research Ethics Committee; HRA: Health Research Authority; YPAG: Young Persons Advisory Group

### Acknowledgements

DJS and AET acknowledge support from the National Institute for Health Research NIHR Leicester Biomedical Research Centre. The views expressed are those of the authors and not necessarily those of the NHS, the NIHR or the Department of Health and Social Care.

### Trademarks

PedsQL ™, copyright © 1998 JW Varni, Ph.D. All rights reserved. Contact information and permission to use: Mapi Research Trust, Lyon, France. Internet: https://eprovide.mapi-trust.org and www.pedsql.org.

### Authors' contributions

FJK designed the study and wrote the manuscript for publication. FEL, JPHS, DJS, AS and AET contributed to the design of the study and provided feedback at all stages of its development and the writing of this protocol. JPHS and GB provided their expertise as clinicians working with individuals with FH with respect to study design and recruitment processes and reviewed this protocol. All authors reviewed the manuscript and approved the final version.

### Funding

This study is funded by the National Institute for Health Research (NIHR) Bristol Biomedical Research Centre (Nutrition Theme) at University Hospitals Bristol NHS Foundation Trust and The University of Bristol. The views expressed in this publication are those of the authors and not necessarily those of the NHS, the NIHR or the Department of Health and Social Care.

### Availability of data and materials

Not applicable.

### Ethics approval and consent to participate

Ethical approval for this project was granted by the South West-Cornwall & Plymouth Research Ethics Committee (Reference: 18/SW/0121).

### Consent for publication

Not applicable.

### Competing interests

None declared.

### Author details

[1]NIHR Bristol Biomedical Research Centre (Nutrition Theme), University Hospitals Bristol NHS Foundation Trust and the University of Bristol, Bristol, UK. [2]National Centre for Sport and Exercise Medicine, School of Sport, Exercise and Health Sciences, Loughborough University, Epinal Way, Loughborough, UK. [3]University Hospitals of Leicester NHS Trust, Infirmary

For peer review only - http://bmjopen.bmj.com/site/about/guidelines.xhtml

Square, Leicester, UK. [4]Department of Clinical Biochemistry, University Hospitals Bristol NHS Foundation Trust, Bristol, UK.

Received: 29 April 2019 Accepted: 11 March 2020
Published online: 02 April 2020

## References

1. Goldstein, J.H., HH; Brown, MS, Familial hypercholesterolemia, in The Metabolic and Molecular Bases of Inherited Disease, A. Sciver CR, Sly WS, Valle D. editors, Editor. 2001, McGraw-Hill: New York. p. 2863-2913.
2. Wong B, et al. Cardiovascular disease risk associated with familial hypercholesterolemia: a systematic review of the literature. Clin Ther. 2016; **38**(7):1696–709.
3. Akioyamen, L.E., et al., Estimating the prevalence of heterozygous familial hypercholesterolaemia: a systematic review and meta-analysis. BMJ Open, 2017. **7**(9).
4. Neil A, et al. Reductions in all-cause, cancer, and coronary mortality in statin-treated patients with heterozygous familial hypercholesterolaemia: a prospective registry study. Eur Heart J. 2008;**29**(21):2625–33.
5. Hopkins, P.N., Putting into perspective the hazards of untreated familial hypercholesterolemia. Journal of the American Heart Association, 2017. **6**(6).
6. Kruse G, et al. Cardiovascular disease and its risk factors in patients with familial hypercholesterolemia: a systematic review. J Clin Exp Cardiolog. 2017;**8**(539):2.
7. Villa G, et al. Prediction of cardiovascular risk in patients with familial hypercholesterolaemia. European Heart Journal - Quality of Care and Clinical Outcomes. 2017;**3**(4):274–80.
8. Bogsrud MP, et al. LDL-cholesterol goal achievement, cardiovascular disease, and attributed risk of Lp(a) in a large cohort of predominantly genetically verified familial hypercholesterolemia. J Clin Lipidol. 2019.
9. Humphries SE, et al. The UK Paediatric familial hypercholesterolaemia register: statin-related safety and 1-year growth data. Journal of Clinical Lipidology. 2017.
10. de Sauvage Nolting PR, et al. Prevalence and significance of cardiovascular risk factors in a large cohort of patients with familial hypercholesterolaemia. J Intern Med. 2003;**253**(2):161–8.
11. Jansen AC, et al. The contribution of classical risk factors to cardiovascular disease in familial hypercholesterolaemia: data in 2400 patients. J Intern Med. 2004;**256**(6):482–90.
12. Besseling J, et al. Severe heterozygous familial hypercholesterolemia and risk for cardiovascular disease: A study of a cohort of 14,000 mutation carriers. Atherosclerosis. 2014;**233**(1):219–23.
13. Gaudet D, et al. Relationships of abdominal obesity and hyperinsulinemia to angiographically assessed coronary artery disease in men with known mutations in the LDL Receptor Gene. Circulation. 1998;**97**(9):871.
14. Broekhuizen K, et al. Can multiple lifestyle behaviours be improved in people with familial hypercholesterolemia? Results of a parallel randomised controlled trial. PLoS One. 2012;**7**(12):e50032.
15. Nordestgaard BG, et al. Familial hypercholesterolaemia is underdiagnosed and undertreated in the general population: guidance for clinicians to prevent coronary heart disease : Consensus Statement of the European Atherosclerosis Society. European Heart Journal. 2013;**34**(45):3478–90.
16. NICE, NICE clinical guideline 71: Familial hypercholesterolemia: identification and management. . 2008.
17. Catapano AL, et al. 2016 ESC/EAS Guidelines for the management of dyslipidaemias. European Heart Journal. 2016;**37**(39):2999–3058.
18. Watts GF, et al. Integrated guidance on the care of familial hypercholesterolaemia from the International FH Foundation. Int J Cardiol. 2014;**171**(3):309–25.
19. Lozano, P., et al., U.S. Preventive Services Task Force Evidence Syntheses, formerly Systematic Evidence Reviews, in Lipid Screening in Childhood and Adolescence for Detection of Familial Hypercholesterolemia: A Systematic Evidence Review for the U.S. Preventive Services Task Force. 2016, Agency for Healthcare Research and Quality (US): Rockville (MD).
20. Malhotra A, et al. Dietary interventions (plant sterols, stanols, omega-3 fatty acids, soy protein and dietary fibers) for familial hypercholesterolaemia. Cochrane Database Syst Rev. 2014;6:CD001918.
21. Goldberg AC, et al. Familial hypercholesterolemia: screening, diagnosis and management of pediatric and adult patients: clinical guidance from the National Lipid Association Expert Panel on Familial Hypercholesterolemia. J Clin Lipidol. 2011;**5**(3 Suppl):S1–8.
22. Claassen L, et al. Perceived risk and representations of cardiovascular disease and preventive behaviour in people diagnosed with familial hypercholesterolemia: a cross-sectional questionnaire study. Journal of Health Psychology. 2010;**15**(1):33–43.
23. Thavendiranathan P, et al. Association between physical activity, adiposity, and lipid abnormalities in children with familial hyperlipidemia. European Journal of Cardiovascular Prevention & Rehabilitation. 2007;**14**(1):59–64.
24. Mohrschladt MF, et al. Cardiovascular disease and mortality in statin-treated patients with familial hypercholesterolemia. Atherosclerosis. 2004;**172**(2):329–35.
25. Thabane L, et al. A tutorial on pilot studies: the what, why and how. BMC Med Res Methodol. 2010;**10**:1.
26. Craig P, et al. Developing and evaluating complex interventions: the new Medical Research Council guidance. BMJ. 2008;**337**.
27. Lancaster GA, Dodd S, Williamson PR. Design and analysis of pilot studies: recommendations for good practice. J Eval Clin Pract. 2004;**10**(2):307–12.
28. Leon AC, Davis LL, Kraemer HC. The role and Interpretation of pilot studies in clinical research. Journal of psychiatric research. 2011;**45**(5):626–9.
29. Sim J, Lewis M. The size of a pilot study for a clinical trial should be calculated in relation to considerations of precision and efficiency. J Clin Epidemiol. 2012;**65**(3):301–8.
30. Julious SA. Sample size of 12 per group rule of thumb for a pilot study. Pharmaceutical Statistics. 2005;**4**(4):287–91.
31. Kinnear FJ, Wainwright E, Bourne JE, et al. The development of a theory informed behaviour change intervention to improve adherence to dietary and physical activity treatment guidelines in individuals with familial hypercholesterolaemia (FH). BMC Health Serv Res. 2020;**20**:7. https://doi.org/10.1186/s12913-019-4869-4.
32. Glanz K, Bishop DB. The role of behavioral science theory in development and implementation of public health interventions. Annu Rev Public Health. 2010;**31**:399–418.
33. Cane J, O'Connor D, Michie S. Validation of the theoretical domains framework for use in behaviour change and implementation research. Implementation Science. 2012;**7**(1):37.
34. Kinnear FJ, et al. How do the experiences and beliefs of adults and children with heterozygous familial hypercholesterolaemia influence their adherence to treatment? A systematic review of qualitative evidence protocol. Syst Rev. 2018;**7**(1):120.
35. Cane J, et al. From lists of behaviour change techniques (BCTs) to structured hierarchies: Comparison of two methods of developing a hierarchy of BCTs. British Journal of Health Psychology. 2015;**20**(1):130–50.
36. Mitchie S, A.L., West R, The behaviour change wheel: a guide to designing interventions. 2014, London: Silverback Publishing.
37. Featherston, K. and J.L. Donovan, Random allocation or allocation at random? Patients' perspectives of participation in a randomised controlled trial. BMJ, 1998. **317**(7167): p. 1177.
38. Snowdon C, Garcia J, Elbourne D. Making sense of randomization; responses of parents of critically ill babies to random allocation of treatment in a clinical trial. Social Science & Medicine. 1997;**45**(9):1337–55.
39. Smyth RMD, Jacoby A, Elbourne D. Deciding to join a perinatal randomised controlled trial: Experiences and views of pregnant women enroled in the Magpie Trial. Midwifery. 2012;**28**(4):e538–45.
40. Lawton J, et al. Participating in the United Kingdom Prospective Diabetes Study (UKPDS): a qualitative study of patients' experiences. Br J Gen Pract. 2003;**53**(490):394–8.
41. Luchtenberg M, et al. Young People's Experiences of Participation in Clinical Trials: Reasons for Taking Part. Am J Bioeth. 2015;**15**(11):3–13.
42. Muir LA, George PM, Whitehead L. Using the experiences of people with familial hypercholesterolaemia to help reduce the risk of cardiovascular disease: a qualitative systematic review. Journal of Advanced Nursing. 2012;**68**(9):1920–32.
43. Palinkas LA, et al. Purposeful sampling for qualitative data collection and analysis in mixed method implementation research. Administration and policy in mental health. 2015;**42**(5):533–44.
44. Robinson OC. Sampling in interview-based qualitative research: a theoretical and practical guide. Qualitative Research in Psychology. 2014;**11**(1):25–41.
45. Mann, C., et al., Protocol for a process evaluation of a cluster randomised controlled trial to improve management of multimorbidity in general practice: the 3D study. BMJ Open, 2016. **6**(5).
46. Oakley A, et al. Process evaluation in randomised controlled trials of complex interventions. BMJ. 2006;**332**(7538):413–6.
47. Donovan J, et al. Improving design and conduct of randomised trials by embedding them in qualitative research: ProtecT (prostate testing for cancer and treatment) study. BMJ. 2002;**325**(7367):766.

For peer review only

48. Bradley J, et al. Comparison of INTAKE24 (an Online 24-h Dietary Recall Tool) with Interviewer-Led 24-h Recall in 11–24 Year-Old. Nutrients. 2016; **8**(6):358.
49. Hutchesson MJ, et al. Self-monitoring of dietary intake by young women: online food records completed on computer or smartphone are as accurate as paper-based food records but more acceptable. J Acad Nutr Diet. 2015; **115**(1):87–94.
50. Sharp DB, Allman-Farinelli M. Feasibility and validity of mobile phones to assess dietary intake. Nutrition. 2014;**30**(11-12):1257–66.
51. Boushey CJ, et al. Use of technology in children's dietary assessment. Eur J Clin Nutr. 2009;**63**(**Suppl** 1):S50–7.
52. Storey KE. A changing landscape: web-based methods for dietary assessment in adolescents. Curr Opin Clin Nutr Metab Care. 2015;**18**(5):437–45.
53. Ekelund U, Tomkinson G, Armstrong N. What proportion of youth are physically active? Measurement issues, levels and recent time trends. Br J Sports Med. 2011;**45**(11):859–65.
54. Prince SA, et al. A comparison of direct versus self-report measures for assessing physical activity in adults: a systematic review. International Journal of Behavioral Nutrition and Physical Activity. 2008;**5**(1):56.
55. Craig CL, et al. International physical activity questionnaire: 12-country reliability and validity. Med Sci Sports Exerc. 2003;**35**(8):1381–95.
56. Crocker PR, et al. Measuring general levels of physical activity: preliminary evidence for the Physical Activity Questionnaire for Older Children. Medicine and science in sports and exercise. 1997;**29**(10):1344–9.
57. Kowalski KC, Crocker PRE, Kowalski NP. Convergent validity of the physical activity questionnaire for adolescents. Pediatric Exercise Science. 1997;**9**(4): 342–52.
58. Biddle SJH, et al. An assessment of self-reported physical activity instruments in young people for population surveillance: Project ALPHA. International Journal of Behavioral Nutrition and Physical Activity. 2011;**8**(1):1.
59. Atkin AJ, et al. Methods of Measurement in epidemiology: sedentary behaviour. International Journal of Epidemiology. 2012;**41**(5):1460–71.
60. Godfrey A, Culhane KM, Lyons GM. Comparison of the performance of the activPAL Professional physical activity logger to a discrete accelerometer-based activity monitor. Med Eng Phys. 2007;**29**(8):930–4.
61. Kozey-Keadle S, et al. Validation of wearable monitors for assessing sedentary behavior. Med Sci Sports Exerc. 2011;**43**(8):1561–7.
62. Grant PM, et al. The validation of a novel activity monitor in the measurement of posture and motion during everyday activities. British Journal of Sports Medicine. 2006;**40**(12):992–7.
63. Dowd KP, Harrington DM, Donnelly AE. Criterion and concurrent validity of the activPAL™ Professional physical activity monitor in adolescent females. PLOS ONE. 2012;**7**(10):e47633.
64. Aminian S, Hinckson EA. Examining the validity of the ActivPAL monitor in measuring posture and ambulatory movement in children. International Journal of Behavioral Nutrition and Physical Activity. 2012;**9**(1):119.
65. Ridley K, Ridgers ND, Salmon J. Criterion validity of the activPAL and ActiGraph for assessing children's sitting and standing time in a school classroom setting. Int J Behav Nutr Phys Act. 2016;**13**:75.
66. Akioyamen LE, et al. Risk factors for cardiovascular disease in heterozygous familial hypercholesterolemia: A systematic review and meta-analysis. J Clin Lipidol. 2018.
67. Rademacher ER, et al. Relation of blood pressure and body mass index during childhood to cardiovascular risk factor levels in young adults. Journal of hypertension. 2009;**27**(9):1766–74.
68. Lamb MM, et al. Association of body fat percentage with lipid concentrations in children and adolescents: United States, 1999–20041–4. The American Journal of Clinical Nutrition. 2011;**94**(3):877–83.
69. Kelley GA, Kelley KS, Tran ZV. Aerobic exercise and lipids and lipoproteins in women: a meta-analysis of randomized controlled trials. J Womens Health (Larchmt). 2004;**13**(10):1148–64.
70. Kelley GA, Kelley KS. Aerobic exercise and lipids and lipoproteins in men: a meta-analysis of randomized controlled trials. J Mens Health Gend. 2006; **3**(1):61–70.
71. Varni JW, Seid M, Kurtin PS. PedsQL 4.0: reliability and validity of the Pediatric Quality of Life Inventory version 4.0 generic core scales in healthy and patient populations. Med Care. 2001;**39**(8):800–12.
72. Varni, J.W. Scaling and scoring of the Pediatric Quality of Life Inventory ™ PedsQL ™. . 2017 [cited 2019 04/0/04]; Available from: http://www.pedsql.org/PedsQL-Scoring.pdf.
73. NICE. Position statement on use of the EQ-5D-5L valuation set for England (updated 2018). 2017 [cited 2019 04/04]; Available from: https://www.nice.org.uk/about/what-we-do/our-programmes/nice-guidance/technology-appraisal-guidance/eq-5d-5l.
74. Greiner W, et al. A single European currency for EQ-5D health states. Results from a six-country study. Eur J Health Econ. 2003;**4**(3):222–31.
75. Foundation, E.R., EQ-5D-3L User Guide Basic information on how to use the EQ-5D-3L instrument. Version 6.0. . 2018.
76. Dolan P. Modeling valuations for EuroQol health states. Med Care. 1997; **35**(11):1095–108.
77. Mente A, et al. Association of dietary nutrients with blood lipids and blood pressure in 18 countries: a cross-sectional analysis from the PURE study. Lancet Diabetes Endocrinol. 2017;**5**(10):774–87.
78. Grundy Scott, M., et al., 2018 AHA/ACC/AACVPR/AAPA/ABC/ACPM/ADA/AGS/APhA/ASPC/NLA/PCNA Guideline on the Management of Blood Cholesterol. Circulation. **0**(0): p. CIR.0000000000000625.
79. Wang Y, Xu D. Effects of aerobic exercise on lipids and lipoproteins. Lipids in Health and Disease. 2017;**16**(1):132.
80. Howie SR. Blood sample volumes in child health research: review of safe limits. Bull World Health Organ. 2011;**89**(1):46–53.
81. Winkler EA, et al. Identifying adults' valid waking wear time by automated estimation in activPAL data collected with a 24 h wear protocol. Physiol Meas. 2016;**37**(10):1653–68.
82. Vickers AJ, Altman DG. Analysing controlled trials with baseline and follow up measurements. BMJ. 2001;323(7321):1123.
83. Lorelli SN, et al. Thematic analysis: striving to meet the trustworthiness criteria. International Journal of Qualitative Methods. 2017;**16**(1): 1609406917733847.
84. Braun V, Clarke V. Using thematic analysis in psychology. Qualitative Research in Psychology. 2006;**3**(2):77–101.

## Publisher's Note

Springer Nature remains neutral with regard to jurisdictional claims in published maps and institutional affiliations.

**Ready to submit your research? Choose BMC and benefit from:**

- fast, convenient online submission
- thorough peer review by experienced researchers in your field
- rapid publication on acceptance
- support for research data, including large and complex data types
- gold Open Access which fosters wider collaboration and increased citations
- maximum visibility for your research: over 100M website views per year

**At BMC, research is always in progress.**

**Learn more** biomedcentral.com/submissions

BMC

For peer review only

BMJ Open

# Reducing cardiovascular disease risk amongst families with familial hypercholesterolaemia by improving diet and physical activity: A randomised controlled feasibility trial

| | |
|---|---|
| Journal: | *BMJ Open* |
| Manuscript ID | bmjopen-2020-044200.R1 |
| Article Type: | Original research |
| Date Submitted by the Author: | 13-Nov-2020 |
| Complete List of Authors: | Kinnear, Fiona; University Hospitals Bristol and Weston NHS Foundation Trust, National Institute for Health Research (NIHR) Bristol Biomedical Research Centre (BRC); University of Bristol<br>Lithander, Fiona; University Hospitals Bristol and Weston NHS Foundation Trust, National Institute for Health Research (NIHR) Bristol Biomedical Research Centre (BRC); University of Bristol, Bristol Medical School<br>Searle, Aidan; University Hospitals Bristol and Weston NHS Foundation Trust, National Institute for Health Research (NIHR) Bristol Biomedical Research Centre (BRC); University of Bristol<br>Bayly, Graham; University Hospitals Bristol and Weston NHS Foundation Trust, Department of Clinical Biochemistry<br>Wei, Christina; St George's University Hospitals NHS Foundation Trust<br>Stensel, David; Loughborough University, National Centre for Sport and Exercise Medicine, School of Sport, Exercise and Health Sciences; University Hospitals of Leicester NHS Trust, National Institute for Health Research (NIHR) Leicester Biomedical Research Centre (BRC)<br>Thackray, Alice; Loughborough University, National Centre for Sport and Exercise Medicine, School of Sport, Exercise and Health Sciences; University Hospitals of Leicester NHS Trust, National Institute for Health Research (NIHR) Leicester Biomedical Research Centre (BRC)<br>Hunt, Linda; University Hospitals Bristol and Weston NHS Foundation Trust, National Institute for Health Research (NIHR) Bristol Biomedical Research Centre (BRC); University of Bristol<br>Shield, Julian; University Hospitals Bristol and Weston NHS Foundation Trust, National Institute for Health Research (NIHR) Bristol Biomedical Research Centre (BRC); University of Bristol |
| <b>Primary Subject Heading</b>: | Diabetes and endocrinology |
| Secondary Subject Heading: | Nutrition and metabolism, Paediatrics, Patient-centred medicine, Qualitative research, Evidence based practice |
| Keywords: | CARDIOLOGY, Lipid disorders < DIABETES & ENDOCRINOLOGY, NUTRITION & DIETETICS, Paediatric endocrinology < DIABETES & ENDOCRINOLOGY, PREVENTIVE MEDICINE, QUALITATIVE RESEARCH |
| | |

SCHOLARONE™
Manuscripts

*I, the Submitting Author has the right to grant and does grant on behalf of all authors of the Work (as defined in the below author licence), an exclusive licence and/or a non-exclusive licence for contributions from authors who are: i) UK Crown employees; ii) where BMJ has agreed a CC-BY licence shall apply, and/or iii) in accordance with the terms applicable for US Federal Government officers or employees acting as part of their official duties; on a worldwide, perpetual, irrevocable, royalty-free basis to BMJ Publishing Group Ltd ("BMJ") its licensees and where the relevant Journal is co-owned by BMJ to the co-owners of the Journal, to publish the Work in this journal and any other BMJ products and to exploit all rights, as set out in our licence.*

*The Submitting Author accepts and understands that any supply made under these terms is made by BMJ to the Submitting Author unless you are acting as an employee on behalf of your employer or a postgraduate student of an affiliated institution which is paying any applicable article publishing charge ("APC") for Open Access articles. Where the Submitting Author wishes to make the Work available on an Open Access basis (and intends to pay the relevant APC), the terms of reuse of such Open Access shall be governed by a Creative Commons licence – details of these licences and which Creative Commons licence will apply to this Work are set out in our licence referred to above.*

*Other than as permitted in any relevant BMJ Author's Self Archiving Policies, I confirm this Work has not been accepted for publication elsewhere, is not being considered for publication elsewhere and does not duplicate material already published. I confirm all authors consent to publication of this Work and authorise the granting of this licence.*

For peer review only

# Reducing cardiovascular disease risk amongst families with familial hypercholesterolaemia by improving diet and physical activity: A randomised controlled feasibility trial

Fiona J Kinnear[1], Fiona E Lithander[1, 2], Aidan Searle[1], Graham Bayly[3], Christina Wei[4], David J Stensel[5,6], Alice E Thackray[5, 6], Linda Hunt[1], Julian P Hamilton-Shield[1].

1. National Institute for Health Research Bristol Biomedical Research Centre, University Hospitals Bristol and Weston NHS Foundation Trust and University of Bristol, Bristol, UK.
2. Bristol Medical School, The University of Bristol, Bristol, UK
3. Department of Clinical Biochemistry, University Hospitals Bristol and Weston NHS Foundation Trust, Bristol, UK
4. St George's University Hospitals NHS Foundation Trust, London, UK
5. National Centre for Sport and Exercise Medicine, School of Sport, Exercise and Health Sciences, Loughborough University, Epinal Way, Loughborough LE11 3TU, UK
6. National Institute for Health Research (NIHR) Leicester Biomedical Research Centre, University Hospitals of Leicester NHS Trust and University of Leicester, Leicester, UK

Corresponding author:
Fiona Kinnear, NIHR Bristol Biomedical Research Centre Nutrition Theme,
University Hospitals Bristol and Weston NHS Foundation Trust and the University of Bristol, Bristol, UK. Email:Fiona.kinnear@bristol.ac.uk

**Word count: 4094**

For peer review only

For peer review only


For peer review only - http://bmjopen.bmj.com/site/about/guidelines.xhtml

## Abstract

**Objective** Familial hypercholesterolaemia (FH) elevates low-density lipoprotein cholesterol (LDL-C) and increases cardiovascular disease (CVD) risk. This study aimed to provide evidence for the feasibility of conducting a randomised controlled trial to evaluate the efficacy of an intervention designed to improve diet and physical activity in families with FH.

**Design** A parallel, randomised, wait-list controlled, feasibility pilot trial.

**Setting** Three outpatient lipid clinics in the UK.

**Participants** Families comprised of children (10-18 years) and their parent with genetically diagnosed FH.

**Intervention** Families were randomised to either 12-week usual-care or intervention. The behavioral change intervention aimed to improve dietary, physical activity and sedentary behaviours. It was delivered to families by dietitians initially via a single face-to face session and then by four telephone or email follow-up sessions.

**Outcome measures** Feasibility was assessed via measures related to recruitment, retention, and intervention fidelity. Post-intervention qualitative interviews were conducted to explore intervention acceptability. Behavioural (dietary intake, physical activity and sedentary time) and clinical (blood pressure, body composition and blood lipids) outcomes were collected at baseline and endpoint assessments to evaluate the intervention's potential benefit.

**Results** Twenty-one families (38% of those approached) were recruited, comprised of 22 children and 17 adults with FH, and 97% of families completed the study. The intervention was implemented with high fidelity and the qualitative data revealed it was well accepted. Between group differences at the endpoint assessment were indicative of the intervention's potential for improving diet in children and adults. Evidence for potential benefits upon physical activity and sedentary behaviours were less apparent. However, the intervention was associated with improvements in several CVD risk factors including LDL-C, with a within group mean decrease of 8% (children) and 10% (adults).

**Conclusions** The study's recruitment, retention, acceptability, and potential efficacy supports the development of a definitive trial, subject to identified refinements.


Trial registration number: ISRCTN24880714, URL: https://doi.org/10.1186/ISRCTN24880714

**Abstract word count: 299**


For peer review only - http://bmjopen.bmj.com/site/about/guidelines.xhtml

## Strengths and limitations of this study

- This is the first study to assess the feasibility and acceptability of delivering a behaviour change intervention, which aims to enable attainment of the dietary and physical activity treatment recommendations, to families affected by familial hypercholesterolaemia (FH).

- The quantitative and qualitative measures employed in this multi-centred, randomised controlled trial have provided evidence of the feasibility and acceptability of conducting a future adequately powered trial, subject to identified refinements.

- An adequately powered, randomised controlled trial is required to formally evaluate the effectiveness of the intervention upon behavioural and clinical outcomes.

- The sample was comprised of mostly highly educated, white European participants and the findings may not be generalisable to all individuals living with FH

For peer review only

## Introduction

Familial hypercholesterolaemia is a genetic disease, characterised by elevated levels of low-density lipoprotein cholesterol (LDL-C) from birth.[1] The disease confers an 18-fold increased risk of cardiovascular disease (CVD), accounting for one in every 17 CVD cases worldwide.[2] The heterozygous genotype of familial hypercholesterolaemia, henceforth called FH, affects one in 311 individuals globally. (2) Pharmacological treatment significantly reduces LDL-C, and thus CVD risk, especially when initiated in childhood.[3] However, CVD risk remains elevated and its onset and severity varies substantially.[4,5]

Environmental factors, including lifestyle behaviours, may explain some of the variance in CVD risk,[6] mediated via LDL-C reductions additive to that attained by pharmacological treatment or effects on other CVD risk factors.[7,8] Both pathways are important as LDL-C treatment goals are often not achieved[9] and hypertension, type II diabetes mellitus (T2DM) and obesity are independently associated with CVD risk amongst adults with FH.[7] Accordingly, adjuvant to pharmacological therapy, clinical guidelines recommend individuals with FH should be encouraged to be physically active, maintain a healthy weight and consume a healthy diet.[10] There is no evidence from randomised controlled trials (RCTs) demonstrating the effectiveness of these recommendations on CVD risk in individuals with FH.[11,12]

A previous RCT sought to address this evidence gap,[13] but the intervention was implemented with poor fidelity and failed to significantly improve dietary or physical activity behaviours.[14] Less than half of children and adults with FH adhere to dietary and physical activity treatment recommendations,[15,16] with several factors identified as barriers including the asymptomatic nature prior to onset of CVD and the perception that it is unnecessary when also taking medication.[17] In order to explore the true effects of diet and physical activity upon CVD risk, it is essential that interventions can successfully change these behaviours. Given the high prevalence of FH, any intervention must also be feasible to implement within the time and resource constraints of health services.[10] Therefore, we previously developed a pragmatic behaviour change intervention specifically designed to address the determinants of dietary and physical


For peer review only - http://bmjopen.bmj.com/site/about/guidelines.xhtml
For peer review only

activity behaviours amongst individuals with FH.[18] As the inheritance pattern of FH means all affected children will have one affected parent, it was delivered to families.

According to the Medical Research Council (U.K.) guidance,[19] healthcare interventions should undergo feasibility testing prior to evaluation in a full scale RCT. Therefore, this trial aimed to provide evidence for the feasibility and acceptability of conducting a future, adequately powered RCT and identify any refinements to the RCT design or intervention required. The six objectives were to:

1. Assess the feasibility of recruiting, randomising, and retaining families with FH
2. Evaluate the feasibility of collecting valid outcome measures
3. Assess the feasibility of implementing the intervention with sufficient fidelity
4. Explore the participant acceptability of the intervention
5. Explore the potential effectiveness of the intervention upon behavioural and clinical outcomes
6. Estimate the sample size required to adequately power a future RCT


For peer review only - http://bmjopen.bmj.com/site/about/guidelines.xhtml

## Methods

The trial received ethical approval from the Cornwall and Plymouth Research Ethics Committee (reference: 18/SW/0121). The study protocol is published,[20] and a CONSORT checklist[21] displayed in supplementary file 1.

### *Trial design and setting*

The study was a parallel, two-arm, randomised, controlled, feasibility pilot trial[22] comparing a diet and physical activity intervention against usual-care waitlist control amongst families living with FH. It was conducted across three National Health Service (NHS) Foundation Trust sites in England, U.K.

### *Participants*

The study aimed to recruit families comprised of a child aged 10-18 years and their parent, both with a genetic diagnosis of FH and established on their current treatment (lifestyle and/or pharmacological) regimen ≥ 1 month. Families could be comprised of multiple affected children and non-affected parents or carers in instances in which affected parents were unwilling, unable or ineligible to participate. If this was not possible, children could participate without parental involvement.

### *Recruitment and randomisation*

All eligible children receiving care from outpatient lipid clinics at the study sites were invited to participate, along with their parent, via invitation letters and/or when attending routine clinic appointments. Written informed consent (or assent and parental consent for children under 16 years old) was obtained from all participants. After baseline data collection, families were randomised to receive usual care and waitlist control or the intervention on a 1:1 basis, stratified by study site. This was carried out by a database manager using pre-prepared, password protected, randomised lists. It was not possible to blind participants or research staff to the randomisation.

### *Sample size*

As this was a feasibility study, a formal sample size calculation was not required.[21] The recruitment of 24 families, based upon the local number of eligible families, was deemed to be large enough to address the study objectives.

For peer review only

***Intervention***

The development and content of the behaviour change intervention are described in detail elsewhere[18] and in supplementary file 2. In brief, the intervention aimed to enable individuals to reduce dietary intakes of total fat, saturated fat and cholesterol; increase intakes of unsaturated fats, fibre, fruits, vegetables and plant stanol or sterol fortified foods; reduce sedentary behaviour and increase physical activity. The Behaviour Change Wheel, which brings together 19 different theories of behaviour change,[23] was applied to the findings of a qualitative evidence synthesis to identify the determinants of treatment behaviours.[17] Twenty-six behavioural change techniques (BCTs) were then incorporated into the intervention to target these.[18] The intervention was delivered to families by one of two dietitians as an initial 1-hour face-to-face session and four email or telephone follow-up sessions over a 12-week intervention period. The dietitians received comprehensive manuals which detailed the content and BCTs to be delivered to families at each session (supplementary file 2).

*Comparator (Waitlist, usual care control)*

Families were informed that they were on a waitlist to receive the intervention at the end of the 12-week study period and received usual care, which comprised of an annual outpatient lipid clinic appointment. Adults, but not children, in both groups had previously received dietetic counseling upon FH diagnosis as part of usual care. No participant received any further dietetic advice during the trial control period.

*Pharmacological treatment*

Participants in both groups were instructed to continue with their prescribed pharmacological treatment, if applicable. Any self-reported changes to medication were documented and validated through confirmation from medical records.

***Outcome measures***

*Feasibility outcomes (study objectives 1-3)*

Recruitment, randomisation and retention rates were collected for families, as the study aimed to recruit children and their parent. Checklists completed by dietitians after each intervention session were analysed to produce outcomes related to the *dose* (number and duration of sessions delivered) and *fidelity* (inclusion of pre-specified content sections and BCTs) of the intervention delivered to each family.[24] Dietitians documented any adaptations made to the intervention. The proportion of participants

For peer review only



For peer review only - http://bmjopen.bmj.com/site/about/guidelines.xhtml

from whom valid outcome measures were collected was used to assess the feasibility of obtaining these measures. While outcome measures were collected from all adults, data is only analysed and presented for adults affected with FH.

*Acceptability outcomes (study objective 4)*

After study completion, 10 families took part in face-to-face or telephone qualitative interviews to explore the acceptability of the intervention. Purposive sampling was employed to yield maximum variation within this sub-sample. The sample included four families from the control group who were interviewed after they received the intervention upon study completion. All interviews were conducted by FJK and were audio-recorded and transcribed *verbatim.*

*Behavioural and clinical outcomes (study objectives 5 & 6)*

It was not appropriate to test the effectiveness hypothesis, as the study was not sufficiently powered to do so.[25] However, all outcome measures to be assessed in a future trial were measured and the potential effectiveness of the intervention on behavioural (diet, physical activity and sedentary behaviours) and clinical (selected CVD risk factors and quality of life) outcomes was explored. Clinical outcomes were measured by the research team at baseline and endpoint assessment visits before and after the 12-week intervention or control period. Behavioural outcomes were measured in the week preceding these visits. A summary is provided below, with full details elsewhere[20] and supplementary file 3.

Participants recorded their dietary intake on four non-consecutive days (including one weekend day) using Intake24, a validated online 24-hour recall tool.[26,27] At least three days were required for analysis and data were analysed to produce outcome measures related to the intervention dietary targets. Participants wore two accelerometers (ActiGraph GT3X+ and activPAL3) for seven consecutive days to measure moderate and vigorous physical activity (MVPA) and time spent being sedentary during waking hours. At least four valid days (including one weekend day) were required for analysis.

Height was measured using a stadiometer and weight (kg), body fat (%) and fat free mass (kg) using bioelectrical impedance scales (Tanita™ MC-780MA). At sites where this equipment was unavailable, weight was measured using medical scales. BMI was



For peer review only - http://bmjopen.bmj.com/site/about/guidelines.xhtml

determined from the height and weight measurements and systolic and diastolic blood pressures were measured using automated oscillometric devices (Dinamap, V100). Health related quality of life (HRQoL) was measured in children using a Pediatric Quality of Life Inventory ™ (PedsQL™) and in adults using the EuroQol Group EQ-5D-3L health questionnaires.[28,29] Fasted blood samples were collected to measure total cholesterol, triglyceride and HDL-C using standard methodology and reagents on local NHS laboratory autoanalysers (Details in supplementary file 3). LDL-C was estimated using the Friedewald equation.[30] The results of lipidomic analyses will be reported in the future, due to the closure of the relevant metabolomics laboratory because of the coronavirus.

***Data analysis***

Feasibility outcomes were analysed descriptively and the qualitative outcomes analysed using the six stages of thematic analysis described by Braun and Clarke.[31] The analysis of the behavioural and clinical outcomes were carried out separately for children and adults. For parametric outcomes, the baseline and endpoint measures for intervention and control groups were summarised with means and standard deviations (S.D.). The endpoint means in the intervention and control groups were compared with and without adjustment for the baseline values using regression analysis,[32] and their differences reported with 95% confidence intervals (C.I.). For non-parametric outcomes, baseline and endpoint measures and within-group pre-to-post changes for intervention and control groups were summarised by median and range. To maximise the power of the models, all valid data collected at baseline and endpoint assessments were included in the analyses.

**Patient involvement**

As detailed in the protocol,[20] patients with FH, under the care of the University Hospitals Bristol NHS Foundation Trust, participated in the development work underpinning this research. They gave feedback about the initial design of the intervention and data collection methods.


For peer review only - http://bmjopen.bmj.com/site/about/guidelines.xhtml

## Results

### *Recruitment, randomisation and retention (study objective 1)*

Recruitment took place between August 2018 and December 2019 and ceased due to study timelines. Recruitment to the trial was 38% of eligible families, with 21 of the target 24 families recruited, 18 (86%) of which comprised of a child and their parent with FH (Figure 1). All families were successfully randomised and retention was high at 97%. The baseline characteristics of the participants (with FH) are displayed in Table 1.

### *Collection of clinical and behavioural outcome measures (study objective 2)*

All families who completed the study attended all research visits, although one child was unable to accompany their parent to the endpoint assessment. Clinical outcome measures were collected from all other participants, with the exception of body composition, due to unavailability of the required equipment at two of the three sites, and blood lipid profile, due to needle phobia (n=2; children), nurse unavailability (n=3; 2 child, 1 adult) and unsuccessful venepuncture (n=2; 1 child, 1 adult). All adults selected the maximum values in the HRQoL questionnaires and due to this ceiling effect, the data were not analysed. Valid paired (baseline and endpoint) dietary outcome measures were obtained from >80% of participants, with missing data due to participants (n=5; 4 child, 1 adult) not completing the required minimum three days of dietary recalls. Due to battery malfunctions (n=12; 6 child, 6 adult) and non-wear due to skin reactions (n=3; children), paired sedentary outcome measures were only collected from 57% and 65% of children and adults, respectively.

### *Intervention implementation and fidelity outcomes (study objective 3)*

All families received the initial intervention session and the duration averaged 66 minutes, 10% higher than the intended 60 minutes. All families received the first three follow-up sessions, only 60% of families opted to receive the fourth follow-up session. Two families chose to receive follow-up sessions via email and eight opted for telephone with an average duration of 25 minutes, which was below the intended 30 minutes. The fidelity to the content and BCTs intended to be delivered in each session was high, with only one content section (‘*barriers and solutions’, detailed in supplementary file 2)* not delivered to 20% of families, due to the initial session exceeding the intended 60 minutes and the family having to leave. In response to the individual needs of the families, four BCTs were not delivered to all families and 80%


For peer review only - http://bmjopen.bmj.com/site/about/guidelines.xhtml

of families received additional BCTs during the follow-up sessions. Two further BCTs were not delivered to 20% of families, as the required information (dietary intake and body composition data) was unavailable for the initial session. Supplementary file 2 provides details of these omitted and additional BCTs.

***Acceptability results (study objective 4)***

Overall the participants were positive towards the content of the intervention, which was perceived to have provided the knowledge and motivation to change behaviours: *'If no-one's telling you what you have to change, then it's harder to think about it.'* (parent). The follow-up sessions were valued as they provided additional information and/or motivational support: *'If we'd not had any contact, it might have been difficult to keep on track.'* (parent). The step-wise approach to goal setting and the family-based delivery of the intervention facilitated behaviour change: *'it's just easier if you have someone else doing it with you.'* (child). If suitable alternatives could be identified, the dietary goals were perceived to be easy to implement and participants intended to continue with the swaps: *'I got used to the cheese and the brown pasta. It just tastes better now than the other pasta.'* (child). Having their child enjoy these foods increased acceptability: *'To see her eating broccoli and green beans, it's what every mum wants.'* (adult). For those who were inactive previously, changing physical activity behaviours proved harder to implement, requiring *'discipline'* (adult) and if they did not find an activity they enjoyed, many continued to struggle. The acceptability of changing behaviours was harder when routines changed during school holidays. However, participants viewed these as short-term lapses and returned to their new habits after: *'The summer holidays have been tricky because you're always out of a routine. When we're back at school it will be easier.'* (adult).

***Behavioural and clinical outcomes (study objectives 5 & 6)***

The baseline data collected from the withdrawn participant was included in the analysis. The results suggest that the intervention may be effective in decreasing intake of total fat, saturated fat and cholesterol, and increasing intake of fibre, fruits and vegetables (Table 2). No participants in either group consumed plant stanol or sterol fortified products at baseline. This remained true for the control group, whereas all but one participant in the intervention group reported consuming a yogurt containing 2g of plant stanols or sterols everyday throughout the intervention. The within-group median change between baseline and endpoint assessment is indicative of a positive effect of



For peer review only - http://bmjopen.bmj.com/site/about/guidelines.xhtml

the intervention upon increasing daily MVPA and decreasing daily sedentary time in adults, but not children (Table 2). However, the data suggests MVPA also increased amongst adults in the control group meaning it is not possible to attribute the improvements solely to the intervention. The direction of trends suggests a favourable effect of the intervention on diastolic blood pressure, total cholesterol and LDL-C in adults and children. (Table 2) Additionally, the within-group median change suggests that the intervention may be associated with improvements in BMI, body composition and HRQoL in children (Table 3).

The within group mean decrease in LDL-C for children in the intervention group was 0.29 (0.58) mmol/l and for adults was 0.33 (0.77) mmol/l, which is an approximate decrease of 8 and 10% from baseline, respectively. A LDL-C reduction of 10% was deemed to be of clinical significance and the sample size calculation was based upon this. Using the mean baseline LDL-C and SDs in children in the intervention group, and a 5% level of significance, a sample size of 42 children in each group was calculated to have 80% statistical power to show a difference between a 10% decrease in LDL-C and no difference in the control group. Using the same methods, a sample size of 68 adults in each group was calculated. To account for non-collection of behavioural outcomes and participation of families with non-affected parents, a future trial should aim to recruit 160 families. Using the 38% recruitment rate, this would require a potential recruitment pool of 420 eligible families.

If multiple affected children from a family were to be recruited to any following trial, an estimate of the intra-class correlation would be needed to calculate an inflation factor for the sample size. Furthermore, mixed models may be needed in the final analyses. As only one family in the present trial included more than one affected child, it was not possible to estimate the intra-class correlation.



For peer review only - http://bmjopen.bmj.com/site/about/guidelines.xhtml

**Discussion**

This trial has demonstrated that it is feasible to recruit, randomise and retain families with FH into a 12-week trial, and to implement and evaluate a dietary and physical activity intervention, which is acceptable to families. While this study was not sufficiently powered, the potentially favourable effects upon CVD risk factors are encouraging and warrant further investigation in a full-scale RCT, subject to the identified refinements.

The 8-10% reduction in LDL-C that that was observed in this trial is clinically significant. It is in fact comparable to that achieved through the addition of further medication, such as one that inhibits the intestinal absorption of cholesterol, to standard statin therapy.[33] Many children and adults with FH do not meet LDL-C treatment targets, even when using maximally licensed doses of medication.[3] There is no LDL-C concentration below which the beneficial effects upon CVD risk cease to improve,[34] and a 'the lower, the better' approach is advocated.[10] Any additional LDL-C reduction that can be achieved through non-pharmacological approaches is critically important, highlighting the potential benefit of the LDL-C finding.

Most families in this trial were recruited at paediatric lipid clinics, held every three to six months. This infrequency contributed to the recruitment target not being met within study timelines, a common finding in RCTs.[35] The infrequent clinics are representative of the low number of individuals diagnosed with FH in the U.K., (8%) and worldwide (1-15%).[36] Ongoing efforts to increase the detection of FH through cascade testing continue to improve these statistics,[36] which should increase the number of eligible families and clinics in a future trial. The study recruited 38% of eligible families, higher than the 34% reported in a similar study conducted in adults with FH[37] and the 22% reported in a family-based study to reduce CVD risk in children without FH.[38] Recruitment could be improved by telephoning non-responders[39] and incorporating a research visit into a lipid clinic appointment to reduce the time burden on participants, a common barrier to participation in this study. Once recruited, the acceptability of the trial was high regardless of randomisation, as reflected by retention of 97%. This study has also demonstrated the feasibility of non-affected parents participating in the trial when the affected parent cannot. As treatment is most beneficial when initiated in childhood,[10] and the family-based delivery of the intervention was integral to the



For peer review only - http://bmjopen.bmj.com/site/about/guidelines.xhtml

participants' acceptability, non-affected parents should continue to be eligible to facilitate recruitment of children in a future trial.

Participant acceptability and compliance with recording dietary and physical activity behaviours was higher than previous research.[40, 41] Battery malfunction due to the age of the accelerometers used, accounted for most missing sedentary behaviour outcomes and a future trial should use new or recently refurbished activPALs. The low collection of body composition outcomes can be overcome in a future trial by providing sites with the equipment and the updated EQ-5D-5L version of the adult QoL questionnaire should be used, which reduces the occurrence of the ceiling effect found in this study.[42]

In contrast to a previous trial conducted in adults with FH,[14] the intervention was implemented with high fidelity, likely due to the comprehensive manuals created. The adaptations made were almost all in response to the individual needs of families. If adaptations do not undermine the mechanisms of action by which the intervention is proposed to have its effect, tailoring interventions to suit different contexts can have beneficial effects upon the outcomes.[43] As the intervention was systematically developed,[18] the effect of adaptations can be evaluated in a definitive trial.[21]

Participants' dietary intakes fell short of treatment recommendations at baseline, as reported by others previously.[13,44] This was despite all adults having previously received dietetic advice upon diagnosis, suggesting the specifically designed behaviour change intervention has advantages over routine advice provided in outpatient lipid clinics. The qualitative findings serve to demonstrate that the intervention was highly acceptable to families with FH, who were able and willing to change their lifestyle behaviours. This was largely driven by the family-based approach, also adopted by a previous non-randomised trial, which successfully improved dietary behaviours of children with FH.[44] However, only the behaviours of the children were targeted and eight dietetic sessions were provided.[44] While not powered to detect changes, the findings suggest that the pragmatic intervention evaluated in the current study may achieve dietary improvements in both adults and children without the need for multiple dietetic sessions.

Unlike previous research,[14] the results suggest physical activity and sedentary behaviours of adults with FH may be improved by the intervention developed for the


For peer review only - http://bmjopen.bmj.com/site/about/guidelines.xhtml

current study. However, trends towards improvement amongst adults in the control group were also observed. This finding may be explained by the *reactivity bias* associated with wearing accelerometers, in which participants modify their habitual physical activity in response to wearing accelerometers.[45] In contrast, the results of this study do not suggest that physical activity or sedentary behaviours were improved amongst children in either group. Participants received feedback about the disparities between their dietary intakes and the recommendations, but not for physical activity. Parents of inactive children typically over-estimate the time they spend in physical activity, as do the children themselves.[46] Given the low rates of children meeting the physical activity recommendations in this study, feedback on current physical activity levels in comparison to the recommendations should be provided in a future trial to increase motivation to change this behaviour.

***Strengths and limitations***

The participants were highly educated and mostly white Europeans, limiting the generalisability of the findings to all families with FH, a common condition affecting all ethnicities.[2] The qualitative sample did not include any participants of different ethnicities and it cannot be concluded that data saturation was achieved, limiting the validity of the qualitative findings.[47] The 12-week follow-up period in this study does not enable exploration of longer-term effects, which are of importance to treatment for a life-long condition like FH.[11] Individuals attend annual lipid clinic appointments and it would be feasible to extend the follow-up period to 12 months in a future trial. The dietary intake estimates in this study are limited by the misreporting bias associated with all self-report methods of dietary assessment.[48] A future trial should include objective measures of dietary intakes to validate the estimates of the nutrients targeted by the intervention, such as blood concentrations of fatty acids and phytosterols.[49] Adherence to pharmacological treatment influences LDL-C concentrations and this was not formally assessed in this study. The validated Morisky Eight-item Medication Adherence scale is suggested for a future trial.[50]



For peer review only - http://bmjopen.bmj.com/site/about/guidelines.xhtml

**Conclusion**

This study has addressed the main uncertainties ahead of a definitive trial and if the identified refinements are made to the study design then a fully powered RCT is feasible and warranted. Such a trial will address the unanswered question of the potential benefits of the current diet and physical activity treatment recommendations upon LDL-C treatment goals and CVD risk in children and adults with FH.

For peer review only

**Acknowledgements** We would like to thank Dr Coxson, Dr Edmonds and all members of the research teams at University Hospitals Bristol and Weston, St. George's University Hospitals and Royal United Hospitals Bath NHS Foundation Trusts. DJS and AET acknowledge support from the National Institute for Health Research NIHR Leicester Biomedical Research Centre. The views expressed are those of the authors and not necessarily those of the NHS, the NIHR or the Department of Health and Social Care.

**Contributors:** FJK, JHS, FEL, GB, CW and DS contributed to the design of the study. FJK and AS analysed and interpreted the qualitative data. FJK, AET, FEL, JHS analysed and interpreted the quantitative data. FJK and LH devised and conducted the statistical analysis plan for this analysis. FJK prepared the manuscript, which was critically revised by all co-authors and all authors approved the final version.

**Funding** This study was funded by the NIHR Biomedical Research Centre at University Hospitals Bristol and Weston NHS Foundation Trust and the University of Bristol. Award/grant number: not applicable. The views expressed are those of the authors and not necessarily those of the NIHR or the Department of Health and Social Care.

**Competing interests** All authors declare that they have no competing interests.

**Data sharing statement:** Data from this study are available on request.

**Figure legends**

Figure 1. Consolidated Standards of Reporting Trials (CONSORT) diagram of recruitment of families across the three sites.

**Supplementary files**

Supplementary file 1. CONSORT 2010 checklist of information to include when reporting a pilot or feasibility trial

Supplementary file 2. Additional details of intervention content and delivery

Supplemental file 3. Additional details of outcome measures used in study

For peer review only



For peer review only - http://bmjopen.bmj.com/site/about/guidelines.xhtml

# Tables

## Table 1 Baseline characteristics of the study participants*

| Characteristic | Adults | | Children | |
|---|---|---|---|---|
| | Control (n=9) | Intervention (n=8) | Control (n=12) | Intervention (n=10) |
| **Gender- n female (%)** | 4 (44%) | 4 (50%) | 6 (50%) | 5 (50%) |
| **Age (years)- mean (SD); range** | 51 (5); 43-59 | 42 (6); 32-49 | 15 (3) 10-18 | 13 (2); 10-16 |
| **Ethnicity- n (%)** | | | | |
| *White European* | 8 (89%) | 7 (88%) | 9 (75%) | 9 (90%) |
| *Asian* | 1 (11%) | 1 (12%) | 3 (25%) | 1 (10%) |
| **Managerial or professional job- n (%)** | 7 (78%) | 8 (100%) | - | - |
| **Treatment- n (%)** | | | | |
| *Lifestyle only* | 0 | 1 (13%) | 2 (17%) | 3 (30%) |
| *Statin medication* | 2 (22%) | 2 (25%) | 10 (80%) | 7 (70%) |
| *Statin and Ezetimibe medication* | 6 (67%) | 5 (63%) | 0 | 0 |
| *PCSK9 inhibitor injections* | 1 (11%) | 0 | 0 | 0 |
| **FH pathogenic variant location- n (%)** | | | | |
| *LDLR* | 7 (78%) | 5 (63%) | 10 (83%) | 7 (70%) |
| *APOB* | 2 (12%) | 2 (25%) | 2 (17%) | 2 (20%) |
| *PCSK9* | 0 | 1 (12%) | 0 | 1 (10%) |
| **Risk factors – n (%)** | | | | |
| *Current smoker* | 0 | 1 (13%) | 0 | 0 |
| *Previous smoker* | 4 (44%) | 1 (13%) | 0 | 0 |
| *Hypertension* | 2 (22%) | 1 (13%) | 0 | 0 |
| *Overweight weight status†* | 4 (44%) | 4 (50%) | 2 (17%) | 2 (20%) |
| *Obese weight status ‡* | 0 | 0 | 1 (8%) | 1 (10%) |

N, number; SD, standard deviation; LDLR, low density lipoprotein receptor; APOB, Apolipoprotein B; PCSK9, Proprotein convertase subtilisin/kexin type 9

*Only adults with FH included in this table

† Overweight in adults defined as BMI ≥ 25-29.9kg/m² and BMI centile ≥ 91st percentile for children

‡ Obese defined as BMI ≥ 30kg/m² for adults and BMI centile ≥ 98th percentile for children

For peer review only



For peer review only - http://bmjopen.bmj.com/site/about/guidelines.xhtml

Table 2. Mean daily intakes of nutrients and clinical outcome measures (SBP, DBP, total cholesterol, LDL-C, HDL-C and HRQoL) at baseline and endpoint assessments for control and intervention groups and a comparison of the differences between groups at the endpoint assessment unadjusted and adjusted for baseline values*

| | | **Children** | | | | | | | **Adults** | | | | | | |
|---|---|---|---|---|---|---|---|---|---|---|---|---|---|---|---|
| Outcome | **Group** | **N** | **Baseline** | **N** | **Endpoint** | Endpoint difference (95% CI) | N | Adujsted difference† (95% CI) | **N** | **Baseline** | **N** | **Endpoint** | Endpoint difference (95% CI) | N | Adujsted difference† (95% CI) |
| Fat intake (% TEI) | Intervention | 9 | 33.0 (6.4) | 9 | 28.3 (5.1) | -5.6 | 9 | -5.3 | 8 | 35.4 (7.8) | 8 | 31.2 (5.9) | -0.3 | 8 | -2.3 |
| | Control | 10 | 32.9 (4.8) | 9 | 33.9 (4.1) | (-10.2 to -0.9) | 8 | (-8.9 to -1.5) | 9 | 32.0 (5.7) | 8 | 31.6 (6.3) | (-6.9 to 6.2) | 8 | (-7.4 to 2.9) |
| SFA intake (%TEI) | Intervention | 9 | 12.6 (2.0) | 9 | 10.3 (2.6) | -1.8 | 9 | -1.8 | 8 | 12.1(3.8) | 8 | 9.9 (2.6) | -1.1 | 8 | -1.9 |
| | Control | 10 | 12.1 (2.9) | 9 | 12.1 (2.1) | (-4.2 to 0.5) | 8 | (-4.3 to 0.8) | 9 | 10.8 (3.4) | 8 | 10.9 (3.3) | (-4.3 to 2.2) | 8 | (-4.3 to 0.5) |
| MUFA intake (%TEI) | Intervention | 9 | 11.6 (4.1) | 9 | 9.4 (2.0) | -2.8 | 9 | -3.2 | 8 | 13.5 (3.7) | 8 | 11.5 (3.4) | 0.9 | 8 | 0.3 |
| | Control | 10 | 11.3 (1.8) | 9 | 12.2 (2.3) | (-4.9 to -0.6) | 8 | (-5.3 to -1.01) | 9 | 12.1 (2.6) | 8 | 10.6 (3.5) | (-2.8 to 4.6) | 8 | (-3.3 to 3.9) |
| PUFA intake (%TEI) | Intervention | 9 | 4.8 (1.4) | 9 | 4.8(1.1) | -0.4 | 9 | -0.6 | 8 | 5.7 (1.5) | 8 | 5.6 (1.6) | 0.1 | 8 | 0.03 |
| | Control | 10 | 4.6 (1.1) | 9 | 5.3 (1.3) | (-1.7 to 0.8) | 8 | (-1.9 to 0.7) | 9 | 5.2 (1.4) | 8 | 5.5 (2.2) | (-1.9 to 2.2) | 8 | (-2.1 to 2.2) |
| Cholesterol intake (mg) | Intervention | 9 | 181.9 (71.2) | 9 | 149.4 (54.6) | -34.1 | 9 | -24.1 | 8 | 219.2 (84.6) | 8 | 204.4 (30.5) | 63.9 | 8 | 28.1 |
| | Control | 10 | 220.9 (95.2) | 9 | 183.5 (34.7) | (-117.2 to 48.7) | 8 | (-100.9 to 52.7) | 9 | 160.9 (60.1) | 8 | 37.4 (105.8) | (-39.6 to 167.5) | 8 | (-76.6 to 132.8) |
| Fibre intake (g) | Intervention | 9 | 20.0 (5.2) | 9 | 24.2 (6.4) | 7.8 | 9 | 5.2 | 8 | 15.5 (5.7) | 8 | 20.6 (7.8) | -2.1 | 8 | 1.1 |
| | Control | 10 | 15.4 (4.0) | 9 | 16.3 (5.7) | (1.8 to 13.9) | 8 | (-0.7 to 10.9) | 9 | 22.4 (10.6) | 8 | 22.6 (7.4) | (-10.2 to 6.1) | 8 | (-6.7 to 8.8) |
| Fruit & vegetable intake (portions) | Intervention | 9 | 3.2 (1.7) | 9 | 4.2 (0.9) | 2.2 | 9 | 2.2 | 8 | 3.3 (2.0) | 8 | 4.1 (1.5) | 1.2 | 8 | 1.6 |
| | Control | 10 | 2.2 (1.1) | 9 | 2.0 (0.6) | (1.4 to 3.0) | 8 | (1.2 to 3.2) | 9 | 4.5 (1.8) | 8 | 2.9 (1.3) | (-0.3 to 2.8) | 8 | (0.1 to 3.1) |
| SBP (mmHg) | Intervention | 10 | 104.5 (10.5) | 9 | 104.9 (6.3) | -3.1 | 9 | -1.6 | 8 | 115.5 (15.1) | 8 | 111.9 (3.6) | -8.1 | 8 | -6.8 |
| | Control | 12 | 110.3 (102) | 11 | 107.9 (8.3) | (-10.9 to 4.8) | 11 | (-8.6 to 5.3) | 9 | 117.5 (14.5) | 9 | 120 (4.5) | (-21.2 to 4.9) | 9 | (-15.9 to 1.4 |
| DBP (mmHg) | Intervention | 10 | 62.9 (6.7) | 9 | 58.9 (4.9) | -4.1 | 9 | -2.2 | 8 | 75.4 (10.1) | 8 | 71.6 (7.2) | -3.2 | 8 | -6.8 |
| | Control | 12 | 68.9 (9.2) | 11 | 63.0 (2.4) | (-10.4 to 2.3) | 11 | (-8.1 to 3.7) | 9 | 69.7 (8.6) | 9 | 74.8 (8.6) | (-11.4 to 5.1) | 9 | (-12.7 to -0.9) |
| Total cholesterol (mmol/l) | Intervention | 8 | 5.2 (1.9) | 8 | 5.0 (1.7) | -0.06 | 8 | -0.3 | 7 | 5.0 (0.9) | 8 | 4.8 (0.3) | -0.08 | 7 | -0.4 |
| | Control | 10 | 4.8 (0.8) | 8 | 4.9 (0.8) | (-1.4 to 1.5) | 8 | (-0.9 to 0.4) | 9 | 4.6 (0.9) | 8 | 4.9 (0.5) | (-1.3 to 1.2) | 8 | (-1.2 to 0.4) |
| LDL-C (mmol/l) | Intervention | 8 | 3.5 (0.8) | 8 | 3.2 (1.6) | -0.2 | 8 | -0.36 | 7 | 3.2 (0.7) | 8 | 2.8 (0.8) | -0.2 | 7 | -0.56 |
| | Control | 10 | 3.2 (0.8) | 8 | 3.3 (0.7 | (-1.5 to 1.1) | 8 | (-0.82 to 0.12) | 9 | 2.7 (0.8) | 8 | 2.9 (1.1) | (-1.2 to 0.8) | 8 | (-1.33 to 0.12) |
| HDL-C (mmol/l) | Intervention | 8 | 1.4 (0.3) | 8 | 1.4 (0.3) | 0.1 | 8 | 0.01 | 7 | 1.47 (0.16) | 8 | 1.39 (0.22) | -0.13 | 7 | -0.17 |
| | Control | 10 | 1.2 (0.3) | 8 | 1.3 (0.1 | (-0.2 to 0.4) | 8 | (-0.1 to 0.2) | 9 | 1.45 (0.27) | 8 | 1.52 (0.09) | (-0.38 to 0.13) | 8 | (-0.38 to 0.04) |
| HRQoL ‡ (Score 0 to 100) | Intervention | 10 | 85.1 (9.8) | 9 | 89.0 (7.8) | 16.7 | 9 | 5.9 | | | | | | | |
| | Control | 12 | 73.4 (9.9) | 11 | 72.3 (3.3) | (7.6 to 25.7) | 11 | (-0.3 to 12.0) | | | | | | | |

N, number; CI, confidence interval; TEI, total energy intake; SFA, saturated fatty acids; MUFA, monounsaturated fatty acids; PUFA, polyunsaturated fatty acids; SBP, systolic blood pressure; DBP, diastolic blood pressure; LDL-C, low density lipoprotein cholesterol; HDL-C, high density lipoprotein; HRQoL, health related quality of life

*All data has been included, the ‘N’ columns indicate the number of participants included in each estimated group mean value and estimated mean differences between groups.

† Adjusted for baseline values, this difference estimate includes only participants for whom paired baseline and endpoint data was collected indicated in the ’N’ column

‡ HRQoL data presented for children only due to ‘ceiling effect’ that occurred in the data collected from adult participants

For peer review only



For peer review only - http://bmjopen.bmj.com/site/about/guidelines.xhtml

Table 3. Median (range) values for BMI, body composition, daily MVPA and sedentary time at baseline and endpoint assessments for control and intervention groups and the median change (range) between these timepoints for both groups for children and adults*†

| | | Children | | | | | | Adults | | | | | |
|---|---|---|---|---|---|---|---|---|---|---|---|---|---|
| Outcomes | Group | N | **Baseline median (range)** | N | **Endpoint median (range)** | N | **Median change (range)** | N | **Baseline median (range)** | N | **Endpoint median (range)** | N | **Median change (range)** |
| BMI (z-score children; kg/m$^2$ adults) | Intervention | 10 | 0.71 (-1.46 to 3.14) | 9 | 0.54 (-1.74 to 3.18) | 9 | -0.20 (-0.39 to 0.07) | 8 | 24.9 (21.9 to 29.1) | 8 | 25.5 (22.2 to 28.9) | 8 | 0.1 (-1.31 to 0.68) |
| | Control | 12 | 0.41 (-1.79 to 2.35) | 11 | 0.31 (-1.72 to 1.45) | 11 | 0.07 (-0.28 to 0.30) | 9 | 24.0 (21.7 to 28.1) | 9 | 23.2 (22.5 to 27.5) | 9 | -0.41 (-1.85 to 1.35) |
| Body fat (%) | Intervention | 6 | 21.2 (13 to 24.5) | 5 | 22.4 (12.9 to 23.7) | 5 | -0.1 (-1.0 to 0.9) | 4 | 26.4 (23 to 36.5) | 4 | 26.8 (21.6 to 33.7) | 4 | -0.6 (-2.8 to 0.5) |
| | Control | 6 | 27.9 (14.4 to 37) | 5 | 23.8 (16.3 to 29.2) | 5 | 0.2 (-1.4 to 0.3) | 4 | 29.2 (22.4 to 33.2) | 4 | 26.9 (22.7 to 32.5) | 4 | -0.9 (-3.5 to 0.3) |
| Fat free mass (kg) | Intervention | 6 | 41.3 (25.4 to 56.9) | 5 | 43.1 (29.4 to 56.2) | 5 | -0.6 (-1.0 to -0.3) | 4 | 45.1 (41.8 to 52.9) | 4 | 44.9 (40.8 to 54.4) | 4 | -0.2 (-1 to 1.5) |
| | Control | 6 | 38.2 (27.2 to 43.8) | 5 | 39.1 (29.2 to 47.3) | 5 | 1.6 (0.3 to 3.5) | 4 | 52.4 (40.7 to 58.2) | 4 | 51.8 (40.6 to 58) | 4 | -0.2 (-2.2 to 0.9) |
| Daily MVPA (minutes) | Intervention | 10 | 67.1 (59.9 to 84.6) | 10 | 62.2 (42.2 to 86.2) | 10 | -4.2 (-14.3 to 2.4) | 8 | 37.7 (28.0 to 63.1) | 8 | 47.4 (27.9 to 79.9) | 8 | 4.3 (1.2 to 17.2) |
| | Control | 10 | 51.5 (41.9 to 71.9) | 8 | 45.9 (38.7 to 59.4) | 6 | -5.9 (-19.1 to 12.4) | 8 | 34.5 (21.4 to 59.0) | 8 | 41.9 (24.7 to 65.9) | 7 | 6 (-22.2 to 19.1) |
| Daily sedentary time (minutes) | Intervention | 9 | 551 (497 to 553) | 8 | 560 (526 to 608) | 7 | 14 (-41 to 105) | 8 | 593 (543 to 624) | 6 | 552 (529 to 583) | 6 | -34 (-52 to -7) |
| | Control | 10 | 515 (494 to 633) | 6 | 595 (532 to 674) | 5 | 81 (10 to 160) | 8 | 571 (478 to 619) | 8 | 537 (293 to 679) | 5 | 3.8 (-32 to 19) |

N, number; BMI, body mass index; MVPA, moderate and vigorous physical activity.
*All data has been included, the 'N' columns indicate the number of participants included in each estimated group median value and estimated median within group changes from baseline to endpoint
† Ranges are presented due to small sample sizes.

For peer review only



For peer review only - http://bmjopen.bmj.com/site/about/guidelines.xhtml

## References

1. Goldstein JH, HH; Brown, MS. Familial hypercholesterolemia. In: Sciver CR A, Sly WS, Valle D. editors, editor. The Metabolic and Molecular Bases of Inherited Disease. 8th edition ed. New York: McGraw-Hill; 2001. p. 2863-913.
2. Beheshti SO, Madsen CM, Varbo A, Nordestgaard BG. Worldwide Prevalence of Familial Hypercholesterolemia: Meta-Analyses of 11 Million Subjects. Journal of the American College of Cardiology. 2020;75(20):2553-66.
3. Alonso R, Perez de Isla L, Muñiz-Grijalvo O, Mata P. Barriers to Early Diagnosis and Treatment of Familial Hypercholesterolemia: Current Perspectives on Improving Patient Care. Vasc Health Risk Manag. 2020;16:11-25.
4. Bianconi V, Banach M, Pirro M. Why patients with familial hypercholesterolemia are at high cardiovascular risk? Beyond LDL-C levels. Trends Cardiovasc Med. 2020.
5. Galema-Boers AM, Lenzen MJ, Engelkes SR, Sijbrands EJ, Roeters van Lennep JE. Cardiovascular risk in patients with familial hypercholesterolemia using optimal lipid-lowering therapy. Journal of Clinical Lipidology. 2018;12(2):409-16.
6. Arsenault BJ, Perrot N, Couture P. Does lifestyle contribute to disease severity in patients with inherited lipid disorders? Current opinion in lipidology. 2017;28(2):177-85.
7. Akioyamen LE, Genest J, Chu A, Inibhunu H, Ko DT, Tu JV. Risk factors for cardiovascular disease in heterozygous familial hypercholesterolemia: A systematic review and meta-analysis. J Clin Lipidol. 2018.
8. Mach F, Baigent C, Catapano AL, Koskinas KC, Casula M, Badimon L, et al. 2019 ESC/EAS Guidelines for the management of dyslipidaemias: lipid modification to reduce cardiovascular risk: The Task Force for the management of dyslipidaemias of the European Society of Cardiology (ESC) and European Atherosclerosis Society (EAS). European heart journal. 2019;41(1):111-88.
9. Stock J. First insights from the EAS familial hypercholesterolaemia collaboration registry: FH is still underdiagnosed and undertreated. Atherosclerosis. 2019;290:138-9.
10. Watts GF, Gidding SS, Mata P, Pang J, Sullivan DR, Yamashita S, et al. Familial hypercholesterolaemia: evolving knowledge for designing adaptive models of care. Nat Rev Cardiol. 2020;17(6):360-77.
11. Malhotra A, Shafiq N, Arora A, Singh M, Kumar R, Malhotra S. Dietary interventions (plant sterols, stanols, omega-3 fatty acids, soy protein and dietary fibers) for familial hypercholesterolaemia. Cochrane Database Syst Rev. 2014(6):CD001918.
12. Lozano P, Henrikson NB, Dunn J, Morrison CC, Nguyen M, Blasi PR, et al. U.S. Preventive Services Task Force Evidence Syntheses, formerly Systematic Evidence Reviews. Lipid Screening in Childhood and Adolescence for Detection of Familial Hypercholesterolemia: A Systematic Evidence Review for the US Preventive Services Task Force. Rockville (MD): Agency for Healthcare Research and Quality (US); 2016.
13. Broekhuizen K, van Poppel MN, Koppes LL, Kindt I, Brug J, van Mechelen W. No significant improvement of cardiovascular disease risk indicators by a lifestyle intervention in people with familial hypercholesterolemia compared to usual care: results of a randomised controlled trial. BMC research notes. 2012;5:181.
14. Broekhuizen K, Jelsma JGM, van Poppel MNM, Koppes LLJ, Brug J, van Mechelen W. Is the process of delivery of an individually tailored lifestyle intervention associated with improvements in LDL cholesterol and multiple lifestyle behaviours in people with familial hypercholesterolemia? BMC public health [Internet]. 2012 2012; 12:[348 p.].
15. Avis HJ, Kusters DM, Vissers MN, Huijgen R, Janssen TH, Wiegman A, et al. Follow-Up of Children Diagnosed with Familial Hypercholesterolemia in a National Genetic Screening Program. The Journal of Pediatrics. 2012;161(1):99-103.


For peer review only - http://bmjopen.bmj.com/site/about/guidelines.xhtml

16. Claassen L, Henneman L, Kindt I, Marteau TM, Timmermans DRM. Perceived Risk and Representations of Cardiovascular Disease and Preventive Behaviour in People Diagnosed with Familial Hypercholesterolemia: A Cross-sectional Questionnaire Study. Journal of Health Psychology. 2010;15(1):33-43.
17. Kinnear FJ, Wainwright E, Perry R, Lithander FE, Bayly G, Huntley A, et al. Enablers and barriers to treatment adherence in heterozygous familial hypercholesterolaemia: a qualitative evidence synthesis. BMJ Open. 2019;9(7):e030290.
18. Kinnear FJ, Wainwright E, Bourne JE, Lithander FE, Hamilton-Shield J, Searle A. The development of a theory informed behaviour change intervention to improve adherence to dietary and physical activity treatment guidelines in individuals with familial hypercholesterolaemia (FH). BMC health services research. 2020;20(1):27.
19. Craig P, Dieppe P, Macintyre S, Michie S, Nazareth I, Petticrew M. Developing and evaluating complex interventions: the new Medical Research Council guidance. BMJ. 2008;337.
20. Kinnear FJ, Hamilton-Shield JP, Stensel DJ, Bayly G, Searle A, Thackray AE, et al. Nutrition and physical activity intervention for families with familial hypercholesterolaemia: protocol for a pilot randomised controlled feasibility study. Pilot Feasibility Stud. 2020;6:42.
21. Eldridge SM, Chan CL, Campbell MJ, Bond CM, Hopewell S, Thabane L, et al. CONSORT 2010 statement: extension to randomised pilot and feasibility trials. BMJ. 2016;355:i5239.
22. Eldridge SM, Lancaster GA, Campbell MJ, Thabane L, Hopewell S, Coleman CL, et al. Defining Feasibility and Pilot Studies in Preparation for Randomised Controlled Trials: Development of a Conceptual Framework. PloS one. 2016;11(3):e0150205-e.
23. Michie S, van Stralen MM, West R. The behaviour change wheel: A new method for characterising and designing behaviour change interventions. Implementation Science. 2011;6(1):42.
24. Moore GF, Audrey S, Barker M, Bond L, Bonell C, Hardeman W, et al. Process evaluation of complex interventions: Medical Research Council guidance. BMJ : British Medical Journal. 2015;350:h1258.
25. Sim J. Should treatment effects be estimated in pilot and feasibility studies? Pilot and Feasibility Studies. 2019;5(1):107.
26. Simpson E, Bradley J, Poliakov I, Jackson D, Olivier P, Adamson AJ, et al. Iterative Development of an Online Dietary Recall Tool: INTAKE24. Nutrients. 2017;9(2):118.
27. Bradley J, Simpson E, Poliakov I, Matthews J, Olivier P, Adamson A, et al. Comparison of INTAKE24 (an Online 24-h Dietary Recall Tool) with Interviewer-Led 24-h Recall in 11–24 Year-Old. Nutrients. 2016;8(6):358.
28. Rabin R, de Charro F. EQ-5D: a measure of health status from the EuroQol Group. Ann Med. 2001;33(5):337-43.
29. EuroQoL Research Foundation. EQ-5D-3L User Guide Basic information on how to use the EQ-5D-3L instrument. Version 6.0. . 2018.
30. Friedewald WT, Levy RI, Fredrickson DS. Estimation of the concentration of low-density lipoprotein cholesterol in plasma, without use of the preparative ultracentrifuge. Clin Chem. 1972;18(6):499-502.
31. Braun V, Clarke V. Using thematic analysis in psychology. Qualitative Research in Psychology. 2006;3(2):77-101.
32. Vickers AJ, Altman DG. Analysing controlled trials with baseline and follow up measurements. BMJ. 2001;323(7321):1123.
33. Pisciotta L, Fasano T, Bellocchio A, Bocchi L, Sallo R, Fresa R, et al. Effect of ezetimibe coadministered with statins in genotype-confirmed heterozygous FH patients. Atherosclerosis. 2007;194(2):e116-e22.


For peer review only - http://bmjopen.bmj.com/site/about/guidelines.xhtml

34. Ference BA, Ginsberg HN, Graham I, Ray KK, Packard CJ, Bruckert E, et al. Low-density lipoproteins cause atherosclerotic cardiovascular disease. 1. Evidence from genetic, epidemiologic, and clinical studies. A consensus statement from the European Atherosclerosis Society Consensus Panel. European heart journal. 2017;38(32):2459-72.
35. McDonald AM, Knight RC, Campbell MK, Entwistle VA, Grant AM, Cook JA, et al. What influences recruitment to randomised controlled trials? A review of trials funded by two UK funding agencies. Trials. 2006;7(1):9.
36. Vallejo-Vaz AJ, De Marco M, Stevens CAT, Akram A, Freiberger T, Hovingh GK, et al. Overview of the current status of familial hypercholesterolaemia care in over 60 countries - The EAS Familial Hypercholesterolaemia Studies Collaboration (FHSC). Atherosclerosis. 2018;277:234-55.
37. Broekhuizen K, van Poppel MN, Koppes LL, Kindt I, Brug J, van Mechelen W. Can multiple lifestyle behaviours be improved in people with familial hypercholesterolemia? Results of a parallel randomised controlled trial. PloS one. 2012;7(12):e50032.
38. Schwandt P, Geiß HC, Ritter MM, Üblacker C, Parhofer KG, Otto C, et al. The Prevention Education Program (PEP). A Prospective Study of the Efficacy of Family-Oriented Life Style Modification in the Reduction of Cardiovascular Risk and Disease: Design and Baseline Data. Journal of Clinical Epidemiology. 1999;52(8):791-800.
39. Treweek S, Lockhart P, Pitkethly M, Cook JA, Kjeldstrøm M, Johansen M, et al. Methods to improve recruitment to randomised controlled trials: Cochrane systematic review and meta-analysis. BMJ Open. 2013;3(2):e002360.
40. Howie EK, Straker LM. Rates of attrition, non-compliance and missingness in randomized controlled trials of child physical activity interventions using accelerometers: A brief methodological review. Journal of Science and Medicine in Sport. 2016;19(10):830-6.
41. Rowland MK, Adamson AJ, Poliakov I, Bradley J, Simpson E, Olivier P, et al. Field Testing of the Use of Intake24-An Online 24-Hour Dietary Recall System. Nutrients. 2018;10(11):1690.
42. Janssen MF, Pickard AS, Golicki D, Gudex C, Niewada M, Scalone L, et al. Measurement properties of the EQ-5D-5L compared to the EQ-5D-3L across eight patient groups: a multi-country study. Quality of Life Research. 2013;22(7):1717-27.
43. Hawe P, Shiell A, Riley T. Complex interventions: how "out of control" can a randomised controlled trial be? Bmj. 2004;328(7455):1561-3.
44. Rodriguez-Borjabad C, Malo AI, Ibarretxe D, Girona J, Heras M, Ferre R, et al. Efficacy of therapeutic lifestyle changes on lipid profiles assessed by NMR in children with familial and non-familial hypercholesterolemia. Clin Investig Arterioscler. 2020;32(2):49-58.
45. Sylvia LG, Bernstein EE, Hubbard JL, Keating L, Anderson EJ. Practical guide to measuring physical activity. Journal of the Academy of Nutrition and Dietetics. 2014;114(2):199-208.
46. Corder K, van Sluijs EMF, McMinn AM, Ekelund U, Cassidy A, Griffin SJ. Perception Versus Reality: Awareness of Physical Activity Levels of British Children. American journal of preventive medicine. 2010;38(1):1-8.
47. Fusch PIan, L.R. Are we there yet? Data saturation in qualitative research. The Qualitative Report. 2015;20(9):1408-16.
48. Cade JE, Warthon-Medina M, Albar S, Alwan NA, Ness A, Roe M, et al. DIET@NET: Best Practice Guidelines for dietary assessment in health research. BMC medicine. 2017;15(1):202.
49. Jones PJH, Shamloo M, MacKay DS, Rideout TC, Myrie SB, Plat J, et al. Progress and perspectives in plant sterol and plant stanol research. Nutrition reviews. 2018;76(10):725-46.
50. Grover A, Oberoi M, Rehan HS, Gupta LK, Yadav M. Self-reported Morisky Eight-item Medication Adherence Scale for Statins Concords with the Pill Count Method and Correlates


For peer review only - http://bmjopen.bmj.com/site/about/guidelines.xhtml

with Serum Lipid Profile Parameters and Serum HMGCoA Reductase Levels. Cureus. 2020;12(1):e6542-e.

For peer review only

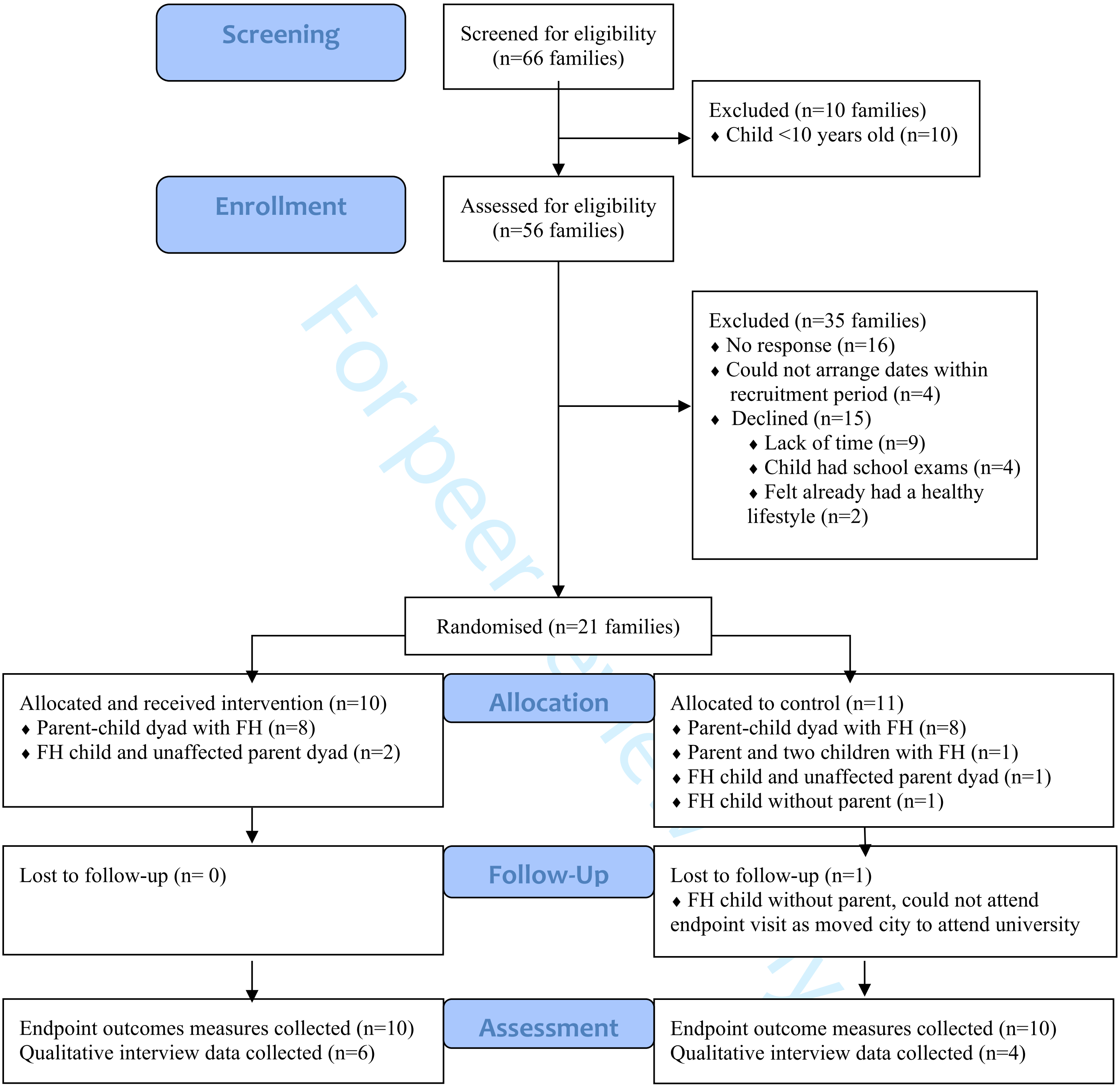


Figure 1. Consolidated Standards of Reporting Trials (CONSORT) diagram of recruitment of families across the three sites.

For peer review only

**Supplementary file 1. CONSORT 2010 checklist of information to include when reporting a pilot or feasibility trial**

| Section/Topic | Item No | Checklist item | Reported on page # |
|---|---|---|---|
| **Title and abstract** | | | |
| | 1a | Identification as a pilot or feasibility randomised trial in the title | Title |
| | 1b | Structured summary of pilot trial design, methods, results, and conclusions (for specific guidance see CONSORT abstract extension for pilot trials) | *1* |
| **Introduction** | | | |
| Background and objectives | 2a | Scientific background and explanation of rationale for future definitive trial, and reasons for randomised pilot trial | 2-3 |
| | 2b | Specific objectives or research questions for pilot trial | 3 |
| **Methods** | | | |
| Trial design | 3a | Description of pilot trial design (such as parallel, factorial) including allocation ratio | 4 |
| | 3b | Important changes to methods after pilot trial commencement (such as eligibility criteria), with reasons | n/a |
| Participants | 4a | Eligibility criteria for participants | 4 |
| | 4b | Settings and locations where the data were collected | 4 |
| | 4c | How participants were identified and consented | 4 |
| Interventions | 5 | The interventions for each group with sufficient details to allow replication, including how and when they were actually administered | 5 and Appendix 2 |
| Outcomes | 6a | Completely defined prespecified assessments or measurements to address each pilot trial objective specified in 2b, including how and when they were assessed | 5-7 |
| | 6b | Any changes to pilot trial assessments or measurements after the pilot trial commenced, with reasons | n/a |
| | 6c | If applicable, prespecified criteria used to judge whether, or how, to proceed with future definitive trial | n/a |
| Sample size | 7a | Rationale for numbers in the pilot trial | 4 |
| | 7b | When applicable, explanation of any interim analyses and stopping guidelines | n/a |
| Randomisation: | | | |
| Sequence | 8a | Method used to generate the random allocation sequence | 4 |

For peer review only

| | | | |
|---|---|---|---|
| generation | 8b | Type of randomisation(s); details of any restriction (such as blocking and block size) | 4 |
| Allocation concealment mechanism | 9 | Mechanism used to implement the random allocation sequence (such as sequentially numbered containers), describing any steps taken to conceal the sequence until interventions were assigned | 4 |
| Implementation | 10 | Who generated the random allocation sequence, who enrolled participants, and who assigned participants to interventions | 4 |
| Blinding | 11a | If done, who was blinded after assignment to interventions (for example, participants, care providers, those assessing outcomes) and how | 4 |
| | 11b | If relevant, description of the similarity of interventions | n/a |
| Statistical methods | 12 | Methods used to address each pilot trial objective whether qualitative or quantitative | 7 |
| **Results** | | | |
| Participant flow (a diagram is strongly recommended) | 13a | For each group, the numbers of participants who were approached and/or assessed for eligibility, randomly assigned, received intended treatment, and were assessed for each objective | 8 and Figure 1 |
| | 13b | For each group, losses and exclusions after randomisation, together with reasons | 8 and Figure 1 |
| Recruitment | 14a | Dates defining the periods of recruitment and follow-up | 8 |
| | 14b | Why the pilot trial ended or was stopped | 8 |
| Baseline data | 15 | A table showing baseline demographic and clinical characteristics for each group | Table 1 |
| Numbers analysed | 16 | For each objective, number of participants (denominator) included in each analysis. If relevant, these numbers should be by randomised group | Tables 2 and 3 |
| Outcomes and estimation | 17 | For each objective, results including expressions of uncertainty (such as 95% confidence interval) for any estimates. If relevant, these results should be by randomised group | Tables 2 and 3 |
| Ancillary analyses | 18 | Results of any other analyses performed that could be used to inform the future definitive trial | 8-10 |
| Harms | 19 | All important harms or unintended effects in each group (for specific guidance see CONSORT for harms) | n/a |
| | 19a | If relevant, other important unintended consequences | n/a |
| **Discussion** | | | |
| Limitations | 20 | Pilot trial limitations, addressing sources of potential bias and remaining uncertainty about feasibility | 11-13 |
| Generalisability | 21 | Generalisability (applicability) of pilot trial methods and findings to future definitive trial and other studies | 11-13 |

For peer review only

| | | | |
|---|---|---|---|
| Interpretation | 22 | Interpretation consistent with pilot trial objectives and findings, balancing potential benefits and harms, and considering other relevant evidence | 11-13 |
| | 22a | Implications for progression from pilot to future definitive trial, including any proposed amendments | 11-13 |
| **Other information** | | | |
| Registration | 23 | Registration number for pilot trial and name of trial registry | Abstract |
| Protocol | 24 | Where the pilot trial protocol can be accessed, if available | 4 |
| Funding | 25 | Sources of funding and other support (such as supply of drugs), role of funders | 15 |
| | 26 | Ethical approval or approval by research review committee, confirmed with reference number | 4 |

For peer review only

**Supplementary file 2. Additional details of intervention content and delivery**

Full details of the development of the intervention, and the selection of content and the delivery method, are described elsewhere.[1] This appendix will provide overview of the 1) The behavioural targets of the intervention; 2) The structure and topics covered in the intervention session; 3) Behaviour change techniques (BCTs) included and details of how they were incorporated into the sessions; 4) The BCTs actually delivered in practice to the participants during the initial and follow-up sessions in the study.

**1. Behavioural targets of the intervention**

All available clinical guidelines for the treatment of children and adults with FH include recommendations for dietary intakes and physical activity. The recommendations are very similar across guidelines, although small differences are apparent. The recommendations selected to be included as targets of the behavioural intervention in this research are summarized below:

1. Total daily fat intake ≤30% of TEI
2. Daily saturated fat intake of ≤10% TEI achieved via replacement with monounsaturated and polyunsaturated fats
3. Daily dietary cholesterol intake ≤300mg
4. Consumption of ≥ 5 portions of fruit and vegetables a day
5. Age appropriate fibre intake: 10-year-olds= 20g/day; 11-16 year-olds = 25g/day and 30g/day for ≥17 years
6. Consumption of 2g of plant sterols or stanols per day
7. Reduce time spent engaged in sedentary behaviors
8. Age appropriate physical activity levels:

- Adults aged 19-64 years: ≥ 150 minutes a week of moderate intensity physical activity or ≥ 75 minutes of vigorous intensity physical activity, or a mixture of the two. Additional activity focussing on improving muscle strength should be undertaken twice a week.
- Children aged 5-18 years: ≥ 60 minutes of moderate-to-vigorous physical activity each day, with 3 of these sessions each week being of vigorous intensity and including activities that strengthen muscle and bone.



For peer review only - http://bmjopen.bmj.com/site/about/guidelines.xhtml

These recommendations reflect the U.K. NICE guidelines,[2] as the developed intervention was to be delivered in an U.K. context, in the first instance. However, three additional recommendations were included for reasons described below:

**A) Consumption of 2g/day plant sterol or stanols**

While the U.K. NICE guidelines do not consider plant sterols and stanols to be an essential component of dietary treatment,[2] a recommendation for their intake was included in the intervention for the following reasons: the promising evidence to support the addition of plant sterols or stanols to the cholesterol lowering diet in children and adults with FH;[3] the indication for use in individuals with FH from the age of six in EAS and AHA clinical guidelines for the management of high cholesterol;[4-6] recommendation for use in other and more recent clinical guidelines for the management of FH;[7-11] their inclusion in the resources produced by *HEART UK: The cholesterol charity*,[12] which are recommended for use in the recent U.K. guidelines for the management of FH in children.[13]

**B) Age appropriate fibre intakes**

All the clinical guidelines advocate a Mediterranean dietary pattern and the high fibre content of this dietary pattern may be of particular benefit to individuals with high cholesterol levels.[14-17] Therefore a specific recommendation for fibre intake was included as a specific goal of dietary treatment in this research, using values derived from the guidelines for fiber intake for the general population in the U.K.[18]

**C) Reduce time spent engaged in sedentary behaviours**

Only the NICE guidelines specify the type or amount of physical activity to be included as part of lifestyle treatment which is based upon the advice for the general population.[2] However, the lifestyle treatment section of the NICE guidelines have not been updated since 2008 and therefore do not include reflect the updated physical activity guidelines for the general population which now include advice to reduce sedentary behaviours.[19] This was therefore added as an additional recommendation to be targeted by the intervention.


For peer review only - http://bmjopen.bmj.com/site/about/guidelines.xhtml

**D) No specific target for trans fatty acids**

While not stated in the U.K. NICE guidelines,[2] other clinical guidelines advocate reducing intake of trans fatty acids (TFA).[8,10-11] This was not selected as a recommendation to be targeted in the intervention in this research for two reasons. Firstly, intakes in the U.K. general population are below the maximum recommended intakes of 2% total energy intake.[20] Secondly, as most TFA are found in processed foods, it is recognised that reducing intakes of TFA requires interventions targeting the use of TFA by the food industry.[21-22] It was therefore not deemed to be appropriate or required for the intervention to target intakes of TFA in the present research. Lastly, in light of the lack of research to support the effectiveness of the current recommendations, it was decided that the intervention would not target additional dietary components that have been investigated to a small extent, such as protein or omega-3 fatty acids.[3]

**2. The structure of, and topics covered in, the intervention sessions**

The intervention was delivered to families by a dietitian as an initial 1-hour face-to-face session followed by four telephone or email follow-up sessions. The intervention primarily focused upon providing education about the dietary and physical activity recommendations and the importance of adhering to these in addition to pharmacological treatment for the avoidance of long-term health consequences associated with FH. The dietary and physical activity recommendations to be targeted in the intervention were communicated as six targets, which provided structure for the intervention booklets and intervention sessions. The targets were:

1) Reduce how many foods high in saturated fat you eat and replace with foods rich in unsaturated fats
2) Limit how many foods high in dietary cholesterol you eat per week
3) Increase the number of portions of fruits and vegetables you eat each day
4) Increase how many insoluble and soluble fiber-rich foods you eat each day
5) Have one plant sterol or stanol fortified yogurt drink each day
6) Increase how physically active you are and reduce time spent being sedentary

Achievable goals were set for each of the six dietary and physical activity targets, tailored towards each individuals current physical activity levels, dietary intakes and unique personal and family characteristics. These goals were SMART (specific, measurable, acceptable, realistic, time-based) in nature.[23] The dietitians provided


For peer review only - http://bmjopen.bmj.com/site/about/guidelines.xhtml

verbal and written (in intervention booklets developed specifically for this trial) instructions on how to achieve these targets such as food swaps, cooking techniques, and recipes. The goals were reviewed at each follow-up session alongside further problem solving and action planning sessions to facilitate continued attainment of goals. Parents and children were encouraged to view each other as sources of emotional and social support and other family members encouraged to adopt the lifestyle changes. Individuals will be encouraged to self-monitor their behaviours through the use of self-reflection diaries and checklists to monitor intake of foods fortified with plant sterols and stanols.

The initial session comprised of eight sections, as shown in Table 1 alongside the intended aims for participants.  Each follow-up session comprised of four sections: *introduction and overview; review of goals; barriers and solutions; further support and follow up*.

3. **Behaviour change techniques (BCTs) included and details of how they were incorporated into the sessions**

Table 2 displays the BCTs to be included in the initial session and Table 3 displays the BCTs to be included in the follow-up sessions. These 'BCT checklists' were provided to dietitians to provide them with instructions on how to incorporate the BCTs into the sessions and to monitor fidelity. Descriptions of each BCT are provided in full in The BCT Taxonomy (V1).[24] Dietitians were instructed to record any adaptations they made to the intended BCTs- including if they delivered other additional BCTs.



For peer review only - http://bmjopen.bmj.com/site/about/guidelines.xhtml

Table 1. The eight sections of the initial intervention session and participant aims

| Section | Aim(s) for participants |
| --- | --- |
| **1. Introduction & 2. Scientific rationale** | • To understand the importance of diet and physical activity in the management of FH and for their overall health.<br>• To be aware of the importance placed on diet and physical activity by national and international guidelines for FH and the current recommendations in England that all FH patients should receive individualised advice about diet & physical activity<br>• To understand that the earlier treatment for FH starts, the more effective it is and this is why it is important to optimise nutrition and physical activity from a young age |
| **3. Education about dietary guidelines** | • To know what a healthy balanced diet looks like, including the food groups and the proportion each one should make to diet<br>• To know what the five diet targets of the intervention are<br>• To understand why each target is important for their health and for the management of their FH<br>• To understand what foods to include/exclude and/or increase/decrease consumption of to achieve targets |
| **4. Education about physical activity guidelines** | • To know what the physical activity and sedentary behaviour recommendations are for their age<br>• To know why these recommendations are important for their health and for management of their FH<br>• To know what the different levels of physical activity are (low, moderate and high) and what types of physical activity fall into which group<br>• To understand how they can incorporate more physical activity into their lifestyle to help increase levels to recommended amounts, or more in addition to reducing sedentary behaviours. |
| **5. Goal setting** | • To agree with dietitian upon SMART goals for each of the targets. These will be changes to their lifestyle that they agree to make over the following 12 weeks to achieve nutritional intakes and physical activity levels closer to the guidelines |
| **6. Barriers and solutions** | • To identify potential barriers that may prevent them from meeting the goals set<br>• To identify, through discussion with dietitian and other family member, solutions to these barriers |
| **7. Follow up arrangements & 8. Wrap-up** | • To understand what will happen over the following 12 weeks<br>• To understand the purpose of the weekly reflection diaries and know how to fill them out<br>• To know what will be discussed during the follow-up sessions<br>• An opportunity to ask any unanswered questions<br>• To receive summary of session discussion & receive encouragement and motivation from dietitian |

For peer review only

**Table 2. BCT checklist for initial session of intervention to be completed by dietitians**

| Behaviour change technique | How to be used within intervention | Included? |
|---|---|---|
| Action planning | Individual will be prompted to develop specific planning of how they will achieve each goal set i.e. *if increasing fibre intake then the food swap or additional food to be included in diet will be specified, along with what meal or snack they will include it in and how many times per day or week.* | |
| Behaviour substitution | Individuals encouraged to set goals that involve swapping something they currently do everyday with something that will help them achieve the guidelines to help establish new healthy habits i.e. *walking to and from work instead of driving* or *always having a piece of fruit when they make their morning cup of tea instead of a biscuit*. | |
| Behavioural practice/rehearsal | Prompt individual to practice cooking during intervention and adults to encourage child to help them. | |
| Biofeedback | Individual to be provided with weight, body fat % and blood pressure before starting the intervention to prompt adoption of guidelines- either to improve these figures or maintain them. | |
| Comparative imaging of future outcomes | Dietitian to prompt individual to think about what the possible health outcomes would be if they choose to follow guidelines compared to if they chose not to- with emphasis on what this would mean for their children or parents. | |
| Credible sources | • Intervention delivered by a dietitian who will explain the training they have undertaken to gain that title- to help individual recognise that their advice is credible. All individuals will also be informed that their doctor is aware and supportive of them receiving the intervention as they view it as being part of their clinical care.<br>• Individual to be receive verbal and written advice about the guidelines which will include information about where the guidelines have come from- national committees across several countries who have reviewed all the available scientific evidence and come to same conclusion about the guidelines that individuals with FH should be following. | |
| Demonstration of the behaviour | Individual to be signposted to resources which can aid with demonstration of the behaviour i.e. *step by step recipe videos or pictorial guides and parents encouraged to demonstrate cooking skills to child.* | |
| Feedback on outcomes of behaviour | Individual to receive feedback about how their dietary intakes and physical activity levels compare to the guidelines at the start and end of the intervention as evidence of their capability of adhering to the guidelines to promote maintenance of behaviours after intervention ended.<br>Note- the follow-up information will be delivered at research contact 3. Provide initial information in initial session. | |
| Framing/re-framing | • Guidelines communicated to individual as being specifically for individuals with FH as opposed to general healthy lifestyle guidelines provided to all individuals. The inclusion of two specific dietary guidelines for individuals with FH such as eating foods fortified with plant stanols/sterols and reducing dietary cholesterol intakes will help individuals to buy into the idea that following the guidelines is part of their identity of having FH. The benefits of following the guidelines in the management of their FH will be emphasised, in addition to general overall health benefits.<br>• Dietary guidelines to be communicated as a healthy lifestyle rather than a restrictive diet, with all foods permitted. Emphasis will be put on foods to add into the diet (fruits, vegetables, fibre rich foods, plant sterols/stanols) to help individual view dietary choices are positive and enjoyable.<br>• Physical activity to be communicated positively, with emphasis placed upon finding activities that the individual enjoys doing, either alone or with friends or family, rather than it being a chore they have to try and fit into their day without enjoying it.<br>• Individual prompted to view the guidelines as behaviours that can help reduce their risk of developing symptoms as their family members have, or reduce the likelihood of experiencing further symptoms. They are something the individual can do to help take control of their health. | |
| Goal setting (behavioural) | • Individual prompted to set their own goals which they feel are achievable-which take into consideration their food preferences, fitness levels, readiness to change and lifestyles. The goals will be SMART. Parents will be encouraged to help child with setting their goals. | |
| Graded tasks | • Child to be prompted to help with food preparation, starting with simple task such as preparing vegetables, progressing to more complex tasks until able to prepare full meal.<br>• Adults to be prompted to start cooking meals- starting with simple recipes and progressing to more complex ones. | |
| Identification of self as a role model | • Parents encouraged to view themselves as role models for their children and make their behaviours part of everyday life for their children to help them foster adoption of healthy habits from a young age. Dietitian to communicate the benefits engaging in the behaviours could bring to these significant others.<br>• Children also encouraged to view themselves as role models for their parents, other siblings and friends. | |
| Information about antecedents | Individual prompted to consider specific contexts in which they find it hard to adhere to dietary guidelines and identify triggers for this behaviour. The findings will be addressed in action planning and problem-solving session of the intervention. | |

| | | |
|---|---|---|
| Information about emotional consequences | • Dietitian to discuss with individual the potential improvement in their mood that they could experience if they choose to engage in dietary behaviours.<br>• Dietitian to emphasis to individual that if they find foods that they enjoy that fit the guidelines, they will also experience the same enjoyment of these foods. | |
| Information about health consequences | • The intervention starts with explanation from dietitian about the importance of lifestyle guidelines in the management of FH. It will be explained that despite use of medication, many individuals with FH may still be at higher risk of cardiovascular disease and adherence to lifestyle guidelines can help reduce this risk. The benefits to their overall health will also be communicated.<br>• Individuals to be informed about the 'silent' nature of cholesterol and the importance of keeping cholesterol low for your whole life, before any symptoms occur. | |
| Instruction on how to perform behaviour | • Individuals will receive detailed instructions about food swaps and cooking methods. The advice given will provide instructions to individual about how they can achieve the desired behaviours in their current *context i.e. cheaper options for suggested food swaps, suggested food swaps one they can obtain in the current place they shop, easy and quick options for meals if individual has limited time to cook and suggestions of physical activity they can fit into their current routines such as walking instead of bus/car.*<br>• The dietary intake data will be analysed ahead of intervention to establish any existing eating habits that are indicative of misinformation i.e. *cooking with coconut oil.* | |
| Problem solving | During intervention 'barriers and solutions' section, dietitian will encourage individual to think of situations in which they feel they will struggle to engage in the desired behaviours (*i.e. social situations*) and think of solutions to overcome these. | |
| Prompts and cues | • Individual advised to leave intervention booklet in a place they regularly eat to prompt them to engage in the dietary goals.<br>• Set reminders on phones/fitness monitors to move regularly. | |
| Restructuring the physical environment | • Advise individual to keep food choices they enjoy eating, that are in line with the guidelines, in the house/at work/in car to encourage consumption of these<br>• Individuals encouraged to socialise with friends and family in places that facilitate engagement of desired behaviours i.*e. meet in the park* or *choose restaurants in which there are suitable options for them.* | |
| Restructuring the social environment | The intervention is delivered at a family-based level with parent and child making dietary and physical activity choices together. Any other family members will also be encouraged to make the changes as well to facilitate a home environment that encourages adherence to the dietary and physical activity guidelines. | |
| Social support (emotional) | • Dietitian to provide emotional support during intervention- discussing with them what else is going on in their life and how this is influencing their ability to adhere to guidelines.<br>• Individual also encouraged to seek support from the family member(s) they are taking part in the intervention with- to view it as a 'team effort' and provide support and encouragement to each other.<br>• Dietitian to encourage parent and child to provide support to each other throughout the intervention and encourage each other to engage in the guidelines.<br>• Individuals also encouraged to seek the emotional support of friends and family. | |
| Social support (practical) | • Dietitian to provide practical support during the session- helping individual to identify methods to help encourage the behaviour *i.e. online recipes or suggestions on how they could obtain practical support from friends* or *having their parent prepare help make their lunch.*<br>• The dietitian will encourage the whole family (both with and without FH) to engage in the dietary and physical activity guidelines to provide support to the individuals.<br>• Individuals encouraged to seek support from family members (including those not taking part in intervention) and friends i.*e. arranging to go to the gym with a friend or having partner/sibling help with food shopping or meal preparation.* | |
| Verbal persuasion about capabilities | Dietitian to encourage individual and tell them that they are capable of changing their behaviours. | |
| Self-monitoring of behaviour (prompt) | Individuals asked to complete weekly reflection diaries in which they record whether they have been able to meet the goals set for lifestyle behaviours.<br>They will also be asked to keep checklists of plant stanol/sterol drink consumption. | |

**Table 3**. **BCT checklist for intervention follow-up sessions completed by dietitians**

| Behaviour change technique | Instructions for use within intervention | Included? |
|---|---|---|
| Action planning | • Individual will be prompted to develop specific planning of how they will continue to, or start to, achieve each goal set at previous and current session i.e. if increasing fibre intake then the food swap or additional food to be included in diet will be specified, along with what meal or snack they will include it in and how many times per day or week.<br>• Follow up sessions are opportunity to review action plans set a previous session and identify what has worked and what needs more planning. | |
| Problem solving | • During 'barriers and solutions' section, dietitian will review with individual the barriers and solutions they discussed at previous session. Together they will identify what they will do next if barrier still preventing them from achieving goal.<br>• Dietitian will encourage individual to think of other situations in which they feel they will struggle to engage in the desired behaviours between now and next follow up session and think of solutions to overcome these. | |
| Social support (emotional) | • Dietitian to provide emotional support during follow-up sessions- discussing with them what else is going on in their life and how this is influencing their ability to adhere to guidelines.<br>• Individuals encouraged to seek the emotional support of friends and family<br>• Individual encouraged to seek support from the family member(s) they are taking part in the intervention with- to view it as a 'team effort' and provide support and encouragement to each other to engage in the guidelines | |
| Social support (practical) | • Dietitian provide practical support during follow-up sessions- helping individual to identify methods to help encourage the behaviour i.e. links to online resources or suggestions on how they could obtain practical support from friends or family i.e. having their parent prepare help make their lunch<br>• Individuals encouraged to seek support from family members (including those not taking part in intervention) and friends i.e. arranging to go to the gym with a friend or having partner/sibling help with food shopping or meal preparation. | |
| Verbal persuasion about capabilities | Dietitian to encourage individual and tell them that they are capable of changing their behaviours. | |
| Focus on past success | Dietitian to focus on any successful changes individuals have made and use these to provide encouragement and motivation to individual to carry on and make more changes. | |
| Review behavioural goal(s) | At each follow-up session, dietitian and individual will review the goals set at previous session or follow-up. Together they will agree to either: keep goal the same, modify the goal or create new goal. These decisions will be based upon individuals levels of achievement and willingness to change. | |
| Self-monitoring of behaviour (prompt to) | Individuals asked to complete weekly reflection diaries in which they record whether they have been able to meet the goals set for lifestyle behaviours which will be discussed during follow-ups. They will also be asked to keep checklists of plant stanol drink consumption which will be reviewed at each follow up session. | |
| Self-monitoring of behaviour (as reported by individuals) | Individuals self-reported completion of the weekly self-reflection diaries and plant stanol checklist. | |


For peer review only - http://bmjopen.bmj.com/site/about/guidelines.xhtml

4. The BCTs actually delivered in practice to the participants during the initial and follow-up sessions.

The BCTs intended to have been delivered in the initial and follow up sessions are indicated with a tick in Table 4 alongside the percentage of families who received each one in practice. Further details of how each BCT was to be delivered in the intervention are displayed in Table 2 and Table 3. Overall, the fidelity was high with the majority of BCTs delivered as intended across all sessions and sites. Four BCTs (*demonstration of the behaviour*; *graded tasks*, *problem solving* and *self-monitoring of behaviour prompt)* were not delivered to all families. In all instances, this was reported by the dietitians to be due to it not being applicable to the individual family. For instance, the dietitian reported it was not appropriate to prompt families, who had previously stated they were not, or did not want to, use the self-reflection resources at previous follow-up sessions. The families stated they did not have time or did not feel it was necessary to help them achieve their goals. Two further BCTs *biofeedback* and *feedback on outcomes of behaviour* were not delivered to families (n=2) at the London study site as the dietitian did not have access to the dietary intake or anthropometric data ahead of the initial session.

In response to the needs of each individual family, the dietitians reported the occasional use of additional BCTs during the follow-up sessions, as indicated by the plus sign in Table 4. The dietitians reported using these BCTs to help families overcome barriers they were facing in achieving their goals. For instance, *instruction on how to perform the behaviour* was delivered to 20% of families during follow-up one who had requested recipes or cooking advice.



For peer review only - http://bmjopen.bmj.com/site/about/guidelines.xhtml

Table 4. The BCTs intended to be included in the initial and follow-up sessions (✔), the additional BCTs delivered (+) and the percentage of families who received each BCT in each session (%)

| Behaviour change technique (BCT)* † | Initial session (n=10) | Follow-up 1 (n=10) | Follow-up 2 (n=10) | Follow-up 3 (n=10) | Follow-up 4 (n=10) |
|---|---|---|---|---|---|
| **Action planning** | ✔ (100%) | ✔ (100%) | ✔ (100%) | ✔ (100%) | ✔ (100%) |
| **Behaviour substitution** | ✔ (100%) | + (20%) | + (10%) | + (5%) | |
| **Behavioural practice/rehearsal** | ✔ (100%) | | | | |
| **Biofeedback** | ✔ (80%) | | | | |
| **Comparative imaging of future outcomes** | ✔ (100%) | | | | |
| **Credible sources** | ✔ (100%) | | | | |
| **Demonstration of the behaviour** | ✔ (0%) | + (5%) | | | |
| **Feedback on outcomes of behaviour** | ✔ (90%) | | | | |
| **Framing/re-framing** | ✔ (100%) | + (25%) | + (25%) | + (5%) | |
| **Goal setting (behavioural)** | ✔ (100%) | | | | |
| **Graded tasks** | ✔ (30%) | | | | |
| **Identification of self as a role model** | ✔ (100%) | | | | |
| **Information about antecedents** | ✔ (100%) | | | | |
| **Information about emotional consequences** | ✔ (100%) | | | | |
| **Information about health consequences** | ✔ (100%) | | | | |
| **Instruction on how to perform behaviour** | ✔ (100%) | + (20%) | + (10%) | + (11%) | |
| **Problem solving** | ✔ (100%) | ✔ (100%) | ✔ (100%) | ✔ (84%) | ✔ (100%) |
| **Prompts and cues** | ✔ (100%) | | + (5%) | + (5%) | |
| **Restructuring the physical environment** | ✔ (100%) | + (10%) | | | |
| **Restructuring the social environment** | ✔ (100%) | | | | |
| **Social support (emotional)** | ✔ (100%) | ✔ (100%) | ✔ (100%) | ✔ (100%) | ✔ (100%) |
| **Social support (practical)** | ✔ (100%) | ✔ (100%) | ✔ (100%) | ✔ (100%) | ✔ (100%) |
| **Verbal persuasion about capabilities** | ✔ (100%) | ✔ (100%) | ✔ (100%) | ✔ (100%) | ✔ (100%) |
| **Focus on past success** | | ✔ (100%) | ✔ (100%) | ✔ (100%) | ✔ (100%) |
| **Review behavioural goal(s)** | | ✔ (100%) | ✔ (100%) | ✔ (100%) | ✔ (100%) |
| **Self-monitoring of behaviour (prompt to)** | ✔ (90%) | ✔ (100%) | ✔ (100%) | ✔ (68%) | ✔ (50%) |

*Details of how each BCT was delivered in the intervention are available in Table 2 and Table 3.
† Further details of the BCTs are available on the BCT taxonomy (BCTTv1)[24]

For peer review only

References

1.Kinnear FJ, Wainwright E, Bourne JE, Lithander FE, Hamilton-Shield J, Searle A. The development of a theory informed behaviour change intervention to improve adherence to dietary and physical activity treatment guidelines in individuals with familial hypercholesterolaemia (FH). BMC health services research. 2020;20(1):27.
2.NICE clinical guideline 71: Familial hypercholesterolemia: identification and management. [Internet]. 2008 [cited 19/01/2018]. Available from: http://nice.org.uk/guidance/cg71.
3.Malhotra A, Shafiq N, Arora A, Singh M, Kumar R, Malhotra S. Dietary interventions (plant sterols, stanols, omega-3 fatty acids, soy protein and dietary fibers) for familial hypercholesterolaemia. Cochrane Database Syst Rev. 2014(6):CD001918.
4.Gylling H, Plat J, Turley S, Ginsberg HN, Ellegard L, Jessup W, et al. Plant sterols and plant stanols in the management of dyslipidaemia and prevention of cardiovascular disease. Atherosclerosis. 2014;232(2):346-60.
5.Mach F, Baigent C, Catapano AL, Koskinas KC, Casula M, Badimon L, et al. 2019 ESC/EAS Guidelines for the management of dyslipidaemias: lipid modification to reduce cardiovascular risk: The Task Force for the management of dyslipidaemias of the European Society of Cardiology (ESC) and European Atherosclerosis Society (EAS). European heart journal. 2019;41(1):111-88.
6.Grundy Scott M, Stone Neil J, Bailey Alison L, Beam C, Birtcher Kim K, Blumenthal Roger S, et al. 2018 AHA/ACC/AACVPR/AAPA/ABC/ACPM/ADA/AGS/APhA/ASPC/NLA/PCNA Guideline on the Management of Blood Cholesterol. Circulation.0(0):CIR.0000000000000625.
7.Goldberg AC, Hopkins PN, Toth PP, Ballantyne CM, Rader DJ, Robinson JG, et al. Familial hypercholesterolemia: screening, diagnosis and management of pediatric and adult patients: clinical guidance from the National Lipid Association Expert Panel on Familial Hypercholesterolemia. J Clin Lipidol. 2011;5(3 Suppl):S1-8.
8.Nordestgaard BG, Chapman MJ, Humphries SE, Ginsberg HN, Masana L, Descamps OS, et al. Familial hypercholesterolaemia is underdiagnosed and undertreated in the general population: guidance for clinicians to prevent coronary heart disease : Consensus Statement of the European Atherosclerosis Society. European heart journal. 2013;34(45):3478-90.
9.Wiegman A, Gidding SS, Watts GF, Chapman MJ, Ginsberg HN, Cuchel M, et al. Familial hypercholesterolaemia in children and adolescents: gaining decades of life by optimizing detection and treatment. European heart journal. 2015;36(36):2425-37.
10.Watts GF, Gidding S, Wierzbicki AS, Toth PP, Alonso R, Brown WV, et al. Integrated guidance on the care of familial hypercholesterolaemia from the International FH Foundation. International journal of cardiology. 2014;171(3):309-25.
11.Gidding SS, Champagne MA, de Ferranti SD, Defesche J, Ito MK, Knowles JW, et al. The Agenda for Familial Hypercholesterolemia: A Scientific Statement From the American Heart Association. Circulation. 2015;132(22):2167-92.
12.HEART UK: The Cholesterol Charity. Familial hypercholesterolaemia: An education booklet for people with familial hypercholesterolaemia (FH). London HEART UK: The cholesterol charity; 2019.
13.Ramaswami U, Humphries SE, Priestley-Barnham L, Green P, Wald DS, Capps N, et al. Current management of children and young people with heterozygous familial hypercholesterolaemia - HEART UK statement of care. Atherosclerosis. 2019;290:1-8.



For peer review only - http://bmjopen.bmj.com/site/about/guidelines.xhtml

14.McRae MP. Dietary Fiber Is Beneficial for the Prevention of Cardiovascular Disease: An Umbrella Review of Meta-analyses. Journal of Chiropractic Medicine. 2017;16(4):289-99.
15.Brown L, Rosner B, Willett WW, Sacks FM. Cholesterol-lowering effects of dietary fiber: a meta-analysis. The American journal of clinical nutrition. 1999;69(1):30-42.
16.Whitehead A, Beck EJ, Tosh S, Wolever TM. Cholesterol-lowering effects of oat β-glucan: a meta-analysis of randomized controlled trials. The American journal of clinical nutrition. 2014;100(6):1413-21.
17.Ho HV, Sievenpiper JL, Zurbau A, Blanco Mejia S, Jovanovski E, Au-Yeung F, et al. The effect of oat β-glucan on LDL-cholesterol, non-HDL-cholesterol and apoB for CVD risk reduction: a systematic review and meta-analysis of randomised-controlled trials. Br J Nutr. 2016;116(8):1369-82.
18.Nutrition SACo. Carbohydrates and Health. London: The Stationery Office (TSO); 2015.
19.Department of Health and Social Care. UK Chief Medical Ofiicers' Physical Activity Guidelines London: UK Government Department of Health and Social Care; 2019.
20.Public Health England. National Diet and Nutrition Survey Results from Years 7 and 8 (combined) of the Rolling Programme (2014/2015 to 2015/2016). London: Public Health England and Food Standards Agency 2018.
21.Hyseni L, Bromley H, Kypridemos C, O'Flaherty M, Lloyd-Williams F, Guzman-Castillo M, et al. Systematic review of dietary trans-fat reduction interventions. Bulletin of the World Health Organization. 2017;95(12):821-30G.
22.Hendry VL, Almíron-Roig E, Monsivais P, Jebb SA, Neelon SE, Griffin SJ, et al. Impact of regulatory interventions to reduce intake of artificial trans-fatty acids: a systematic review. American journal of public health. 2015;105(3):e32-42.
23.Ogbeiwi O. Why written objectives need to be really SMART. British Journal of Healthcare Management. 2017;23(7):324-36.
24.Michie S, Richardson M, Johnston M, Abraham C, Francis J, Hardeman W, et al. The behavior change technique taxonomy (v1) of 93 hierarchically clustered techniques: building an international consensus for the reporting of behavior change interventions. Ann Behav Med. 2013;46(1):81-95.

For peer review only

**Supplementary file 3. Additional details of outcome measures used in study**

*Behavioural outcomes (study objectives 5 & 6)*

Behavioural outcomes were collected in the week preceding research contact two (baseline) and research contact three (endpoint).

1. Dietary intakes: participants recorded their dietary intake on four non-consecutive days (including one weekend day) using Intake24, a validated online 24-hour recall tool.[1,2] Data were downloaded from Intake24 as an Excel file before being cleaned and analysed, in line with Intake24 data processing guidelines. After data quality checks, data not meeting the validity criteria (completion rate <2 minutes; daily energy intake <2000KJ or >16000KJ) were excluded and only participants with two weekdays and one weekend day of valid intake were included in the analysis. Using pivot tables, daily intakes of macronutrients were calculated. Intakes of fruits and vegetables were presented as mean portions consumed per day by dividing the sum intake of fruit and vegetables by 80, per participant and per day. The value of 80 was chosen as a single portion of fruit and vegetables, in U.K. national guidelines, is considered to be 80g. Dried fruit, fruit juices and pulses were included but a portion was considered to be 30g, 150ml and 80g respectively, and fruit juices and pulses were capped at one portion per day. Fruits and vegetables in composite dishes were not included as there is not yet a validated method to calculate this using the Intake24 database. In line with the specific dietary goals of the intervention described in appendix 2, mean daily intakes of fat, saturated fat, monounsaturated fats, polyunsaturated fats, cholesterol, fiber, fruit and vegetable portions, and plant sterol or stanol fortified foods were chosen as outcome measures.

2. Physical activity levels: average daily minutes of free-living moderate and vigorous physical activity (MVPA) was measured by asking participants to wear an ActiGraph GT3X+ accelerometer monitor (ActiGraph, Pensacola, USA) during waking hours for seven consecutive days. Participants also completed logbooks to capture waking/sleeping times and any activities during which the monitor was taken off. Raw data were downloaded and analysed, in 15 and 60-second epochs for children and adults respectively, using the manufacturer's software (ActiLife software v6.13.4; ActiGraph, Pansacola, FL, USA). Logbook data and an algorithm, which detected non-wear as periods of 60 minutes or more of consecutive zeros with no allowance

For peer review only

for interruptions, was used to identify valid weartime periods. A second researcher (AET) analysed the data from 10 participants (five adults and five children) to ensure consistency and validity of the decisions made by FJK. To determine MVPA, the cut-points developed by Freedson[3] and Evenson[4] were used for adults and children, respectively. At least four valid days (defined as ≥ 8 hours weartime), including at least one weekend day, were required for analysis.

3. Sedentary time: participants wore an activPAL3 accelerometer (PAL Technologies Ltd., Glasgow, UK) for seven consecutive days to estimate the average daily time spent sedentary (sitting or lying).[5] Accelerometers were made waterproof using a nitrile sleeve and were attached to the front of the thigh using a waterproof hypoallergenic medical dressing (Hypafix), enabling participants to wear the monitor continuously for 24 hours/day, removing only for swimming. Data was downloaded using the manufacturer's software (PAL software v7.2.38; PAL Technologies Ltd., Glasgow, UK) and was cleaned and processed in ProcessingPAL, a Java application which utilises a validated algorithm to isolate waking weartime.[6] Logbooks were used to aid identification of the non-wear periods and sleep time, which were removed from analysis. A second researcher (AET) analysed the data from 10 participants (five adults and five children) to ensure consistency and validity of the decisions made by FJK. At least four valid days (defined as ≥10 h of worn waking hours, <95% of time spent in any one position and ≥500 steps), including one weekend day was required for analysis.

For peer review only

Table 1. Data processing and analysis decisions for data collected using Actigraph accelerometers

| Factor to consider | Description | Approach taken in this study: Adults | Approach taken in this study: Children |
|---|---|---|---|
| **EPOC length** | EPOC length is the time period over which the ActiGraph counts are totalled and can range between 1 and 60 seconds. | • 60 second epochs | • 15 second epochs |
| **Cut-points for classification of activity intensity** | To interpret the counts per epoch, thresholds are applied to define different intensities of PA known as cut-points. There are several sets of cut-points available. | • Freedson et al[3] | • Evenson et al[4] |
| **Classification of non-wear time** | Periods of time that participant is not wearing Actigraph should be detected and deleted from the data to avoid misclassifying non-wear time as sedentary time. There are a variety of available algorithms to detect non-wear time, which differ in the criteria they use. | • An algorithm which detects non-wear as periods of 60 minutes or more of consecutive zeros with no allowance for interruptions<br>• Participants are asked to complete logbooks to capture non-wear occasions and reasons, to cross check results from algorithm<br>• Self-reported PA during non-wear times (e.g. swimming or contact sports) were not included in analysis | |
| **Valid day & minimum number of days criteria** | The number of hours of weartime required to define a valid day, and the number of these valid days required for data to be included in the analysis. | • A valid day is defined as ≥ 8 hours (480 minutes)<br>• Four valid days (including one weekend day) were required for inclusion in analysis | |
| **Uni- or tri-axial counts** | The ActiGraph detects activity counts across three axes (vertical, mediolateral and anteroposterior). Researchers can choose to use the vector magnitude (VM) score, which includes activity counts detected across all axes, or use activity counts detected across vertical axis only. | • Activity counts detected across vertical axis were analysed to match the approach used in the studies that developed the MVPA cut-points | |



For peer review only - http://bmjopen.bmj.com/site/about/guidelines.xhtml

*Clinical outcomes (Study objectives 5 and 6)*

Clinical outcomes were collected from participants at research contact two (baseline) and three (endpoint) by members of the research team.

1. Anthropometry and body composition: Height was measured using a stadiometer and weight, body fat percentage and fat free mass using bioelectrical impedance scales (Tanita™ MC-780MA). At sites where this equipment was unavailable, weight was measured using medical scales. These outcomes were used to calculate the body mass index (BMI) for adult participants. The LMS method was used to express the BMIs of child participants as BMI Z-scores and percentiles.[7] This was carried out using the lmsGrowth excel add-in[8] and the British 1990 reference population.[9] The 91$^{st}$ and 98$^{th}$ BMI percentiles were applied to define overweight and obesity as recommended for clinical interventions.[10]

2. Resting arterial blood pressure: The mean of two sphygmomanometer readings (three if 1$^{st}$ and 2$^{nd}$ differed by more than 10mmHg) was calculated to provide systolic and diastolic blood pressure outcomes. Participants were seated during the measurements, which were taken after a period of at least five minute of rest to allow values to return to resting levels.

3. HRQoL: Children completed an age appropriate Pediatric Quality of Life Inventory ™ (PedsQL™) Version 4.0 and adults completed an EuroQol Group EQ-5D-3L health questionnaire.[11,12] For children, the completed inventories were scored using the recommended scoring system[13] to produce a score for HRQoL and a breakdown of the component physical and psychosocial (emotional, social and school) functioning scores. For adults, the recommended scoring system was used[12] to produce a five-digit health state profile representative of five dimensions of health which was converted into a single index value using a validated value set created for use in the United Kingdom.[14] The visual analogue scale data was presented as a single value between 1 and 100.

4. Blood lipid profile: 25ml fasted blood samples were collected and a proportion was analysed immediately in the local NHS laboratory. Analysis was done by routine homogeneous enzymatic methods using Cobas reagents (Cholesterol Gen 2;


For peer review only - http://bmjopen.bmj.com/site/about/guidelines.xhtml

Triglyceride Gen 1; HDL Gen 3 or 4) on Roche/Hitachi c701/2 analysers. LDL-C concentration was then calculated using the Friedewald equation.[15] The remaining sample was processed and stored at -80°C as plasma or serum. Subsequent batch metabolomics analysis to determine 201 metabolomics is planned, but not presented in this manuscript due to laboratory closure during the COVID-19 outbreak.

References

1.Simpson E, Bradley J, Poliakov I, Jackson D, Olivier P, Adamson AJ, et al. Iterative Development of an Online Dietary Recall Tool: INTAKE24. Nutrients. 2017;9(2):118.
2.Bradley J, Simpson E, Poliakov I, Matthews J, Olivier P, Adamson A, et al. Comparison of INTAKE24 (an Online 24-h Dietary Recall Tool) with Interviewer-Led 24-h Recall in 11–24 Year-Old. Nutrients. 2016;8(6):358.
3.Freedson PS, Melanson E, Sirard J. Calibration of the Computer Science and Applications, Inc. accelerometer. Medicine & Science in Sports & Exercise. 1998;30(5):777-81.
4.Evenson KR, Catellier DJ, Gill K, Ondrak KS, McMurray RG. Calibration of two objective measures of physical activity for children. J Sports Sci. 2008;26(14):1557-65.
5.Atkin AJ, Gorely T, Clemes SA, Yates T, Edwardson C, Brage S, et al. Methods of Measurement in epidemiology: Sedentary Behaviour. International Journal of Epidemiology. 2012;41(5):1460-71.
6.Winkler EA, Bodicoat DH, Healy GN, Bakrania K, Yates T, Owen N, et al. Identifying adults' valid waking wear time by automated estimation in activPAL data collected with a 24 h wear protocol. Physiological measurement. 2016;37(10):1653-68.
7.Green PJ, Cole TJ. Smoothing reference centile curves: the LMS method and penalized likelihood. Statistics in Medicine. 1992;11:1305-19.
8.Pan HaC, T User's guide to lmsGrowth. London, U.K. : Medical Research Council; 2012.
9.Cole TJ, Freeman JV, Preece MA. Body mass index reference curves for the UK, 1990. Archives of Disease in Childhood. 1995;73(1):25.
10.Scientific Advisory Committee on Nutrition (SACN). SACN/RCPCH defining child underweight, overweight and obesity in the UK In: England PH, editor. London: Public Health England; 2012.
11.Rabin R, de Charro F. EQ-5D: a measure of health status from the EuroQol Group. Ann Med. 2001;33(5):337-43.
12.EuroQoL Research Foundation. EQ-5D-3L User Guide Basic information on how to use the EQ-5D-3L instrument. Version 6.0. . 2018.
13.Varni JW. Scaling and scoring of the Pediatric Quality of Life Inventory ™ PedsQL ™. 2017 [Available from: http://www.pedsql.org/PedsQL-Scoring.pdf.
14.Dolan P. Modeling valuations for EuroQol health states. Medical care. 1997;35(11):1095-108.
15.Friedewald WT, Levy RI, Fredrickson DS. Estimation of the concentration of low-density lipoprotein cholesterol in plasma, without use of the preparative ultracentrifuge. Clin Chem. 1972;18(6):499-502



For peer review only - http://bmjopen.bmj.com/site/about/guidelines.xhtml
